# Bacterial cell division protein FtsZ complexes with a phage protein to activate bacterial immunity

**DOI:** 10.1038/s41564-026-02384-6

**Published:** 2026-06-12

**Authors:** Tong Zhang, Anastasiia Nadieina, Carl B. W. Soderstrom, Julia E. Ramseyer, Christina R. Beck, Kyo Coppieters ‘t Wallant, Chloé Martens, Abel Garcia-Pino, Michael T. Laub

**Affiliations:** 1 Department of Biology, Massachusetts Institute of Technology, Cambridge, MA USA; 2 Cellular and Molecular Microbiology, Faculté des Sciences, Université libre de Bruxelles, Brussels, Belgium; 3 Biochemistry and Structural Biology, Université libre de Bruxelles, Brussels, Belgium; 4 WELRI: WEL Research Institute, Wavre, Belgium; 5 Howard Hughes Medical Institute, Massachusetts Institute of Technology, Cambridge, MA USA

**Keywords:** Phage biology, Microbial genetics

## Abstract

Many antiphage defence systems directly bind a specific phage-encoded protein that acts similar to a pathogen-associated molecular pattern to activate an immune response. Such activation is often assumed to occur independent of host factors. Here we demonstrate that the antiphage defence protein CapRel^Ebc^, a fused toxin–antitoxin system from *Enterobacter chengduensis*, senses the T7 phage-encoded protein Gp0.4 in complex with the host bacterial factor FtsZ, an essential cell division protein. During T7 infection, Gp0.4 sequesters monomeric FtsZ to block its polymerization and thereby inhibit bacterial cell division. Only the complex of Gp0.4–FtsZ, but neither protein alone, triggers CapRel^Ebc^ activity. Structural modelling and hydrogen–deuterium exchange mass spectrometry indicate that Gp0.4, FtsZ and CapRel^Ebc^ form a ternary complex that activates phage defence. Our work suggests that activation of bacterial immune systems does not always depend exclusively on phage-encoded triggers. Instead, activation can involve host factors targeted by phages, analogous to how eukaryotic innate immune systems detect pathogen-induced perturbations of host cells through effector-triggered immunity.

## Abstract (web summary)

The activation of an antiphage defence system relies on host factors targeted by phages, a mechanism analogous to the way that eukaryotic innate immune systems detect pathogen-induced perturbations of host cells through effector-triggered immunity.

## Main

Bacteria have evolved diverse innate immune systems to protect themselves against phage infection^1–6^. Recently, numerous antiphage defence systems have been discovered, but how these systems sense phage infection remains poorly understood. Analogous to how eukaryotic innate immune systems use pattern recognition receptors to recognize specific pathogen-associated molecular patterns (PAMPs), some bacterial defence systems sense PAMP-like molecules from phages and then trigger an immune response^7,8^. In addition to PAMP-triggered immunity, eukaryotic innate immune systems can also detect pathogens through ‘effector-triggered immunity’ (ETI), which involves detection of virulence-associated activities of pathogens^9–11^. Many eukaryotic pathogens encode effectors that disrupt host cellular functions to promote their own replication, and such activities can be sensed by the host immune system as a way of detecting infection^9,11^.

Here, we investigate the activation of an antiphage protein called CapRel^Ebc^ from *Enterobacter chengduensis*. CapRel systems are broadly distributed toxin–antitoxin systems that often have the toxin and antitoxin fused into a single polypeptide^12^. The toxin domains of such CapRels are homologous to the bacterial alarmone synthetases that pyrophosphorylate purine nucleotides including GTP, ATP and the adenosine at the 3′ ends of some tRNAs^13–16^. We previously showed that the homologue CapRel^SJ46^ in *Escherichia coli* can sense phage infection by directly binding to the major capsid protein or the small protein Gp54 produced by some phages^12,17^, similar to PAMP-based sensing in eukaryotes. Binding of these phage-encoded triggers relieves auto-inhibition of the CapRel^SJ46^ toxin and enables it to pyrophosphorylate tRNAs to inhibit translation and restrict phage propagation^12,17^.

The homologue CapRel^Ebc^ protects *E. coli* against different phages compared with CapRel^SJ46^, and it is unknown how CapRel^Ebc^ senses phage infection. We now demonstrate that CapRel^Ebc^ is activated during T7 phage infection when it forms a ternary complex with the phage protein Gp0.4 and its cellular target, the essential cell division protein FtsZ^18^. Notably, searches for the triggers of antiphage defence systems often focus exclusively on phage-encoded factors, but our findings indicate that host factors can also have an essential role.

## Results

### CapRel^Ebc^ is activated by Gp0.4 from phage T7

**Fig. 1:**
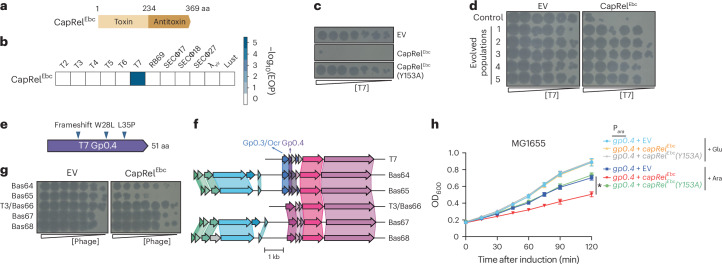
CapRel^Ebc^ is activated by Gp0.4 of phage T7. **a**, A schematic of the domain organization of CapRel^Ebc^. **b**, EOP data for the phages indicated when infecting cells producing CapRel^Ebc^. **c**, Serial, tenfold dilutions of T7 phage spotted on lawns of cells harbouring an empty vector (EV) or a plasmid producing CapRel^Ebc^ (wild type or the Y153A variant). Relative phage concentration is indicated by the height of the wedge. **d**, Serial dilutions of five evolved populations of T7 and a control population passaged without selective pressure spotted on lawns of cells harbouring an EV or a CapRel^Ebc^ expression vector. **e**, Summary of the identified escape mutants of T7, all of which map to the gene encoding Gp0.4. **f**, Schematics showing the gene neighbourhoods of Gp0.4 in the indicated, closely related phages, as visualized with the CAGECAT clinker web server^45^. Homologues of Gp0.4 are highlighted in purple. **g**, Serial dilutions of the indicated phages spotted on lawns of cells harbouring an EV or a CapRel^Ebc^ expression vector. **h**, Growth of cells producing CapRel^Ebc^ or CapRel^Ebc^(Y153A) from its native promoter or an EV and Gp0.4 from an arabinose-inducible promoter (P_*ara*_) measured at the times indicated postaddition of glucose (for suppression) or arabinose (for induction). Mean and s.e.m. of three biological replicates are shown. *P = 0.008 (at 120 min between EV and CapRel^Ebc^, unpaired two-tailed *t*-test). aa, amino acid. Source data

**Extended Data Fig. 1:**
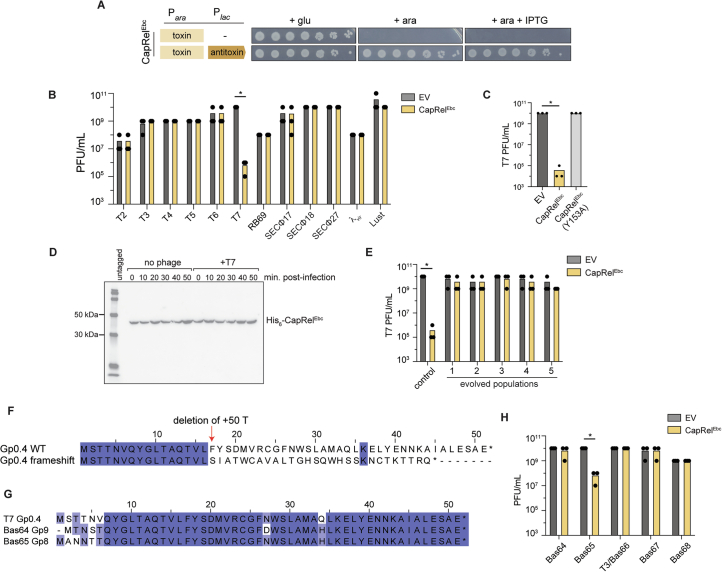
Activation of CapRel^Ebc^ by Gp0.4. (**a**) Cell viability assessed by serial dilutions for strains producing the N-terminal toxin domain of CapRel^Ebc^ alone or with the C-terminal antitoxin domain. (**b**) Summary of 3 independent replicates of phage spotting assay in Fig. 1b. Asterisk indicates p = 10^−18^ (unpaired two-tailed t-test). (**c**) Summary of 3 independent replicates of phage spotting assay in Fig. 1c. Asterisk indicates p = 10^−22^ (unpaired two-tailed t-test). (**d**) Immunoblot of His_6_-CapRel^Ebc^ following infection with T7 compared to an uninfected control. Representative of two independent experiments. (**e**) Summary of 3 independent replicates of phage spotting assay in Fig. 1d. Asterisk indicates p = 10^−18^ (unpaired two-tailed t-test). (**f**) Sequence alignment of wild-type Gp0.4 or the frameshifted variant identified in the escape phage mutants. (**g**) Multiple sequence alignment of Gp0.4 homologs from phages T7, Bas64 and Bas65. (**h**) Summary of 3 independent replicates of phage spotting assay in Fig. 1g. Asterisk indicates p = 10^−10^ (unpaired two-tailed t-test).

CapRel^Ebc^ is a predicted, fused toxin–antitoxin system identified from *E. chengduensis* that contains an N-terminal toxin domain and a C-terminal antitoxin domain (Fig. 1a). Production of the N-terminal domain alone was toxic to cells, and this toxicity was rescued by coproducing the C-terminal domain in trans (Extended Data Fig. 1a), confirming that CapRel^Ebc^ is a bona fide fused toxin–antitoxin system. To test if CapRel^Ebc^ defends against phages, we expressed it from its native promoter in *E. coli* MG1655 and tested for defence against a panel of diverse coliphages. CapRel^Ebc^ decreased the efficiency of plaquing (EOP) of phage T7 by 10^5^-fold, indicating it provides strong protection against this phage (Fig. 1b,c and Extended Data Fig. 1b). Defence by CapRel^Ebc^ depended on the predicted enzymatic activity of the toxin domain, as substituting the conserved tyrosine (Y153A) in the substrate-binding G-loop abolished defence^19^ (Fig. 1c and Extended Data Fig. 1c).

Because full-length CapRel^Ebc^ was not toxic in the absence of phage, and it was not proteolytically processed during phage infection (Extended Data Fig. 1d), we hypothesized that it is activated by a phage-encoded factor that relieves auto-inhibition by the antitoxin domain. To identify such a factor, we experimentally evolved T7 to overcome defence by CapRel^Ebc^ and obtained five populations that each fully escaped defence (Fig. 1d and Extended Data Fig. 1e). We isolated phage clones from each population and found that these clones all had mutations in gene *0.4*, which encodes Gp0.4, a small protein of 51 amino acids (Fig. 1e). Three clones had the same deletion of a single nucleotide in the coding sequence, leading to a frameshift and a premature stop codon (Extended Data Fig. 1f). The other clones each had a single nucleotide change that led to a single amino acid substitution, W28L or L35P (Fig. 1e). These results suggested that mutations in gene *0.4* allowed T7 to overcome protection by CapRel^Ebc^.

Gene *0.4* is among the earliest expressed genes during T7 infection and is adjacent to gene *0.3*, which encodes for Ocr, an inhibitor of restriction-modification systems that also activates the PARIS defence system^20–22^. Similar to Ocr, Gp0.4 is not found in all phages in Autographiviridae, the family that T7 belongs to, though homologues were present in phages Bas64 and Bas65, with each being 88% identical to Gp0.4 from T7 (Fig. 1f and Extended Data Fig. 1g). Consistent with their similarity to T7 Gp0.4, CapRel^Ebc^ defended against Bas64 and Bas65, reducing their EOP by 10- to 100-fold and leading to smaller plaques (Fig. 1g and Extended Data Fig. 1h). Other related phages—such as T3, Bas67 and Bas68—do not encode homologues of Gp0.4 and completely escaped CapRel^Ebc^ defence (Fig. 1f,g). Based on these observations and our T7 escape mutants, we hypothesized that Gp0.4 is an activator of CapRel^Ebc^, and its loss-of-function enables phage escape.

To test whether Gp0.4 is sufficient to activate CapRel^Ebc^, we coproduced Gp0.4 with CapRel^Ebc^ in the absence of phage infection. Consistent with previous work^23^, overproduction of Gp0.4 alone was moderately toxic to *E. coli* (Fig. 1h). However, coproducing Gp0.4 and CapRel^Ebc^ led to increased toxicity, as manifested by a further reduction in growth rate compared with cells producing Gp0.4 alone (Fig. 1h). This additional toxicity depended on the substrate-binding Y153 of CapRel^Ebc^, suggesting that the enzymatic activity of CapRel^Ebc^ toxin is required (Fig. 1h). Together, our results indicated that Gp0.4 can activate CapRel^Ebc^ without any other phage factors and that escape phages overcome defence by preventing activation.

### Activation of CapRel^Ebc^ by Gp0.4 depends on FtsZ

**Fig. 2:**
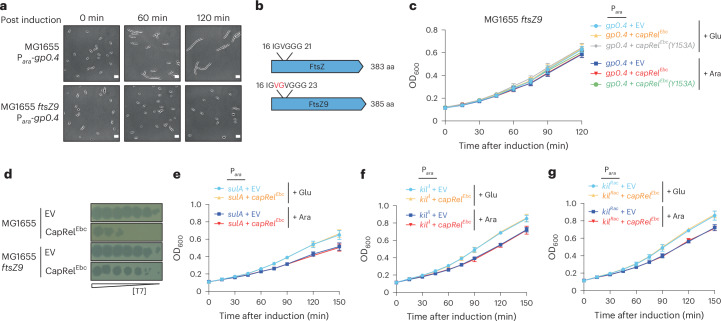
Activation of CapRel^Ebc^ by Gp0.4 depends on FtsZ. **a**, Phase-contrast microscopy images of cells producing Gp0.4 from an arabinose-inducible promoter (P_*ara*_) in wild-type *E. coli* MG1655 or the suppressor strain harbouring *ftsZ9* at the times indicated postaddition of arabinose. Scale bars, 5 µm. **b**, Schematics of FtsZ in wild-type *E. coli* cells or the suppressor strain (FtsZ9). The suppressor mutant FtsZ9 contains a duplication of residues valine–glycine at position 18 in FtsZ. **c**, Growth of cells harbouring the *ftsZ9* mutation in its genome and producing CapRel^Ebc^or CapRel^Ebc^(Y153A) from its native promoter and Gp0.4 from an arabinose-inducible promoter measured at the times indicated postaddition of glucose or arabinose. Mean and s.e.m. of three biological replicates are shown. **d**, Serial dilutions of the phage T7 spotted on lawns of cells harbouring an empty vector (EV) or a CapRel^Ebc^ expression vector in wild-type *E. coli* MG1655 or the suppressor strain harbouring *ftsZ9*. **e**, Growth of cells producing CapRel^Ebc^ from its native promoter or an EV and SulA from an arabinose-inducible promoter measured at the times indicated postaddition of glucose or arabinose. Mean and s.e.m. of three biological replicates are shown. **f**, As in **e** but for Kil peptide from coliphage λ (Kil^λ^). **g**, As in **e** but for Kil peptide from a *E. coli* cryptic prophage Rac (Kil^Rac^). Source data

**Extended Data Fig. 2:**
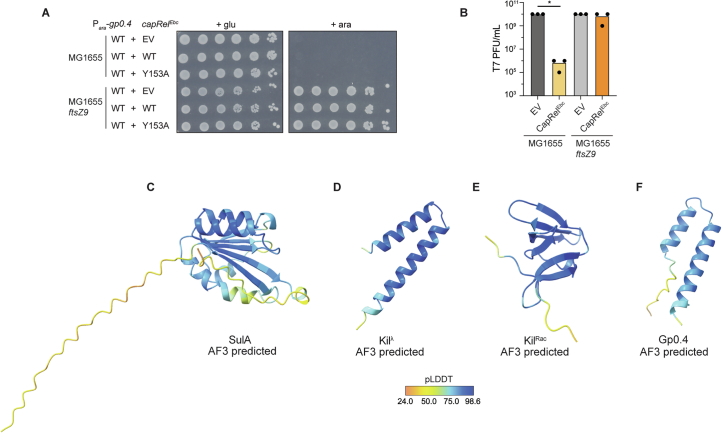
Gp0.4 does not activate CapRel^Ebc^ in *ftsZ9* suppressor strain. (**a**) Serial dilutions of wild-type *E. coli* or cells harboring *ftsZ9* suppressor mutation and producing CapRel^Ebc^ or CapRel^Ebc^ (Y153A) from its native promoter and Gp0.4 from an arabinose-inducible promoter spotted on media containing glucose or arabinose. (**b**) Summary of 3 independent replicates of phage spotting assay in Fig. 2d. Asterisk indicates p = 10^−18^ (unpaired two-tailed t-test). (**c–f**) AlphaFold3-predicted structures of SulA from *E. coli*, Kil from phage λ, or Kil from prophage Rac, or Gp0.4 from T7, colored by pLDDT.

Previous work has shown that Gp0.4 from T7 can disrupt bacterial cell division by inhibiting FtsZ, a tubulin homologue in bacteria that polymerizes into the Z-ring at mid-cell that is essential for cytokinesis^18,23^. Consistent with this prior work, we found that overproducing Gp0.4 caused wild-type *E. coli* cells to form elongated filaments, indicating an inhibition of cell division (Fig. 2a). We isolated a spontaneous suppressor mutant of *E. coli* that was resistant to Gp0.4 overproduction and found that this mutant contained a duplication of residues Val-18 and Gly-19 in FtsZ (Fig. 2b). This same FtsZ variant was previously identified (called *ftsZ9*) independently as resistant to the FtsZ inhibitor SulA, and shown to overcome other FtsZ inhibitors, such as the Kil peptide from phage λ (refs. ^24–26^). Overproduction of Gp0.4 was no longer toxic to cells harbouring *ftsZ9* (Fig. 2a), further supporting that Gp0.4 is a phage-encoded FtsZ inhibitor. Note that overproducing Gp0.4 on short timescales drives extensive cellular elongation (Fig. 2a) but only a modest decrease in cell growth (Fig. 1h), whereas on longer timescales, cells become extremely filamentous, cannot divide and, thus, do not form viable colonies (Extended Data Fig. 2a); similar patterns are seen with other cell division/FtsZ inhibitors^27–30^.

To test whether FtsZ is involved in CapRel^Ebc^ activation, we measured bacterial growth in the *ftsZ9* suppressor strain following coproduction of Gp0.4 and CapRel^Ebc^. Unlike in the wild-type strain, coproducing Gp0.4 alone or with CapRel^Ebc^ was no longer toxic to cells harbouring *ftsZ9* (Fig. 2c and Extended Data Fig. 2a). In addition, CapRel^Ebc^ mostly lost the ability to defend against T7 in the *ftsZ9* strain (Fig. 2d and Extended Data Fig. 2b). These results indicated that Gp0.4 cannot activate CapRel^Ebc^ in the FtsZ suppressor mutant strain and that wild-type FtsZ is required for CapRel^Ebc^ activation by Gp0.4.

Bacteria often encode protein inhibitors of FtsZ to regulate cell division, such as the *E. coli* protein SulA that is induced following DNA damage^25,31,32^. Some phages also produce FtsZ inhibitors, such as the Kil peptide from coliphage λ and cryptic prophage Rac, to halt cell division of their host bacteria^24,33,34^. These other FtsZ inhibitors are not homologues of Gp0.4 and are predicted to adopt different structures (Extended Data Fig. 2c–f). To test whether these other FtsZ inhibitors also activate CapRel^Ebc^, we coproduced each protein (SulA, Kil^λ^ or Kil^Rac^) with CapRel^Ebc^ and measured cell growth. As cell division inhibitors, each protein led to a growth defect in *E. coli* when individually produced (Fig. 2e–g). However, there was no additional toxicity in the presence of CapRel^Ebc^, as seen with Gp0.4 (Fig. 1h), suggesting that these other FtsZ inhibitors do not activate CapRel^Ebc^ (Fig. 2e–g). Together, our results demonstrated that the activation of CapRel^Ebc^ by Gp0.4 depends on the host cell division protein FtsZ but that other FtsZ inhibitors are not sufficient to activate CapRel^Ebc^.

### Analysis of FtsZ inhibition and CapRel^Ebc^ activation by Gp0.4

**Fig. 3:**
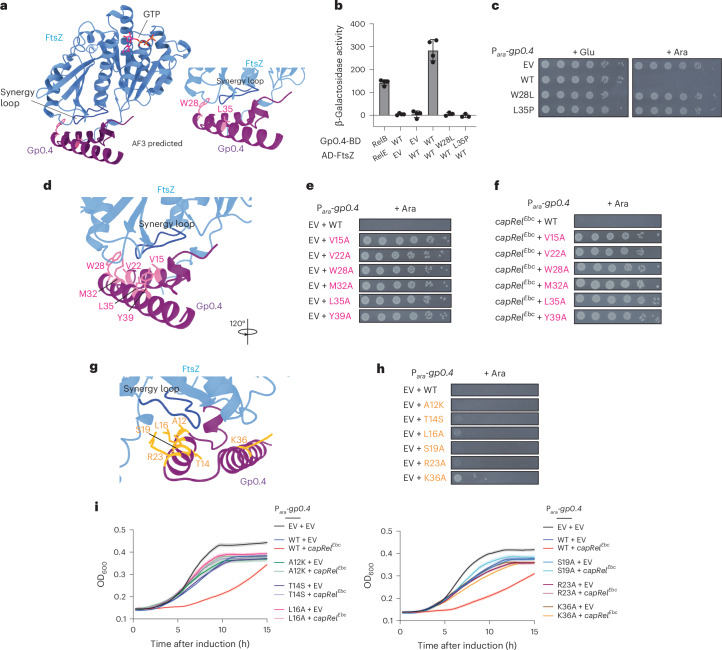
Analysis of FtsZ inhibition and CapRel^Ebc^ activation by Gp0.4. **a**, Left: AlphaFold3-predicted complex structure of Gp0.4 (purple) and FtsZ (blue) in the presence of GTP. The synergy loop of FtsZ is coloured in dark blue and labelled. Right: detailed interface of Gp0.4 and FtsZ, with the residues substituted in the escape phage mutants highlighted in pink. **b**, β-galactosidase activity of yeast two-hybrid strains producing wild type (WT) or the indicated variant of Gp0.4-BD and AD-FtsZ. RelB-BD and AD-RelE are used as a positive control for known interactions. Mean and s.d. of three to four biological replicates are shown. **c**, Cell viability assessed by serial dilutions of cells producing WT or the indicated variant of Gp0.4 from an arabinose-inducible promoter or an empty vector (EV) spotted on media containing glucose or arabinose. **d**, Detailed interface of AlphaFold3-predicted complex structure of Gp0.4 (purple) and FtsZ (blue). The synergy loop of FtsZ is coloured in dark blue and labelled. Residues in Gp0.4 substituted are highlighted in pink. **e**, Serial dilutions of cells producing an EV and WT or the indicated variant of Gp0.4 from an arabinose-inducible promoter (P_*ara*_) spotted on media containing arabinose. **f**, Serial dilutions of cells producing CapRel^Ebc^from its native promoter and WT or the indicated variant of Gp0.4 from an arabinose-inducible promoter spotted on media containing arabinose. **g**, As in **d** except the substituted residues in Gp0.4 are highlighted in orange. **h**, As in **e**. **i**, Growth of cells producing CapRel^Ebc^ from its native promoter or an EV and Gp0.4 (WT or the indicated variant) from an arabinose-inducible promoter measured at the times indicated postaddition of arabinose. The data shown are the average of seven to ten plate replicates with shaded areas indicating the s.d. Representative of two independent experiments.

**Extended Data Fig. 3:**
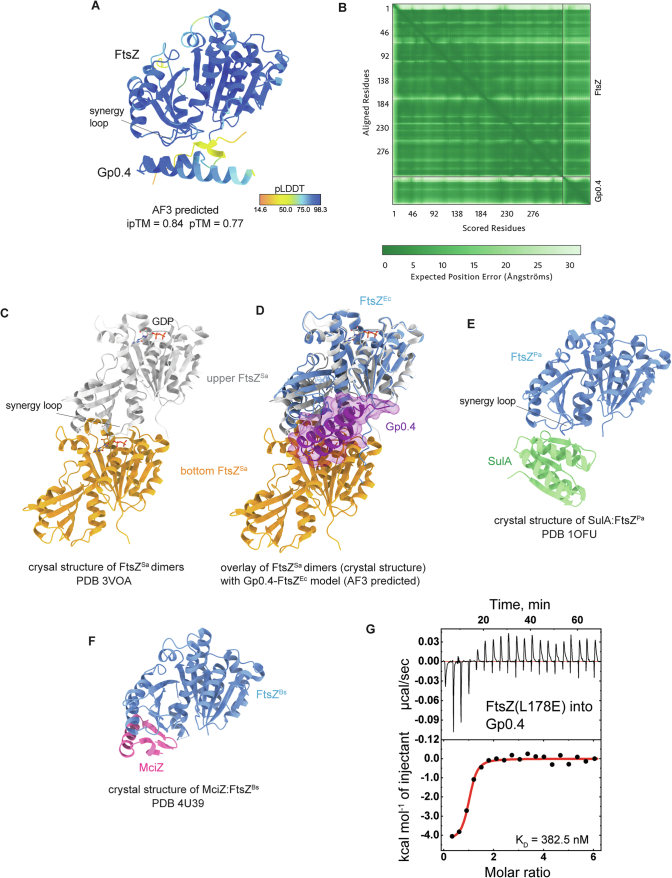
Analysis of FtsZ inhibition by Gp0.4 and other proteins. (**a,b**) AlphaFold3-predicted complex structure of Gp0.4 and FtsZ colored by pLDDT, with the metrics iPTM and pTM indicated (**a**), along with the PAE plot (**b**). (**c**) Crystal structure of FtsZ dimers from *Staphylococcus aureus* (PDB ID: 3VOA). The two subunits of FtsZ are colored in grey and orange respectively, with the synergy loop of the upper FtsZ in the interface indicated. (**d**) Superposition of the AlphaFold3-predicted Gp0.4-FtsZ^Ec^ complex structure onto the crystal structure of FtsZ^Sa^ dimers (PDB ID: 3VOA). FtsZ^Ec^ in the predicted complex is aligned to the upper subunit in the FtsZ^Sa^ dimers. (**e**) Crystal structure of SulA (green) in complex with FtsZ from *Pseudomonas aeruginosa* (FtsZ^Pa^ in blue, PDB ID: 1OFU). The synergy loop of FtsZ is highlighted. (**f**) Crystal structure of MciZ (pink) in complex with FtsZ from *Bacillus subtilis* (FtsZ^Bs^ in blue, PDB ID: 4U39). (**g**) Binding of Gp0.4 to the monomeric FtsZ(L178E) monitored by ITC, with binding affinity K_D_ noted.

Because the activation of CapRel^Ebc^ depends on both the phage protein Gp0.4 and the host protein FtsZ, we sought to analyse the interaction between Gp0.4 and FtsZ to understand how it impacts CapRel^Ebc^ activation. To examine the interaction interface between Gp0.4 and FtsZ, we used AlphaFold3 to predict a complex model^35^. Gp0.4, which is only 51 amino acids, was predicted to form two antiparallel α-helices and to interact with the C-terminal region of FtsZ, with a contact interface of ~1,000 Å^2^ (Fig. 3a and Extended Data Fig. 3a,b). The predicted binding interface involves the synergy loop of FtsZ, which contains the catalytic residues for GTP hydrolysis and is inserted into the N-terminal nucleotide-binding pocket of an adjacent FtsZ monomer upon FtsZ polymerization^36,37^ (Extended Data Fig. 3c). Through binding to the synergy loop-containing surface of FtsZ, Gp0.4 probably occludes binding of another FtsZ monomer, thereby preventing FtsZ polymerization (Extended Data Fig. 3d), similar to the FtsZ inhibitors SulA from *E. coli* and MciZ from *Bacillus* *subtilis*^32,38^ (Extended Data Fig. 3e,f).

To further validate the interaction of Gp0.4 and FtsZ, we used a yeast two-hybrid system with Gp0.4 fused to GAL4-BD and FtsZ fused to GAL4-AD and measured β-galactosidase activity. Gp0.4-BD or AD-FtsZ alone produced no detectable signal, whereas the two proteins together led to β-galactosidase activity comparable to that achieved when producing known binding partners RelB and RelE (Fig. 3b). In addition, isothermal titration calorimetry (ITC) indicated that purified Gp0.4 directly binds FtsZ(L178E), a monomeric variant of FtsZ^39^, with an affinity of 382 nM (Extended Data Fig. 3g). Together, our analyses indicate that Gp0.4 and FtsZ form a stable complex.

We had isolated escape mutants of T7 encoding Gp0.4 variants (W28L or L35P) that fully escape CapRel^Ebc^ defence (Fig. 1d,e). The residues W28 and L35 in Gp0.4 both map to the predicted binding interface formed with FtsZ (Fig. 3a) and probably both contribute to the interaction. The single substitutions W28L and L35P each abolished the toxicity of Gp0.4 when overproduced in *E. coli* (Fig. 3c), and these Gp0.4 variants no longer interacted with FtsZ in the yeast two-hybrid assay (Fig. 3b). These results indicated that the escape variants of Gp0.4 abolished the inhibition of FtsZ and prevent activation of CapRel^Ebc^ to allow phage escape.

**Extended Data Fig. 4:**
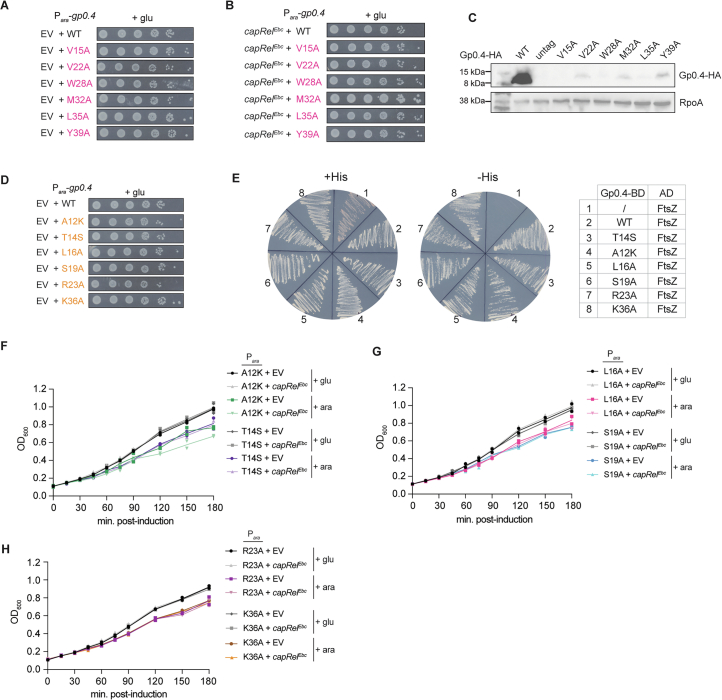
Mutational analysis of FtsZ inhibition and CapRel^Ebc^ activation by Gp0.4. (**a**) Serial dilutions of cells producing an empty vector and wild-type or the indicated variant of Gp0.4 from an arabinose-inducible promoter spotted on media containing glucose. (**b**) Serial dilutions of cells producing CapRel^Ebc^ from its native promoter and wild-type or the indicated variant of Gp0.4 from an arabinose-inducible promoter spotted on media containing glucose. (**c**) Immunoblot of Gp0.4-HA (wild-type or the indicated variant) in cells upon induction of Gp0.4 expression. Representative of three independent experiments. (**d**) Same as in (**a**). (**e**) Yeast two-hybrid strains producing Gp0.4-BD (wild-type or variant) and AD-FtsZ as indicated on the right streaked on plates containing (+His) or lacking (-His) histidine. (**f–h**) Growth of cells producing CapRel^Ebc^ from its native promoter or an empty vector and each Gp0.4 variant from an arabinose-inducible promoter measured at the times indicated post-addition of glucose or arabinose. 2 biological replicates are shown.

To further validate the predicted complex of Gp0.4 and FtsZ, we made single substitutions (V15A, V22A, W28A, M32A, L35A or Y39A) of additional solvent-exposed residues in Gp0.4 predicted to interact with FtsZ (Fig. 3d). Overproducing each Gp0.4 variant, unlike the wild type Gp0.4, was not toxic to *E. coli*, suggesting that they can no longer inhibit FtsZ (Fig. 3e and Extended Data Fig. 4a). Coproducing these Gp0.4 variants with CapRel^Ebc^ also did not affect cell growth, indicating that these variants abolish the activation of CapRel^Ebc^ (Fig. 3f and Extended Data Fig. 4b). These Gp0.4 variants accumulated to lower levels in cells compared with the wild-type Gp0.4, probably because interaction with FtsZ is important for its stability (Extended Data Fig. 4c). These mutational analyses, along with the phage escape mutants, supported our model that an interaction with FtsZ is critical for Gp0.4 to activate CapRel^Ebc^.

Next, we wanted to test whether FtsZ inhibition and CapRel^Ebc^ activation can be genetically separated, so we looked for residues in Gp0.4 that are predicted not to be involved in the interface with FtsZ but are solvent-exposed and potentially involved in binding CapRel^Ebc^. We made single substitutions in each such residue of Gp0.4: A12K, T14S, L16A, S19A, R23A or K36A (Fig. 3g). Overproducing each Gp0.4 variants was still toxic to *E. coli* (Fig. 3h and Extended Data Fig. 4d), and each maintained an interaction with FtsZ in the yeast two-hybrid assay (Extended Data Fig. 4e), suggesting they still bind and inhibit FtsZ. To test whether they activate CapRel^Ebc^, we coproduced each Gp0.4 variant with CapRel^Ebc^ or with an empty vector, and compared the rate of bacterial growth following Gp0.4 induction (Fig. 3i and Extended Data Fig. 4f–h). Unlike the wild-type Gp0.4, none of these variants had increased toxicity in the presence of CapRel^Ebc^, indicating that they were unable to activate CapRel^Ebc^ (Fig. 3i and Extended Data Fig. 4f–h). These results further indicated that an inhibition of cell division via FtsZ is not sufficient to activate CapRel^Ebc^ and that the residues A12, T14, L16, S19, R23 and K36 in Gp0.4 are important for CapRel^Ebc^ activation despite not being involved in FtsZ inhibition.

### FtsZ facilitates the interaction between CapRel^Ebc^ and Gp0.4

**Fig. 4:**
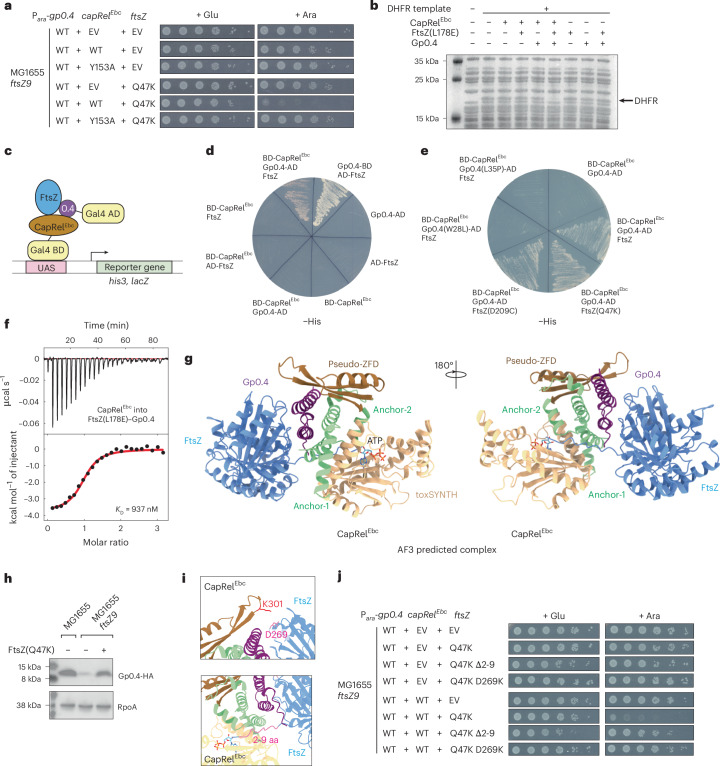
FtsZ facilitates interaction of CapRel^Ebc^ and Gp0.4. **a**, Serial dilutions of cells harbouring *ftsZ9* suppressor mutation and producing CapRel^Ebc^ or CapRel^Ebc^(Y153A) from its native promoter, Gp0.4 from an arabinose-inducible promoter (P_*ara*_) and FtsZ(Q47K) or an empty vector spotted on media containing glucose or arabinose. **b**, In vitro transcription–translation assays using DHFR production from a DNA template as readout. Purified CapRel^Ebc^, FtsZ(L178E) or Gp0.4 were added to the reactions. Representative of three biological replicates. **c**, A schematic of yeast two-hybrid experiment with a strain producing BD-tagged CapRel^Ebc^, AD-tagged Gp0.4 and untagged FtsZ, using *his3* and *lacZ* as reporter genes. **d**, Yeast two-hybrid strains producing a BD-tagged protein, an AD-tagged protein and an untagged protein as indicated streaked on plates lacking histidine (−His). **e**, As in **d**. **f**, Binding of CapRel^Ebc^ to FtsZ(L178E)-Gp0.4 as monitored by ITC. **g**, AlphaFold3-predicted complex structure of Gp0.4 (purple), FtsZ (blue) and CapRel^Ebc^ (coloured by domains) in the presence of ATP. The toxin domain (toxSYNTH), pseudo-ZFD and the connecting anchor helices (anchor-1 and anchor-2) of CapRel^Ebc^ are coloured accordingly. **h**, Immunoblot of Gp0.4-HA in wild-type *E. coli* MG1655 or the suppressor strain harbouring *ftsZ9*, with or without coproduction of FtsZ(Q47K). Image shown is a representative of three biological replicates. **i**, Details of the predicted interactions between CapRel^Ebc^ and FtsZ (blue). The predicted interaction residues CapRel^Ebc^(K301) and FtsZ(D269) are highlighted (top), and the most N-terminal 2-9 residues of FtsZ are coloured in pink (bottom). **j**, Serial dilutions of cells harbouring *ftsZ9* suppressor mutation and producing CapRel^Ebc^ from its native promoter, Gp0.4 from an arabinose-inducible promoter and the indicated variant of FtsZ or an empty vector spotted on media containing glucose or arabinose.

**Extended Data Fig. 5:**
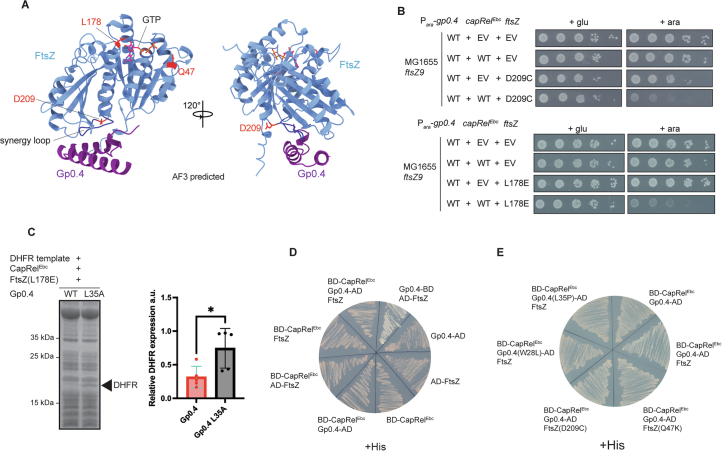
Gp0.4 activates CapRel^Ebc^ likely through a ternary complex with FtsZ. (**a**) AlphaFold3-predicted complex structure of Gp0.4 (purple) and FtsZ (blue) in the presence of GTP. The synergy loop of FtsZ is colored in dark blue and labeled. The residues Q47, L178 and D209 of FtsZ are highlighted in red. (**b**) Serial dilutions of cells harboring *ftsZ9* suppressor mutation and producing CapRel^Ebc^ from its native promoter, Gp0.4 from an arabinose-inducible promoter, and the indicated FtsZ variant or an empty vector spotted on media containing glucose or arabinose. (**c**) In vitro transcription-translation assays using DHFR production from a DNA template as readout. Purified CapRel^Ebc^, FtsZ(L178E), or Gp0.4 (wild-type or L35A variant) were added to the reactions. *Left*, representative image. *Right*, quantification of the relative intensity of the bands. Asterisk indicates p = 0.03 (Ordinary one-way ANOVA). Mean and SD of five biological replicates are shown. (**d,e**) Yeast two-hybrid strains producing a BD-tagged protein, an AD-tagged protein and an untagged protein as indicated streaked on plates containing histidine (+His).

Given that an interaction with FtsZ is probably necessary, though not sufficient, for Gp0.4 to activate CapRel^Ebc^, we hypothesized that the activation of CapRel^Ebc^ may involve a tripartite complex involving both Gp0.4 and FtsZ. Thus, we wondered whether Gp0.4 bound to monomeric FtsZ binds and activates CapRel^Ebc^. To test this hypothesis in vivo, we examined three previously characterized variants of FtsZ harbouring a substitution in the GTP-binding surface (Q47K or L178E) or the synergy loop-containing surface (D209C)^39,40^ (Extended Data Fig. 5a). Each substitution does not support cell division and probably disrupts FtsZ polymerization^39,40^. To test if these presumed monomeric mutants of FtsZ, along with Gp0.4, can activate CapRel^Ebc^, we coproduced each FtsZ variant with wild-type Gp0.4 and CapRel^Ebc^ in *E. coli* harbouring *ftsZ9* (the suppressor mutant) in its genome. Gp0.4 did not activate CapRel^Ebc^ on its own in this strain; however, coproduction of FtsZ(Q47K) with Gp0.4 led to a growth defect that was dependent on the presence of CapRel^Ebc^ and its catalytic activity (Fig. 4a). Similar results were observed with the D209C or L178E substitutions of FtsZ (Extended Data Fig. 5b). Next, we tested whether Gp0.4 and FtsZ can activate CapRel^Ebc^ in vitro using a reconstituted transcription–translation system. Incubating purified Gp0.4 and FtsZ(L178E) with CapRel^Ebc^ inhibited the synthesis of a model protein DHFR, whereas neither protein alone led to translation inhibition (Fig. 4b). No inhibition of DHFR synthesis was observed with the L35A variant of Gp0.4 (Extended Data Fig. 5c). These findings strongly suggest that monomeric FtsZ allows Gp0.4 to activate CapRel^Ebc^.

To test whether FtsZ, Gp0.4 and CapRel^Ebc^ form a tripartite complex that leads to CapRel^Ebc^ activation, we again used the yeast two-hybrid system to examine interactions. We first tested interactions between CapRel^Ebc^ and Gp0.4, or between CapRel^Ebc^ and FtsZ, but observed no detectable growth on media lacking histidine, suggesting either Gp0.4 or FtsZ alone was not sufficient to interact with CapRel^Ebc^ (Fig. 4c,d and Extended Data Fig. 5d). We then produced untagged FtsZ along with BD-CapRel^Ebc^ and Gp0.4-AD in yeast and saw growth on media lacking histidine, indicating a potential interaction between CapRel^Ebc^ and Gp0.4 in the presence of FtsZ (Fig. 4c,d and Extended Data Fig. 5d). Each of the FtsZ monomeric variants (Q47K or D209C) also supported this interaction (Fig. 4e and Extended Data Fig. 5e). The escape variants of Gp0.4 (W28L or L35P) were unable to interact with CapRel^Ebc^ in the presence of FtsZ (Fig. 4e and Extended Data Fig. 5e). Together, these results indicated that FtsZ facilitates an interaction between CapRel^Ebc^ and Gp0.4, possibly through formation of a ternary complex.

### Gp0.4, FtsZ, and CapRel^Ebc^ form a ternary complex

**Extended Data Fig. 6:**
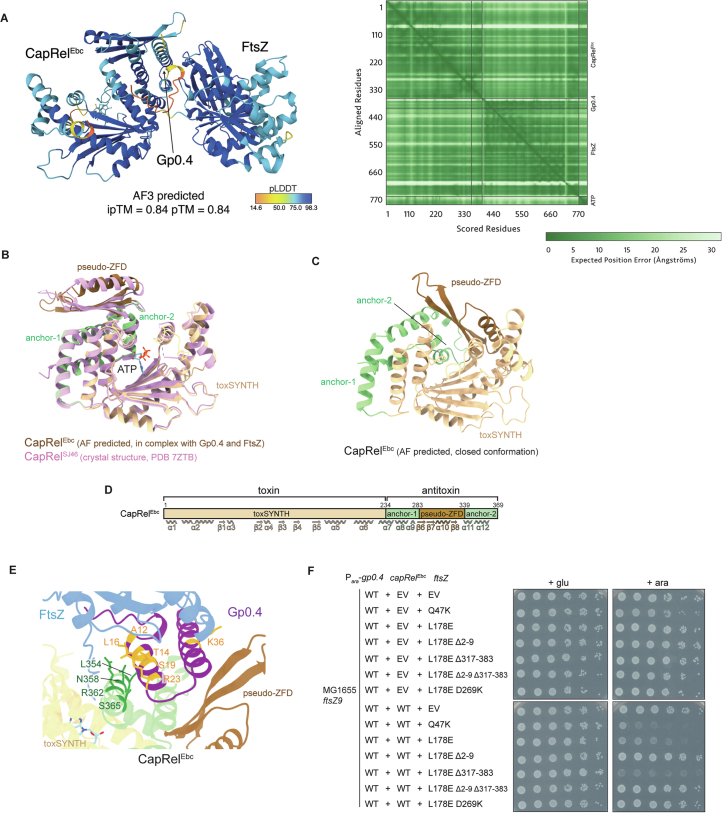
Model and validation of a CapRel^Ebc^-Gp0.4-FtsZ complex. (**a**) AlphaFold3-predicted complex structure of CapRel^Ebc^, Gp0.4, and FtsZ colored by pLDDT, with the metrics iPTM and pTM indicated (left), along with the PAE plot (right). (**b**) Superposition of CapRel^Ebc^ (colored by domains) in the AlphaFold3-predicted Gp0.4-FtsZ-CapRel^Ebc^ complex onto the open, active structure of CapRel^SJ46^ (PDB ID: 7ZTB, colored in pink). (**c**) AlphaFold3-predicted complex structure of CapRel^Ebc^ colored by domains. (**d**) Domain organization and the predicted secondary structural elements of CapRel^Ebc^. (**e**) Detailed interfaces in AlphaFold3-predicted complex structure of Gp0.4 (purple), FtsZ (blue), and CapRel^Ebc^ (colored by domains). The substituted residues in Gp0.4 are colored in orange, and the predicted interaction residues in the anchor helix of CapRel^Ebc^ are colored in green. (**f**) Serial dilutions of cells harboring *ftsZ9* suppressor mutation and producing CapRel^Ebc^ from its native promoter, Gp0.4 from an arabinose-inducible promoter, and the indicated FtsZ variant or an empty vector spotted on media containing glucose or arabinose.

Using ITC we confirmed that CapRel^Ebc^ directly interacts with the Gp0.4–FtsZ(L178E) complex with an affinity of 937 nM and a 1:1:1 molar ratio (Fig. 4f). To further explore the interactions between FtsZ, Gp0.4 and CapRel^Ebc^, we used AlphaFold3 to predict a ternary complex structure^35^ (Fig. 4g and Extended Data Fig. 6a). In this predicted complex, CapRel^Ebc^ adopts an open conformation with ATP bound to the pyrophosphate donor site, which is comparable to the conformation seen in the crystal structure of open, active CapRel^SJ46^ (ref. ^12^) (Extended Data Fig. 6b). Notably, this conformation is distinct from the predicted structure of CapRel^Ebc^ on its own, where the C-terminal antitoxin folds back and blocks the ATP-binding site of the N-terminal toxin domain (Extended Data Fig. 6c,d). Gp0.4 is predicted to interact with both FtsZ and CapRel^Ebc^, with a contact interface of over 1,000 Å^2^ with each partner (Fig. 4g). In this ternary complex, the interaction between Gp0.4 and FtsZ is comparable to the predicted FtsZ-Gp0.4 complex in the absence of CapRel^Ebc^ (Fig. 3a). We hypothesized that binding to FtsZ stabilizes the α-helical structure of Gp0.4, facilitating its interaction with CapRel^Ebc^. Consistent with this hypothesis, Gp0.4 accumulated to lower levels in *E. coli* cells harbouring *ftsZ*9 (the suppressor strain) compared with that in wild-type cells (Fig. 4h). Coproduction with a monomeric FtsZ(Q47K) rescued the expression level of Gp0.4 (Fig. 4h), indicating that FtsZ stabilizes Gp0.4.

In the predicted ternary complex, the α-helices of Gp0.4 interact with the C-terminal antitoxin domain of CapRel^Ebc^, including both the zinc-finger-like domain (pseudo-ZFD) and the anchor helices that flank the pseudo-ZFD (Fig. 4g and Extended Data Fig. 6d). The substitutions identified above (Fig. 3h,i) that specifically disrupted the interaction between Gp0.4 and CapRel^Ebc^ were in residues (A12, T14, L16, S19 and R23) that map to the predicted contact interface between Gp0.4 and the most C-terminal anchor helix of CapRel^Ebc^ (Extended Data Fig. 6e). The C-terminal antitoxin domain of the homologue CapRel^SJ46^ directly binds multiple phage-encoded activators, serving as a phage infection sensor that regulates the auto-inhibition of the N-terminal toxin domain and determines the specificity of phage defence^12,17^. Notably, CapRel^SJ46^ and CapRel^Ebc^ have 70% identity overall at the amino acid level, but their C-terminal antitoxin domains are substantially more divergent (only 47% identical) (Extended Data Fig. 7). This region is also the least conserved across diverse CapRel homologues^12^. Unlike CapRel^SJ46^ that senses the major capsid protein or the small protein Gp54 of some phages, CapRel^Ebc^ specifically senses Gp0.4 together with FtsZ. By binding to the C-terminal anchor helices and the pseudo-ZFD, Gp0.4 probably stabilizes the open conformation of CapRel^Ebc^, such that the antitoxin domain cannot neutralize the N-terminal toxin, leading to CapRel^Ebc^ activation.

The predicted ternary complex also included interactions between FtsZ and CapRel^Ebc^, which may further stabilize the complex and promote CapRel^Ebc^ activation (Fig. 4g). AlphaFold3 predicted a salt bridge between K301 in the pseudo-ZFD of CapRel^Ebc^ and D269 of FtsZ, as well as a potential interaction between N-terminal residues 2-9 of FtsZ and CapRel^Ebc^ (Fig. 4i). Consistent with these predictions, the substitution D269K or a deletion of residues 2–9 in FtsZ largely abolished activation of CapRel^Ebc^ when introduced to the monomeric FtsZ mutant (Q47K or L178E) and coproduced with Gp0.4 (Fig. 4j and Extended Data Fig. 6f). Deletion of the disordered C-terminal linker of FtsZ had no effect on CapRel^Ebc^ activation (Extended Data Fig. 6f).

**Fig. 5:**
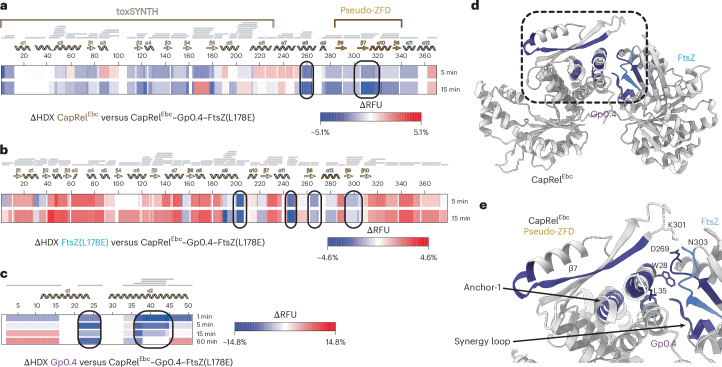
CapRel^Ebc^, Gp0.4 and FtsZ form a ternary complex. **a–c**, ΔHDX between CapRel^Ebc^ and CapRel^Ebc^–Gp0.4–FtsZ(L178E) (**a**), FtsZ(L178E) and CapRel^Ebc^–Gp0.4–FtsZ(L178E) (**b**) and Gp0.4 and CapRel^Ebc^–Gp0.4–FtsZ(L178E) (**c**), each plotted as a heat map. RFU, relative fractional uptake. **d**, Structure of the CapRel^Ebc^–Gp0.4–FtsZ complex predicted by AlphaFold3, with residues showing the largest decreased deuterium exchange coloured. **e**, Details of the complex interface highlighting the substitutions observed in the escape clones and the direct contacts between CapRel^Ebc^ and FtsZ.

**Fig. 6:**
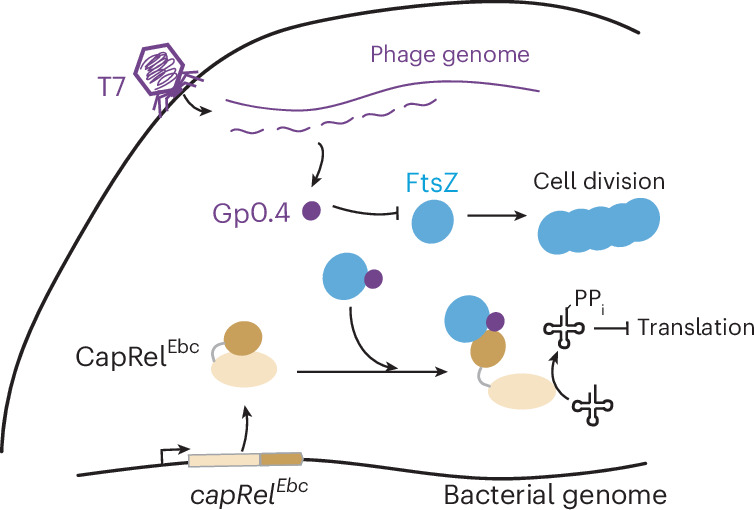
Model for the activation of CapRel^Ebc^ during T7 infection. A small phage protein Gp0.4 blocks polymerization of the essential cell division protein FtsZ; together, Gp0.4 and FtsZ bind to and activate CapRel^Ebc^, which can pyrophosphorylate tRNAs (designated by PP_i_ at the 3’ end of a tRNA) to block translation and prevent phage replication.

To further validate the predicted ternary complex CapRel^Ebc^–Gp0.4–FtsZ, we used hydrogen–deuterium exchange mass spectrometry (HDX-MS). We compared the deuterium exchange profile of CapRel^Ebc^–Gp0.4–FtsZ(L178E) with those of the individual proteins, using changes in deuterium uptake as a proxy for changes in solvent accessibility (Fig. 5a–c). The change (ΔHDX) for CapRel^Ebc^ confirmed interaction via the antitoxin domain (Fig. 5a). The global protection pattern was similar to that observed in the recognition of Gp54 and the major capsid protein Gp57 by CapRel^SJ46^ (refs. ^12,17^), with anchor-1 (residues 254–264) and β7 of the pseudo-ZFD (residues 303–312) identified as the most strongly protected regions (Fig. 5d,e). The change in deuterium uptake in FtsZ(L178E) upon complex formation confirmed that interactions with Gp0.4 are primarily mediated by the synergy loop (residues 199–206), along with residues 262–270 from β8-α12 and 292–303 from β9–β10 (Fig. 5b,d,e). For Gp0.4, most of the protein was protected from deuterium exchange following complex formation, particularly residues 21–28 in α1 and 35–46 in α2, which includes the residues W28 and L35 substituted in the escape variants (Fig. 5c–e). Together, our results indicate that Gp0.4, along with the host cell division protein FtsZ, activates CapRel^Ebc^ through the formation of a ternary complex (Fig. 6).

## Discussion

Here, we identified a mechanism of antiphage defence activation that involves both a phage and a host-encoded factor. During T7 infection, Gp0.4 binds to FtsZ to block its polymerization and inhibit bacterial cell division. Together, Gp0.4 and FtsZ activate CapRel^Ebc^, probably through the formation of a tripartite complex that relieves auto-inhibition of the CapRel^Ebc^ toxin domain by its antitoxin domain. The inhibition of cell division by targeting FtsZ appears to be a common activity of diverse phages, including the Kil peptide from λ and DicB in the lambdoid prophage Qin^33,41^, though these inhibitors share no similarity. Notably, T7 phages lacking *0.4* have a competitive disadvantage against wild-type phages, suggesting that the inhibition of cell division is beneficial to phages, but the basis of this benefit is unclear^23^.

Most defence systems in bacteria must be activated during phage infection. Some systems exclusively sense a phage protein through direct binding, akin to PAMP-based sensing in eukaryotic innate immunity^7,8^. In eukaryotes, pathogen detection can also involve ETI in which innate immune proteins sense pathogen-associated activities rather than exclusively through binding pathogen structures^9,11^. CapRel^Ebc^ shares features of both PAMP and ETI-based strategies, binding to the T7 phage protein Gp0.4 but only once this phage-encoded effector has bound to monomeric FtsZ to inhibit cell division. Although akin to ETI in some ways, CapRel^Ebc^ does not solely sense the consequences of cell division inhibition as other FtsZ inhibitors did not trigger CapRel^Ebc^ activity. Instead, because CapRel^Ebc^ binds Gp0.4 in complex with the FtsZ monomer that it has sequestered, the activation mechanism shares elements of PAMP recognition and ETI.

Finally, our work raises two important issues related to the identification of phage activators of bacterial defence systems. First, despite being closely related, CapRel^Ebc^ and CapRel^SJ46^ recognize completely unrelated proteins. This observation highlights the notion that homologues cannot be assumed to have identical functions, a reflection of how fast viral and immunity proteins evolve. Second, we note that the triggers for antiphage defence systems are often identified by finding phage escape mutants or by showing that the expression of a single phage gene is sufficient to activate a defence system^42^. Such studies will intrinsically miss host factors that may also be critical to activation. Our work underscores the notion that in some cases it may in fact be phage proteins in complex with host targets that comprise the activation signal.

## Methods

### Strains and growth conditions

All bacterial and phage strains used in this study are listed in Supplementary Table 1. *E. coli* strains were routinely grown at 37 °C in Luria broth (LB) medium for cloning and maintenance. Phages were propagated by infecting a culture of *E. coli* MG1655 at an optical density at 600 nm (OD_600_) of ~0.1–0.2 with a multiplicity of infection (MOI) of 0.1. Cleared cultures were pelleted by centrifugation to remove residual bacteria and filtered through a 0.2-μm filter. Chloroform was then added to phage lysates to prevent bacterial growth. Antibiotics were used at the following concentrations (liquid; plates): carbenicillin (50 μg ml^−1^; 100 μg ml^−1^), chloramphenicol (20 μg ml^−1^; 30 μg ml^−1^).

### Plasmid construction

All plasmids are listed in Supplementary Table 2. All primers are listed in Supplementary Table 3.

pBAD33 constructs: wild type or mutant variants of *gp0.4* were PCR-amplified from the corresponding wild-type T7 or escaping phage clones using primers TZ-3 and TZ-4 and inserted into pBAD33 linearized with TZ-1 and TZ-2 using Gibson assembly. Mutations that produce the single amino acid substitutions were generated by site-directed mutagenesis. *kil*^*λ*^ was PCR-amplified from phage λ using primers TZ-7 and TZ-8, *kil*^*Rac*^ was PCR-amplified from *E. coli* MG1655 using primers TZ-5 and TZ-6, *sulA* was PCR-amplified from *E. coli* MG1655 using primers TZ-9 and TZ-10, and each was inserted into pBAD33 linearized with TZ-1 and TZ-2 using Gibson assembly. Primers TZ-11 and TZ-12 were used to add a strong RBS for pBAD33-sulA. *capRel*^*Ebc*^ (1–270) was PCR-amplified from pBR322-capRel^Ebc^ using primers TZ-13 and TZ-14 and inserted into linearized pBAD33 using Gibson assembly.

pEXT20 and pBR322 constructs: *capRel*^*Ebc*^ (271–369) was PCR-amplified from pBR322-capRel^Ebc^ using primers TZ-17 and TZ-18 and inserted into pEXT20 linearized with primers TZ-15 and TZ-16 using Gibson assembly. To add a N-terminal His_6_-tag, primers TZ-19 and TZ-20 were used to PCR-amplify pBR322-capRel^Ebc^ followed by Gibson assembly.

pKVS45 constructs: *ftsZ* was PCR-amplified from *E. coli* MG1655 using primers TZ-21 and TZ-22 and inserted into pKVS45 linearized with primers TZ-23 and TZ-24 using Gibson assembly. Mutations that produce the single amino acid substitutions or the truncated proteins were generated by site-directed mutagenesis.

Yeast two-hybrid constructs: GAL4 DNA binding domain was PCR-amplified from pGBDU-BD-EV using primer TZ-25 and TZ-26, and inserted into pBAD33-gp0.4 linearized with TZ-27 and TZ-28 to construct pBAD33-gp0.4-BD (with a (GGGGS)_2_ linker in between) to confirm the tag does not disrupt Gp0.4 function. *gp0.4-BD* was then PCR-amplified from pBAD33-gp0.4-BD using primers TZ-25 and TZ-29 and inserted into pGBDU-BD-EV linearized with primers TZ-30 and TZ-31. pGBDU-BD-capRel^Ebc^ was constructed similarly with a (GGGGS)_2_ linker in between *BD* and *capRel*^*Ebc*^. *ftsZ* was PCR-amplified using primers TZ-32 and TZ-33 from pKVS45-ftsZ, and inserted into pGAD-AD-EV linearized with primers TZ-34 and TZ-35. To construct pGAD-gp0.4-AD (with a (GGGGS)_2_ linker in between), we first constructed pBAD33-gp0.4-AD using primers TZ-36 and TZ-37 to amplify GAL4 activation domain and TZ-38 and TZ-39 to linearize pBAD33-gp0.4-BD. Then, *gp0.4-AD* was PCR-amplified using primers TZ-40 and TZ-41, and inserted into pGAD-AD-ftsZ vector linearized with TZ-35 and TZ-42. To construct pGAD-ftsZ, primers TZ-33 and TZ-43 were used to PCR-amplified *ftsZ*, which was inserted into pGAD-AD-ftsZ linearized with primers TZ-35 and TZ-42. AD-ftsZ with the ADH-1 promoter was PCR-amplified from pGAD-ftsZ with primers TZ-44 and TZ-45 and inserted into pGAD-gp0.4-AD linearized with primers TZ-46 and TZ-47.

### Strain construction

For bacterial strains, corresponding plasmids were introduced into *E. coli* MG1655 or BW27783 by TSS transformation or electroporation.

For yeast strains, corresponding plasmids were transformed into yeast *Saccharomyces cerevisiae* strain PJ69-4A by lithium acetate/ssDNA/PEG method. A total of 10 ml of overnight culture were pelleted at 3,000*g* for 3 min and resuspended in 50 μl TE + 0.1 M lithium acetate. A total of 1 μg of each plasmid was mixed with 240 μl of 50% PEG 3350, 36 μl of 1 M lithium acetate, 10 μl of water and 5 μl of 10 μg μl^−1^ salmon sperm single-stranded DNA (ssDNA). The transformation mix was added to cells and incubated at 30 °C for 30 min, followed by an incubation at 42 °C for 15 min. A total of 500 μl of TE + 1.2 M sorbitol was then added to each transformation, mixed and centrifuged at 3,000*g* for 3 min. The supernatant was removed, and pellet was resuspended in 500 μl of TE + 1.2 M sorbitol. A total of 100 μl was plated onto selected plates and incubated at 30 °C for 3 days.

### Phage spotting assays and EOP measurements

Phage spotting assays were conducted similarly to that described previously^12^. For phage spotting assays, 80 μl of a bacterial strain of interest was mixed with 4 ml LB + 0.5% agar and spread on an LB + 1.2% agar + antibiotic plate. Phage stocks were then serially diluted in 1× FM buffer (20 mM Tris–HCl pH 7.4, 100 mM NaCl, 10 mM MgSO_4_), and 2 μl of each dilution was spotted on the bacterial lawn. Plates were then incubated at 25 °C overnight before imaging. EOP was calculated by comparing the ability of the phage to form plaques on an experimental strain relative to the control strain.

### Isolation of phage escape mutants to infect CapRel^Ebc^

T7 phage was evolved to overcome CapRel^Ebc^ defence using an experimental evolution protocol as described previously^43^. In brief, five independent populations were evolved in a 96-well plate containing a sensitive host *E. coli* MG1655 pBR322-EV and a resistant host *E. coli* MG1655 pBR322-capRel^*Ebc*^. One control population was evolved with only the sensitive host. Overnight bacterial cultures were back-diluted to OD_600_of 0.1 in LB and 100 μl was seeded into each well. Cells were infected with 10-fold serial dilutions of T7 phage with MOI from 0.1 to 10^−7^. Plates were sealed with breathable plate seals and incubated at 25 °C for 7 h in a plate shaker at 1,000 rpm. Cleared wells from each population were pooled, pelleted at 4,000*g* for 20 min to remove bacteria, and the supernatant lysates were transferred to a 96 deep-well block with 40 µl chloroform added to prevent bacterial growth. Lysates were spotted onto both sensitive and resistant hosts to check the defence phenotype. Two rounds of evolution were performed. Evolved clones from each evolved population were isolated by plating to single plaques on lawns of resistant host, and control clones from the control population were isolated on a lawn of the sensitive host. One clone from each population were propagated using the corresponding host and sequenced as described below.

### Phage DNA extraction and Illumina sequencing

Phage DNA extraction and sequencing were conducted as described previously^12^. To extract phage DNA, high titre phage lysates were treated with DNase I (0.001 U µl^−1^) and RNase A (0.05 mg ml^−1^) at 37 °C for 30 min. A total of 10 mM EDTA was used to inactivate the nucleases. Lysates were then incubated with proteinase K at 50 °C for 30 min to disrupt capsids and release phage DNA. Phage DNA was isolated by ethanol precipitation. In brief, sodium acetate pH 5.2 was added to 300 mM followed by 100% ethanol to a final volume fraction of 70%. Samples were incubated at −80 °C overnight, pelleted at 21,000*g* for 20 min and supernatant removed. Pellets were washed with 100 µl isopropanol and 200 µl 70% (v/v) ethanol and then aired dried at room temperature and resuspended in 25 µl 1× TE buffer (10 mM Tris–HCl, 0.1 mM EDTA, pH 8).

To prepare Illumina sequencing libraries, 100–200 ng of genomic DNA was sheared in a Diagenode Bioruptor 300 sonicator water bath for 15× 30-s cycles at maximum intensity. Sheared genomic DNA was purified using AmpureXP beads, followed by end repair, 3′ adenylation, and adaptor ligation. Barcodes were added to both 5′ and 3′ ends by PCR with primers that anneal to the Illumina adaptors. The libraries were cleaned by AmpureXP beads using a double cut to elute fragment sizes matching the read-lengths of the sequencing run. Libraries were sequenced on an Illumina MiSeq at the MIT BioMicro Center. Illumina reads were assembled to the reference genomes using Geneious Prime 2022.0.2.

### Toxicity assays on solid media

Bacterial toxicity assays were conducted similarly to that described previously^12^.

For producing the CapRel^Ebc^ N- and C-terminal domains, single colonies of *E. coli* MG1655 habouring pBAD33-capRel^Ebc^ (1–270) and pEXT20-capRel^Ebc^ (271–369) or the corresponding empty vectors were grown for 6 h at 37 °C in LB–glucose to saturation. A total of 200 μl of each saturated culture was then pelleted by centrifugation at 4,000*g* for 10 min, washed once in 1× phosphate-buffered saline (PBS) and resuspended in 400 μl 1× PBS. Cultures were then serially diluted tenfold in 1× PBS and spotted on M9L plates (M9 medium supplemented with 5% LB (v/v)) further supplemented with 0.4% glucose, 0.2% arabinose or 0.2% arabinose and 100 μM IPTG. Plates were then incubated at 37 °C overnight before imaging.

For coproducing CapRel^Ebc^ and Gp0.4, *E. coli* MG1655 or MG1655 *ftsZ9* harbouring pBR322-capRel^Ebc^ and pBAD33-gp0.4 (wild type or the corresponding variants) were grown to saturation and processed as above. Cultures were plated onto 0.4% glucose and 0.2% arabinose and incubated at 37 °C overnight.

For producing Gp0.4 (wild type or mutant variant), *E. coli* BW27783 harbouring pBAD33-gp0.4 (wild type or the corresponding variants) were grown to saturation and processed as above. Cultures were plated onto 0.4% glucose and 0.2% arabinose and incubated at 37 °C overnight.

For coproducing CapRel^Ebc^, Gp0.4 and FtsZ, *E. coli* MG1655 *ftsZ9* harbouring pBR322-capRel^Ebc^, pBAD33-gp0.4 and pKVS45-ftsZ variant were grown to saturation and processed as above. Cultures were plated onto 0.4% glucose and 0.2% arabinose and incubated at 37 °C overnight.

### Growth curves in liquid culture

For coproducing CapRel^Ebc^ and FtsZ inhibitors, single colonies of *E. coli* MG1655 containing pBR322-EV or pBR322-capRel^Ebc^ and pBAD33-sulA or *kil*^*λ*^ or *kil*^*Rac*^ were grown overnight in M9-glucose. Overnight cultures were back-diluted to OD_600_ of ~0.05 in 25 ml of M9 (no glucose) and grown to OD_600_ of ~0.3 at 37 °C. Cells were back-diluted to OD_600_ of 0.1 in M9 (no glucose) and either induced with 0.2% arabinose or suppressed with 0.4% glucose at the beginning of the experiment. Cultures were continuously shaking at 200 rpm at 37 °C, and the OD_600_ was measured by spectrometer at the indicated time points.

For coproducing CapRel^Ebc^ and Gp0.4, single colonies of *E. coli* MG1655 containing pBR322-EV or pBR322-capRel^Ebc^ and pBAD33-gp0.4 were grown overnight in M9-glucose. Overnight cultures were back-diluted to OD_600_ of ~0.05 in 25 ml of M9-glucose and grown to OD_600_ of ~0.3 at 37 °C. Cells were pelleted at 4,000*g* for 5 min at 4 °C and washed once with M9 (no glucose) and then back-diluted to OD_600_ of 0.1 in 25 ml M9 (no glucose) and recovered for 45 min at 37 °C. At the beginning of the experiment, cells were induced with 0.2% arabinose or suppressed with 0.4% glucose. OD_600_ was measured at the indicated time points.

### Western blotting

To blot for CapRel^Ebc^ during phage infection, single colonies of *E. coli* MG1655 pBR322-His_6_-capRel^Ebc^ under its native promoter were grown overnight in LB. Overnight cultures were back-diluted to OD_600_ of 0.05 in 25 ml fresh LB and grown to OD_600_ of 0.2 at 25 °C. Cells were infected with phage T7 at an MOI of 5 and shaking at 200 rpm at 25 °C during the experiment. At each indicated time point (0, 10, 20, 30, 40 and 50 min), OD_600_ was measured and 2 ml of cells were pelleted at 21,000*g* for 2 min at 4 °C. Supernatant was removed and pellets were flash-frozen in liquid nitrogen. Pellets were thawed and resuspended in 1× Laemmli sample buffer (Bio-Rad) supplemented with 2-mercaptoethanol with OD_600_ normalized. Samples were then boiled at 95 °C and analysed by 12% SDS–PAGE and transferred to a 0.45-μm PVDF membrane. Anti-His_6_ antibody (Invitrogen, catalogue no. MA1-21315) was used at a final concentration of 1:1,000, and SuperSignal West Femto Maximum Sensitivity Substrate (ThermoFisher) was used to develop the blots. Blots were imaged by a ChemiDoc Imaging system (Bio-Rad).

To blot for Gp0.4 variants, single colonies of *E. coli* MG1655 pBAD33-gp0.4-HA (wild type or the corresponding variants) were grown overnight in M9-glucose at 37 °C. Cultures were back-diluted 1:100 in fresh M9 and grown to OD_600_ of ~0.3 at 37 °C. Cells were induced with 0.2% arabinose for 30 min, OD_600_ was measured, and 4 ml of cells were collected as described above. Samples were then boiled at 95 °C and analysed by 4–20% SDS–PAGE and transferred to a 0.2-μm PVDF membrane. Anti-HA antibody (Cell Signaling Technology, 3724) and anti-RpoA antibody (BioLegend, catalogue no. 663104) were used at a final concentration of 1:1,000 and 1:2,500 respectively.

To blot for Gp0.4, single colonies of *E. coli* MG1655 or MG1655 *ftsZ9* harbouring pBAD33-gp0.4-HA and pKVS45-ftsZ(Q47K) were grown overnight in M9-glucose at 37 °C. Cultures were back-diluted 1:50 in fresh M9 and grown to OD_600_ of ~0.3 at 37 °C. Cells were induced with 0.2% arabinose for 30 min and processed as described above.

### Selection and sequencing of Gp0.4 suppressors

Single colonies of *E. coli* MG1655 containing pBAD33-gp0.4(ts-ori) with a temperature-sensitive origin of replication were grown overnight in LB–glucose at 30 °C. A total of 100 μl overnight cells were plated directly onto M9L plates supplemented with 0.2% arabinose and incubated at 30 °C overnight. Single colonies on the plate were streaked out on M9L plates and incubated at 30 °C again. To cure the plasmid, single colonies were inoculated in 3 ml LB and grow at 37 °C for 6 h to saturation, incubated at 42 °C for 30 min and then plated on LB plate at 37 °C overnight. Loss of the temperature-sensitive plasmid was confirmed by streaking on antibiotic-containing plates. To sequence the suppressor strains, *E. coli* genomic DNA was extracted using PureLink Genomic DNA kit (Invitrogen) following the manufacturer’s instructions. Extracted genomic DNAs were sequenced by SeqCenter using Illumina whole genome sequencing.

### Microscopy

Single colonies of *E. coli* MG1655 or MG1655 *ftsZ9* containing pBAD33-gp0.4 were grown in M9-glucose overnight at 37 °C. Overnight cultures were back-diluted to OD_600_ of 0.05 in 25 ml of M9-glucose and grown to OD_600_ of ~0.2 at 37 °C. Cells were pelleted at 4,000*g* for 5 min at 4 °C and washed once with M9 (no glucose) and then back-diluted to OD_600_ of 0.1 in 10 ml M9 (no glucose) and recovered for 30 min at 37 °C. At the beginning of the experiment, cells were induced with 0.2% arabinose. At each indicated time point (0, 60 and 120 min), 0.5 ml of cells were pelleted at 4,000*g* for 3 min at 4 °C and resuspended in fresh M9 to OD_600_ = of 0.5. A total of 2 μl of resuspended cells were spotted onto a 1.5% agarose pad prepared with M9 medium placed on cover glasses (VWR). Phase-contrast images were taken on a Zeiss Axio Observer microscope using a 100×/1.4 oil immersion objective and an light-emitting-diode-based illumination system using MetaMorph software (Molecular Devices).

### Growth curves measured by plate reader

Single colonies of *E. coli* MG1655 containing pBR322-EV or pBR322-capRel^Ebc^ and pBAD33-gp0.4 (wild type or the corresponding variants) were grown in M9-glucose overnight at 37 °C. Cultures were then back-diluted 1:20 in M9-glucose and grown overnight again at 37 °C to saturation. Overnight cultures were back-diluted 1:500 in fresh M9 (no glucose) supplemented with 0.2% arabinose, and 160 μl of cells were added into each well of a 96-well plate. A total of 70 μl of oil were added to each well to prevent evaporation, and growth was measured at 15-min intervals with orbital shaking at 37 °C on a plate reader (Biotek). Data reported are the mean and s.d. of 7–10 plate replicates.

### Structure prediction

The structure of Gp0.4 bound to FtsZ was predicted by AlphaFold3^35^ using default settings, with input Gp0.4, FtsZ and GTP as a ligand. The complex structure of Gp0.4, FtsZ and CapRel^Ebc^ was also predicted by AlphaFold3, with ATP included as a substrate.

### Yeast two-hybrid assay

Yeast two-hybrid assays were conducted similarly to the protocol developed previously^44^.

To test the interaction between Gp0.4 and FtsZ, plasmids pGAD-AD-ftsZ (with a (GGGGS)_2_ linker in between AD and FtsZ) and pGBDU-gp0.4-BD (with a (GGGGS)_2_ linker in between) were cotransformed into the yeast *S. cerevisiae* strain PJ69-4A, which has *his3* gene under the control of the *gal1* promoter and the *lacZ* gene under the control of *gal7* promoter. Single colonies were streaked onto SD plates supplemented with complete supplement mixture minus LEU and URA (for selection of plasmids) and plates minus LEU, URA and HIS (for assaying interaction). Plates were grown at 30 °C for 2 days before imaging.

The β-galactosidase assays were conducted using the yeast β-galactosidase assay kit (Thermo Scientific) per the manufacturer’s instructions. In brief, single colonies of yeast strains PJ69-4A harbouring plasmids pGAD-AD-ftsZ and pGBDU-gp0.4-BD were grown in complete supplement mixture media minus LEU and URA overnight at 30 °C to mid-log phase. The OD_600_ of each overnight culture was measured, and 350 μl of each culture was transferred to an Eppendorf tube. A total of 350 μl of the working reagent (1:1 volume ratio of the assay buffer and Y-PER reagent) was added to the tube. After 2 min, 300 μl of the stop solution was added to the tube and vortexed for 15 s. Cell debris was pelleted by centrifuging at 13,000*g* for 30 s, and the absorbance at 420 nm was measured in a cuvette. The β-galactosidase activity was calculated by multiplying 1,000 × *A*_420_/(product of time (in min) × volume of cells (ml) × OD_660_). Data reported are three to four biological replicates.

To test the interaction between CapRel^Ebc^ and Gp0.4 in the presence of FtsZ, plasmids pGAD-gp0.4-AD_ftsZ (with a (GGGGS)_2_ linker between Gp0.4 and AD; both Gp0.4-AD and FtsZ are under the ADH-1 promoter) and pGBDU-BD-capRel^Ebc^ (with a (GGGGS)_2_ linker in between) were cotransformed into the yeast *S. cerevisiae* strain PJ69-4A and streaked onto plates as described above. The control strains contained plasmids pGAD-AD-ftsZ and pGBDU-BD-capRel^Ebc^ or pGAD-gp0.4-AD and pGBDU-BD-capRel^Ebc^. Plates were grown at 30 °C for 3 days before imaging.

### Protein expression and purification

CapRel^Ebc^ was produced from *E. coli* BL21(DE3) cells transformed with pET24d-His_10_-SUMO-capRel^Ebc^ and grown in LB broth supplemented with 0.1% Glu. Protein expression was induced by the addition of 0.5 mM isopropyl-β-d-thiogalactopyranoside (IPTG), and the cells were grown for 4 h at 28 °C shaking at 180 rpm. The culture was centrifuged at 4,000*g* for 15 min at 4 °C and the cell pellet resuspended in lysis buffer (50 mM Tris–HCl pH 8.0, 500 mM NaCl, 750 mM KCl, 2 mM MgCl_2_, 1 mM tris(2-carboxyethyl)phosphine (TCEP), 0.002% mellitic acid) supplemented with a protease inhibitor cocktail, EDTA-free (Merck). Cells were treated with RNase A and DNase (0.1% v/v each) and lysed using a Multishot cell disruptor (Constant Systems). Lysates were clarified by centrifugation (46,500*g*, 50 min, 4 °C, Beckman Coulter), and the supernatant was filtered (0.45 µm) before loading onto a gravity-flow column (Bio-Rad) packed with 2 ml of HisPur Ni-NTA resin (ThermoScientific) pre-equilibrated with 10 column volumes of lysis buffer. The protein was washed with 20 column volumes of buffer A (25 mM Tris–HCl pH 8.0, 500 mM NaCl, 500 mM KCl, 2 mM MgCl_2_, 1 mM TCEP, 0.002% mellitic acid, 20 mM imidazole) and eluted with buffer B (same composition as buffer A but containing 350 mM imidazole). Eluted proteins were further purified by size-exclusion chromatography (SEC) using a HiLoad 16/600 Superdex 75 pg column (Cytiva) equilibrated in SEC buffer (50 mM HEPES pH 7.6, 500 mM NaCl, 500 mM KCl, 2 mM MgCl_2_, 1 mM TCEP, 0.002% mellitic acid). Fractions containing the protein of interest were pooled and concentrated to 1 mg ml^−1^. The His_10_-SUMO tag was cleaved by incubation with His-tagged Ulp1 protease at a 1:1,000 molar ratio (Ulp1:CapRel). Cleaved CapRel was separated from remaining contaminants by re-loading onto the HisPur Ni-NTA gravity column and collecting the flow-through in buffer A. A final SEC step was performed to polish the sample. Protein purity was assessed by SDS–PAGE.

To produce Strep-tagged FtsZ(L178E), *E. coli* BL21(DE3) cells were transformed with pET24d-ftsZ(L178E)-Strep and grown in (LB + 0.1% Glu) medium to OD_600_ of 0.6. Protein expression was induced by the addition of 0.5 mM IPTG, and cells were grown for 4 h at 28 °C shaking at 180 rpm. The culture was centrifuged at 4,000*g* for 15 min at 4 °C and the cell pellet resuspended in lysis buffer (100 mM Tris–HCl pH 8.0, 250 mM NaCl, 250 mM KCl, 2 mM MgCl_2_, 1 mM TCEP, 0.002% mellitic acid) supplemented with a protease inhibitor cocktail, EDTA-free (Merck). Cells were treated with RNase A and DNase (0.1% v/v each) and lysed using a Multishot cell disruptor (Constant Systems). Lysates were clarified by centrifugation (46,500*g*, 50 min, 4 °C, Beckman Coulter), and the supernatant was filtered (0.45 µm) before loading onto a StrepTrap XT prepacked chromatography column (Cytiva) pre-equilibrated with 15 column volumes of lysis buffer. Proteins were washed with 20 column volumes of buffer W (100 mM Tris–HCl pH 8.0, 250 mM NaCl, 250 mM KCl, 2 mM MgCl_2_, 1 mM TCEP, 0.002% mellitic acid) and eluted with elution buffer (same composition as buffer W but supplemented with 50 mM biotin). Eluted proteins were further purified by SEC using a HiLoad 16/600 Superdex 75 pg column (Cytiva) equilibrated in SEC buffer (50 mM HEPES pH 7.6, 500 mM NaCl, 500 mM KCl, 2 mM MgCl_2_, 1 mM TCEP, 0.002% mellitic acid). Fractions containing the protein of interest were pooled and concentrated. Protein purity was assessed by SDS–PAGE.

Gp0.4 and Gp0.4(L35A) were chemically synthesized (https://www.peptide.com/) and obtained as a powder at >90% purity. The lyophilized powder was dissolved in 25 mM CAPS pH 11, 200 mM NaCl, 1 mM TCEP, 2 mM MgCl_2_, 0.002% mellitic acid to a concentration of 1–1.5 mg ml^−1^. The solubilized protein was diluted fivefold in 125 mM Tris pH 8.5, 200 mM NaCl, 1.25% DMSO, 1 mM TCEP, 2 mM MgCl_2_, 0.002% mellitic acid and dialysed against SEC buffer 50 mM HEPES pH 7.6, 500 mM NaCl, 500 mM KCl, 2 mM MgCl_2_, 1 mM TCEP, 0.002% mellitic acid and concentrated for biophysical experiments.

### Cell-free transcription–translation assay

Cell-free translation reactions were performed using the PURExpress in vitro protein synthesis kit (New England Biolabs) according to the manufacturer’s instructions. CapRel^Ebc^, FtsZ(L178E) and Gp0.4 (and Gp0.4 (L35A)) were added to the reactions at final concentrations of 1 µM, 8 µM and 12 µM, respectively. An RNase inhibitor (New England Biolabs) was included as recommended by manufacturer protocol to prevent RNA degradation. All components except the DHFR-encoding DNA template were assembled on ice, followed by preincubation at 37 °C for 5 min. Template DNA was then added to initiate translation. Nuclease-free water was added in place of DNA as negative control, and all samples were further incubated at 37 °C for 40 min. Reactions were quenched by addition of Laemmli sample buffer, heated and analysed by SDS–PAGE, and de novo-synthesized DHFR was visualized by Coomassie Brilliant Blue staining.

### HDX-MS

HDX-MS samples were prepared by concentrating CapRel^Ebc^, Gp0.4 and FtsZ(L178E) to 250 μM and mixing 4 µl of sample with 56 µl of the labelling buffer L (50 mM HEPES pH 7.6, 500 mM NaCl, 500 mM KCl, 2 mM MgCl_2_, 1 mM TCEP, 0.002% mellitic acid). To reconstitute the CapRel^Ebc^–Gp0.4–FtsZ(L178E) complex, concentrated samples of CapRel^Ebc^, Gp0.4, FtsZ(178E) were mixed to a final concentration of complex of 250 µM. We then mixed 4 µl of the complex with 54 µl of the labelling buffer L (50 mM HEPES pH 7.6, 500 mM NaCl, 500 mM KCl, 2 mM MgCl_2_, 1 mM TCEP, 0.002% mellitic acid) and incubated during the labelling time at 20 °C on a heat block. Non-deuterated reference points were prepared by replacement of buffer L with equilibration buffer E (50 mM HEPES, 500 mM KCl, 500 mM NaCl, 2 mM MgCl_2_, 1 mM TCEP, 0.002% mellitic acid, pH 7.5). After incubation, samples were quenched with an equivalent volume of quench buffer (1.2% formic acid, 1 mM TCEP, pH 2.4) and immediately flash-frozen in liquid nitrogen. Before each injection, quenched samples were thawed at room temperature and transferred to the Enzymate BEH Pepsin Column (Waters Corporation), where digestion occurred at 200 µl min^−1^ under 7 kPSI pressure at 20 °C. Peptic peptides were trapped for 3 min on an Acquity Ultraperformance Liquid Chromatography (UPLC) BEH C18 VanGuard Pre-column (Waters Corporation) at 200 µl min^−1^ using MS-grade water containing 0.1% formic acid, and then eluted on an Acquity UPLC BEH C18 Column (Waters Corporation) with a linear gradient (3–45%) of 0.1% formic acid in acetonitrile at 40 µl min^−1^.

At the end of each run, columns were cleaned as follows: for the enzymatic column, three injections of pepsin wash buffer (1.5 M Gu-HCl, 4% (v/v) acetonitrile (ACN), 0.8% (v/v) formic acid); for the pre-column and UPLC column, a saw-tooth gradient was applied. A cleaning run was also performed every three samples to prevent peptide carry-over. Peptide identification was performed on non-deuterated samples (five replicates) using the Synapt G2 mass spectrometer with positive ionization and an ESI probe in MSE acquisition mode (collision energy ramp in the transfer cell: 20–30 eV). Leucine Enkephalin was used for mass accuracy correction, and sodium formate served as the calibration standard. All deuterium labelling time points were performed in triplicate. The non-deuterated reference points were analysed using PLGS (ProteinLynx Global Server 2.5.1, Waters Corporation) to identify peptic peptides corresponding to CapRel^Ebc^, Gp0.4 or FtsZ(L178E). All MSE data, including reference and deuterated samples, were processed with DynamX 3.0 (Waters Corporation) for deuterium uptake determination. The following filtering parameters were chosen to optimize the balance between peptide quality and coverage: minimum intensity of 1,000; peptide sequence length between 5 and 20 amino acids; at least 3 MS/MS products; a minimum of 0.27 products per amino acid; a minimum score of 5; and a maximum MH^+^ error threshold of 15 ppm. Data were analysed at both the peptide and overall levels. All heat maps were generated using DynamX, and Deuteros software was also used for statistical analysis.

### ITC

ITC measurements were performed using an Affinity ITC microcalorimeter (TA Instruments) at 35 °C. For the FtsZ(L178E)-Gp0.4 titration, FtsZ(L178E) (360 µM) was loaded into the syringe and titrated into the cell containing Gp0.4 (30 µM). To characterize the formation of the ternary complex CapRel^Ebc^–FtsZ(L178E)–Gp0.4, CapRel^Ebc^ (200 µM) was titrated into a mixture of FtsZ(L178E) and Gp0.4 (20 µM). All titrations were carried out in buffer containing 50 mM HEPES pH 7.6, 500 mM KCl, 500 mM NaCl, 2 mM MgCl_2_, 0.002% mellitic acid and 1 mM TCEP. Protein concentrations were determined by absorbance measurements using a NanoDrop One spectrophotometer (Thermo Scientific). Titrations were performed by successive injections of 2 µl titrant at a stirring rate of 75 rpm. Raw data were corrected for buffer contributions and analysed using NanoAnalyze (TA Instruments) and Origin (OriginLab).

### Statistics and reproducibility

No statistical method was used to predetermine sample size. Sample sizes were chosen based on the number needed to reliably determine differences between groups. Given large effect sizes, we chose to replicate experiments two to three times as is routine to indicate reproducibility. No data were excluded from analysis. No experimental groups or control groups were subjectively chosen, and there are no covariates to control for as experiments were done in isogenic strains. No randomization is required. Blinding was not required because all data were obtained objectively and had strong effect sizes over multiple independent replicates and raw data are reported in the manuscript.

### Reporting summary

Further information on research design is available in the Nature Portfolio Reporting Summary linked to this article.

## Supplementary information

Supplementary Information Supplementary Tables 1–3.

Reporting Summary

Peer Review File

## Source data

Source Data Fig. 1 Unprocessed western blots and gels for all gels.

Source Data Fig. 2 Statistical source data for figures.

## Figures and Tables

**Figure:**
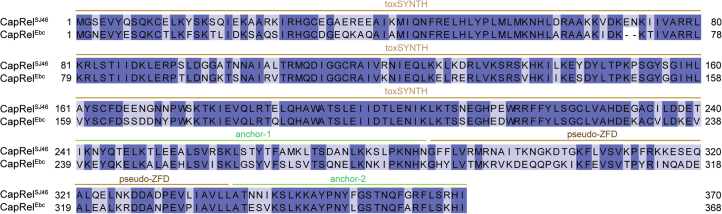
Extended Data Fig. 7 **Sequence alignment of CapRel**^**Ebc**^ **and CapRel**^**SJ46**^ **with domains labelled.**

## Data Availability

The mass spectrometry proteomics data have been deposited to the ProteomeXchange Consortium via the PRIDE partner repository with the dataset identifier PXD065182. Sequencing data are available in the Sequence Read Archive under BioProject PRJNA1452550. All other data are available in the Article or its Supplementary Information. Materials, including strains and plasmids, are available on reasonable request. Source data are provided with this paper.
