# Supplementary material for: Bacterial cell division protein FtsZ complexes with a phage protein to activate bacterial immunity
Source: Nat Microbiol. 2026 Jun 12;11(8):2266–78. doi: 10.1038/s41564-026-02384-6 (PMC13423816; doi:10.1038/s41564-026-02384-6)
Supplement: Supplementary file 1 — Supplementary Tables 1–3. [file 41564_2026_2384_MOESM1_ESM.pdf]

nature microbiology

Supplementary information

https://doi.org/10.1038/s41564-026-02384-6

Bacterial cell division protein FtsZ complexes with a phage protein to activate bacterial immunity

In the format provided by the authors and unedited

Table S1. Strains

Bacterial Strains

**Name**	**Genotype**	**Source**
ML6	MG1655	
	DH5α	Invitrogen
ML3836	BW27783	
	BL21 (DE3)	Lab stock
ML3840	MG1655 pBR322-EV	Zhang et al., 2022
ML3842	MG1655 pBR322-*capRel*^{Ebc}	Zhang et al., 2022
ML3845	MG1655 pBR322-*capRel*^{Ebc}*(Y153A)*	Zhang et al., 2022
ML4939	MG1655 pBAD33-*gp0.4* pBR322-EV	this study
ML4940	MG1655 pBAD33-*gp0.4* pBR322-*capRel*^{Ebc}	this study
ML4941	MG1655 pBAD33-*gp0.4* pBR322-*capRel*^{Ebc}*(Y153A)*	this study
ML4942	MG1655 pBAD33-*capRel*^{Ebc}*(1-270)* pEXT20-EV	this study
ML4943	MG1655 pBAD33-*capRel*^{Ebc}*(1-270)* pEXT20-*capRel*^{Ebc}*(271-369)*	this study
ML4944	MG1655 pBR322-*His*$_6$-*capRel*^{Ebc}	this study
ML4945	MG1655 pBAD33-*gp0.4*	this study
ML4946	MG1655 *ftsZ9* pBAD33-*gp0.4*	this study
ML4947	MG1655 *ftsZ9* pBAD33-*gp0.4* pBR322-EV	this study
ML4948	MG1655 *ftsZ9* pBAD33-*gp0.4* pBR322-*capRel*^{Ebc}	this study
ML4949	MG1655 *ftsZ9* pBAD33-*gp0.4* pBR322-*capRel*^{Ebc}*(Y153A)*	this study
ML4950	MG1655 *ftsZ9* pBR322-EV	this study
ML4951	MG1655 *ftsZ9* pBR322-*capRel*^{Ebc}	this study
ML4952	MG1655 pBAD33-*sulA* pBR322-EV	this study
ML4953	MG1655 pBAD33-*sulA* pBR322-*capRel*^{Ebc}	this study
ML4954	MG1655 pBAD33-*kil*$^{\lambda}$ pBR322-EV	this study
ML4955	MG1655 pBAD33-*kil*$^{\lambda}$ pBR322-*capRel*^{Ebc}	this study
ML4956	MG1655 pBAD33-*kil*^{Rac} pBR322-EV	this study
ML4957	MG1655 pBAD33-*kil*^{Rac} pBR322-*capRel*^{Ebc}	this study

ML4958	BW27783 pBAD33-EV	this study
ML4959	BW27783 pBAD33-*gp0.4*	this study
ML4960	BW27783 pBAD33-*gp0.4(W28L)*	this study
ML4961	BW27783 pBAD33-*gp0.4(L35P)*	this study
ML4962	MG1655 *ftsZ9* pBAD33-*gp0.4* pBR322-EV pKVS45-EV	this study
ML4963	MG1655 *ftsZ9* pBAD33-*gp0.4* pBR322-*capRel*^{Ebc} pKVS45-EV	this study
ML4964	MG1655 *ftsZ9* pBAD33-*gp0.4* pBR322-*capRel*^{Ebc}*(Y153A)* pKVS45-EV	this study
ML4965	MG1655 *ftsZ9* pBAD33-*gp0.4* pBR322-EV pKVS45-*ftsZ(Q47K)*	this study
ML4966	MG1655 *ftsZ9* pBAD33-*gp0.4* pBR322-*capRel*^{Ebc} pKVS45-*ftsZ(Q47K)*	this study
ML4967	MG1655 *ftsZ9* pBAD33-*gp0.4* pBR322-*capRel*^{Ebc}*(Y153A)* pKVS45-*ftsZ(Q47K)*	this study
ML4968	MG1655 *ftsZ9* pBAD33-*gp0.4* pBR322-EV pKVS45-*ftsZ(Q47K Δ2-9)*	this study
ML4969	MG1655 *ftsZ9* pBAD33-*gp0.4* pBR322-EV pKVS45-*ftsZ(Q47K D269K)*	this study
ML4970	MG1655 *ftsZ9* pBAD33-*gp0.4* pBR322-*capRel*^{Ebc} pKVS45-*ftsZ(Q47K Δ2-9)*	this study
ML4971	MG1655 *ftsZ9* pBAD33-*gp0.4* pBR322-*capRel*^{Ebc} pKVS45-*ftsZ(Q47K D269K)*	this study
ML4972	DH5α pBAD33-*gp0.4(V15A)*	this study
ML4973	DH5α pBAD33-*gp0.4(V22A)*	this study
ML4974	DH5α pBAD33-*gp0.4(W28A)*	this study
ML4975	DH5α pBAD33-*gp0.4(M32A)*	this study
ML4976	DH5α pBAD33-*gp0.4(L35A)*	this study
ML4977	DH5α pBAD33-*gp0.4(Y39A)*	this study
ML4978	DH5α pBAD33-*gp0.4(A12K)*	this study
ML4979	DH5α pBAD33-*gp0.4(T14S)*	this study
ML4980	DH5α pBAD33-*gp0.4(L16A)*	this study
ML4981	DH5α pBAD33-*gp0.4(S19A)*	this study
ML4982	DH5α pBAD33-*gp0.4(R23A)*	this study
ML4983	DH5α pBAD33-*gp0.4(K36A)*	this study
ML4984	DH5α pKVS45-EV	this study

ML4985	DH5α pKVS45-*ftsZ(Q47K)*	this study
ML4986	DH5α pKVS45-*ftsZ(Q47K Δ2-9)*	this study
ML4987	DH5α pKVS45-*ftsZ(Q47K D269K)*	this study
ML4988	DH5α pKVS45-*ftsZ(D209C)*	this study
ML4989	DH5α pKVS45-*ftsZ(L178E)*	this study
ML4990	DH5α pKVS45-*ftsZ(L178E Δ2-9)*	this study
ML4991	DH5α pKVS45-*ftsZ(L178E Δ317-383)*	this study
ML4992	DH5α pKVS45-*ftsZ(L178E Δ2-9 Δ317-383)*	this study
ML4993	DH5α pKVS45-*ftsZ(L178E D269K)*	this study
ML4994	DH5α pBAD33-$capRel^{Ebc}(1\text{-}270)$	this study
ML4995	DH5α pEXT20-$capRel^{Ebc}(271\text{-}369)$	this study
ML4996	DH5α pBAD33-*sulA*	this study
ML4997	DH5α pBAD33-kil^{λ}	this study
ML4998	DH5α pBAD33-kil^{Rac}	this study
ML4999	BL21(DE3) pET24d-His_{10}-*SUMO*-$capRel^{Ebc}$	this study
ML5000	BL21(DE3) pET24d-*ftsZ(L178E)-Strep*	this study
ML5001	DH5α pGBDU-*relB-BD*	this study
ML5002	DH5α pGBDU-*gp0.4-BD*	this study
ML5003	DH5α pGBDU-*BD-EV*	this study
ML5004	DH5α pGBDU-*gp0.4(W28L)-BD*	this study
ML5005	DH5α pGBDU-*gp0.4(L35P)-BD*	this study
ML5006	DH5α pGAD-*AD-relE*	this study
ML5007	DH5α pGAD-*AD-EV*	this study
ML5008	DH5α pGAD-*AD-ftsZ*	this study
ML5009	DH5α pGBDU-*gp0.4(T14S)-BD*	this study
ML5010	DH5α pGBDU-*gp0.4(A12K)-BD*	this study
ML5011	DH5α pGBDU-*gp0.4(L16A)-BD*	this study
ML5012	DH5α pGBDU-*gp0.4(S19A)-BD*	this study
ML5013	DH5α pGBDU-*gp0.4(R23A)-BD*	this study
ML5014	DH5α pGBDU-*gp0.4(K36A)-BD*	this study
ML5015	DH5α pGAD-*gp0.4-AD*	this study

ML5016	DH5α pGAD-*ftsZ*	this study
ML5017	DH5α pGAD-*gp0.4-AD_ftsZ*	this study
ML5018	DH5α pGBDU-*BD-capRel*^{Ebc}	this study
ML5019	DH5α pGAD-*gp0.4-AD_ftsZ(Q47K)*	this study
ML5020	DH5α pGAD-*gp0.4-AD_ftsZ(D209C)*	this study
ML5021	DH5α pGAD-*gp0.4(W28L)-AD_ftsZ*	this study
ML5022	DH5α pGAD-*gp0.4(L35P)-AD_ftsZ*	this study

Yeast Strains

ML5023	*Saccharomyces cerevisiae* strain PJ69-4A	this study

Phage Strains

**Name**	**Genotype**	**Source**
phML1	T2	ATCC Cat #: 11303-B2
phML2	T3	ATCC Cat #: 11303-B3
phML3	T4	ATCC Cat #: 11303-B4
phML4	T5	ATCC Cat #: 11303-B5
phML5	T6	ATCC Cat #: 11303-B6
phML6	T7	Gift from R. Sorek
phML7	RB69	Laval Collection, HER #158
phML8	λ_{vir}	Gift from R. Sorek
phML9	SECΦ17	(Doron et al., 2018)
phML10	SECΦ18	(Doron et al., 2018)
phML11	SECΦ27	(Doron et al., 2018)
phML12	Lust	(Malki et al., 2016)
phML103	Bas64	(Maffei et al., 2021)
phML104	Bas65	(Maffei et al., 2021)
phML105	Bas66	(Maffei et al., 2021)
phML106	Bas67	(Maffei et al., 2021)
phML107	Bas68	(Maffei et al., 2021)
phML108	T7 evolved clone 1 containing Gp0.4(L35P)	this study

phML109	T7 evolved clone 2 containing Gp0.4(W28L)	this study
phML110	T7 evolved clone 3 containing frameshift	this study

Table S2. Plasmids

Plasmid	Description	Source
pBAD33-EV	empty vector of pBAD33 (p15A ori, P_{ara} promoter)	lab stock
pEXT20-EV	empty vector of pEXT20 (pBR322 ori, P_{lac} promoter)	lab stock
pBR322-EV	derivative of pBR322 with P_{tet} removed	lab stock
pBR322-*capRel*^{Ebc}	*capRel*^{Ebc} with native promoter	Zhang et al., 2022
pBR322-*capRel*^{Ebc}*(Y153A)*	*capRel*^{Ebc}*(Y153A)* with native promoter	Zhang et al., 2022
pBAD33-*gp0.4*	arabinose inducible *gp0.4*	this study
pBAD33-*capRel*^{Ebc}*(1-270)*	arabinose inducible N-terminal fragment (1-270) of CapRel^{Ebc}	this study
pEXT20-*capRel*^{Ebc}*(271-369)*	IPTG inducible C-terminal fragment (271-369) of CapRel^{Ebc}	this study
pBR322-*His*$_6$*-capRel*^{Ebc}	*capRel*^{Ebc} with N-terminal *His*$_6$*-tag*	this study
pBAD33-*sulA*	arabinose inducible *sulA*	this study
pBAD33-*kil*$^{\lambda}$	arabinose inducible *kil*$^{\lambda}$	this study
pBAD33-*kil*^{Rac}	arabinose inducible *kil*^{Rac}	this study
pBAD33-*gp0.4(W28L)*	arabinose inducible *gp0.4(W28L)*	this study
pBAD33-*gp0.4(L35P)*	arabinose inducible *gp0.4(L35P)*	this study
pKVS45-EV	empty vector of pKVS45 (SC101 ori, P_{tet} promoter)	this study
pKVS45-*ftsZ(Q47K)*	*ftsZ(Q47K)* in pKVS45	this study
pKVS45-*ftsZ(Q47K Δ2-9)*	*ftsZ(Q47K Δ2-9)* in pKVS45	this study
pKVS45-*ftsZ(Q47K D269K)*	*ftsZ(Q47K D269K)* in pKVS45	this study
pKVS45-*ftsZ(D209C)*	*ftsZ(D209C)* in pKVS45	this study
pKVS45-*ftsZ(L178E)*	*ftsZ(L178E)* in pKVS45	this study
pKVS45-*ftsZ(L178E Δ2-9)*	*ftsZ(L178E Δ2-9)* in pKVS45	this study
pKVS45-*ftsZ(L178E Δ317-383)*	*ftsZ(L178E Δ317-383)* in pKVS45	this study
pKVS45-*ftsZ(L178E Δ2-9 Δ317-383)*	*ftsZ(L178E Δ2-9 Δ317-383)* in pKVS45	this study

pKVS45-*ftsZ(L178E D269K)*	*ftsZ(L178E D269K)* in pKVS45	this study
pBAD33-*gp0.4(V15A)*	arabinose inducible *gp0.4(V15A)*	this study
pBAD33-*gp0.4(V22A)*	arabinose inducible *gp0.4(V22A)*	this study
pBAD33-*gp0.4(W28A)*	arabinose inducible *gp0.4(W28A)*	this study
pBAD33-*gp0.4(M32A)*	arabinose inducible *gp0.4(M32A)*	this study
pBAD33-*gp0.4(L35A)*	arabinose inducible *gp0.4(L35A)*	this study
pBAD33-*gp0.4(Y39A)*	arabinose inducible *gp0.4(Y39A)*	this study
pBAD33-*gp0.4(A12K)*	arabinose inducible *gp0.4(A12K)*	this study
pBAD33-*gp0.4(T14S)*	arabinose inducible *gp0.4(T14S)*	this study
pBAD33-*gp0.4(L16A)*	arabinose inducible *gp0.4(L16A)*	this study
pBAD33-*gp0.4(S19A)*	arabinose inducible *gp0.4(S19A)*	this study
pBAD33-*gp0.4(R23A)*	arabinose inducible *gp0.4(R23A)*	this study
pBAD33-*gp0.4(K36A)*	arabinose inducible *gp0.4(K36A)*	this study
pET24d-His_{10}-*SUMO-capRel*^{Ebc}	expression vector of His_{10}-*SUMO-capRel*^{Ebc}	this study
pET24d-*ftsZ(L178E)-Strep*	expression vector of *ftsZ(L178E)-Strep*	this study
pGBDU-*relB-BD*	*relB-BD* in pGBDU vector	this study
pGBDU-*gp0.4-BD*	*gp0.4-BD* in pGBDU vector	this study
pGBDU-*BD-EV*	pGBDU empty vector with GAL4-BD	this study
pGBDU-*gp0.4(W28L)-BD*	*gp0.4(W28L)-BD* in pGBDU vector	this study
pGBDU-*gp0.4(L35P)-BD*	*gp0.4(L35P)-BD* in pGBDU vector	this study
pGAD-*AD-relE*	*AD-relE* in pGAD vector	this study
pGAD-*AD-EV*	pGAD empty vector with GAL4-AD	this study
pGAD-*AD-ftsZ*	*AD-ftsZ* in pGAD vector	this study
pGBDU-*gp0.4(T14S)-BD*	*gp0.4(T14S)-BD* in pGBDU vector	this study
pGBDU-*gp0.4(A12K)-BD*	*gp0.4(A12K)-BD* in pGBDU vector	this study
pGBDU-*gp0.4(L16A)-BD*	*gp0.4(L16A)-BD* in pGBDU vector	this study
pGBDU-*gp0.4(S19A)-BD*	*gp0.4(S19A)-BD* in pGBDU vector	this study
pGBDU-*gp0.4(R23A)-BD*	*gp0.4(R23A)-BD* in pGBDU vector	this study
pGBDU-*gp0.4(K36A)-BD*	*gp0.4(K36A)-BD* in pGBDU vector	this study
pGAD-*gp0.4-AD*	*gp0.4-AD* in pGAD vector	this study
pGAD-*ftsZ*	*ftsZ* in pGAD vector	this study

pGAD-*gp0.4-AD_ftsZ*	*gp0.4-AD* and *ftsZ* in pGAD vector	this study
pGBDU-*BD-capRel*^{Ebc}	*BD-capRel*^{Ebc} in pGBDU vector	this study
pGAD-*gp0.4-AD_ftsZ(Q47K)*	*gp0.4-AD* and *ftsZ(Q47K)* in pGAD vector	this study
pGAD-*gp0.4-AD_ftsZ(D209C)*	*gp0.4-AD* and *ftsZ(D209C)* in pGAD vector	this study
pGAD-*gp0.4(W28L)-AD_ftsZ*	*gp0.4(W28L)-AD* and *ftsZ* in pGAD vector	this study
pGAD-*gp0.4(L35P)-AD_ftsZ*	*gp0.4(L35P)-AD* and *ftsZ* in pGAD vector	this study

Table S3. Primers

Name	Purpose	Sequence (5'-3')
TZ-1	linearize pBAD33	AAGCTTGGCTGTTTTGGC
TZ-2	linearize pBAD33	CTCGAATTCGCTAGCCCAAA
TZ-3	*gp0.4* into pBAD33	TACCCGTTTTTTTGGGCTAGCGAATTCGAG ATGTCTACTACCAACGTGCAATACG
TZ-4	*gp0.4* into pBAD33	CTTCTCTCATCCGCCAAAACAGCCAAGCTT TCACTCAGCAGATTCTAAAGCTATTGC
TZ-5	kil^{Rac} into pBAD33	TACCCGTTTTTTTGGGCTAGCGAATTCGAG ATGATTGCACATCACTTCGGAACT
TZ-6	kil^{Rac} into pBAD33	CTTCTCTCATCCGCCAAAACAGCCAAGCTT TCACCATGACTCCGCCTTTAC
TZ-7	kil^{λ} into pBAD33	TACCCGTTTTTTTGGGCTAGCGAATTCGAG ATGGATCAAACACTTATGGCTATCCA
TZ-8	kil^{λ} into pBAD33	CTTCTCTCATCCGCCAAAACAGCCAAGCTT TTAGTGAATGCTTTTGCTTGATCTCAGT
TZ-9	*sulA* into pBAD33	TACCCGTTTTTTTGGGCTAGCGAATTCGAG ATGTACACTTCAGGCTATGCACATC
TZ-10	*sulA* into pBAD33	TCTCATCCGCCAAAACAGCCAAGCTTTTAA TGATACAAATTAGAGTGAATTTTTAGCCCG
TZ-11	add strong RBS to pBAD33-*sulA*	CTTTAAAAGGCAAAAACTAATTTATGTACA CTTCAGGCTATGCACATC
TZ-12	add strong RBS to pBAD33-*sulA*	GTTTTTGCCTTTTAAAGACGCTAGCCCAAA AAAACGGGTATG
TZ-13	$capRel^{Ebc}(1\text{-}270)$ into pBAD33	TACCCGTTTTTTTGGGCTAGCGAATTCGAGCGGG AGGATATCGGGAATGGG
TZ-14	$capRel^{Ebc}(1\text{-}270)$ into pBAD33	CTTCTCTCATCCGCCAAAACAGCCAAGCTTTTATG AAGTCACGCTAAGTGAAAACACATAACT
TZ-15	linearize pEXT20	CGGGGATCCTCTAGAGTCGAC
TZ-16	linearize pEXT20	GGTACCGAGCTCGAATTCTGTTTC
TZ-17	$capRel^{Ebc}(271\text{-}369)$ into pEXT20	AATTCGAGCTCGGTACCGGGAGGATATCGGGAAT GCAAAACGAACTTAAAAACAAAATTCCAAAAAAC
TZ-18	$capRel^{Ebc}(271\text{-}369)$ into pEXT20	GCAGGTCGACTCTAGAGGATCCCCGTTACTTAAT ATGCTTGCTTAAAAAGCGTG
TZ-19	construct pBR322-His_6-$capRel^{Ebc}$	TCACCATCACCACGGCAGCAGCGGCATGGGCAAT GAAGTTTATGAAAGCC

TZ-20	construct pBR322-*His$_6$-capRel^{Ebc}*	TGCCGTGGTGATGGTGATGATGCATTCCCGATAT CCTCCCGTTAATATTA
TZ-21	*ftsZ* into pKVS45	AGGTTTCTCCATACAGGAGGTACCCATGTTTGAA CCAATGGAACTTACCAATGAC
TZ-22	*ftsZ* into pKVS45	CCTGCAGGTCGACTCTAGAGGATCCTTAATCAGC TTGCTTACGCAGGAA
TZ-23	linearize pKVS45	GGGTACCTCCTGTATGGAGAAACC
TZ-24	linearize pKVS45	GGATCCTCTAGAGTCGACCTGC
TZ-25	construct pBAD33-*gp0.4-BD*	CGATACAGTCAACTGTCTTTGACCT
TZ-26	construct pBAD33-*gp0.4-BD*	GGTGGCGGTGGCTCGGGCGGTGGTGGGTCGATGA AGCTACTGTCTTCTATCGAACA
TZ-27	construct pBAD33-*gp0.4-BD*	AAGGTCAAAGACAGTTGACTGTATCGTGAAAGCT TGGCTGTTTTGGC
TZ-28	construct pBAD33-*gp0.4-BD*	CGACCCACCACCGCCCGAGCCACCGCCACCCTCAG CAGATTCTAAAGCTATTGCC
TZ-29	construct pGBDU-*gp0.4-BD*	AAGCTTGAAGCAAGCCTCCTGAAAGATGTCTACT ACCAACGTGCAATACG
TZ-30	construct pGBDU-*gp0.4-BD*	CTTTCAGGAGGCTTGCTTCAAG
TZ-31	construct pGBDU-*gp0.4-BD*	GGTCAAAGACAGTTGACTGTATCGTGACCGGAAT TCCCG
TZ-32	construct pGAD-*AD-ftsZ*	CGGTGGCTCGGGCGGTGGTGGGTCGATGTTTGAA CCAATGGAACTTACCAATGAC
TZ-33	construct pGAD-*AD-ftsZ*	GATGGATCCCCCGGGGAATTCGATTTAATCAGCT TGCTTACGCAGGAA
TZ-34	construct pGAD-*AD-ftsZ*	CGACCCACCACCGCCCGAGCCACCGCCACCCTCTT TTTTTGGGTTTGGTGGGG
TZ-35	construct pGAD-*AD-ftsZ*	ATCGAATTCCCCGGGGGAT
TZ-36	construct pBAD33-*gp0.4-AD*	ATGGATAAAGCGGAATTAATTCCCGAG
TZ-37	construct pBAD33-*gp0.4-AD*	CTCTTTTTTTGGGTTTGGTGGGG
TZ-38	construct pBAD33-*gp0.4-AD*	TACCCCACCAAACCCAAAAAAAGAGTGAAAGCTT GGCTGTTTTGGC
TZ-39	construct pBAD33-*gp0.4-AD*	CTCGGGAATTAATTCCGCTTTATCCATCGACCCAC CACCGC
TZ-40	construct pGAD-*gp0.4-AD*	ACAATCAACTCCAAGCTTTGCAAAGATGTCTACT ACCAACGTGCAATACG

TZ-41	construct pGAD-*gp0.4-AD*	ATGGATCCCCCGGGGAATTCGATTCACTCTTTTTTGGGTTTGGTGG
TZ-42	construct pGAD-*gp0.4-AD*	CTTTGCAAAGCTTGGAGTTGAT
TZ-43	construct pGAD-*AD-ftsZ*	ACAATCAACTCCAAGCTTTGCAAAGATGTTTGAACCAATGGAACTTACCAATGAC
TZ-44	construct pGAD-*gp0.4-AD_ftsZ*	GTCATGATAATAATGGTTTCTTACCGGTAGAGGTGTGGTCAATAAGA
TZ-45	construct pGAD-*gp0.4-AD_ftsZ*	TGCCACCTGACGTCGCTTGCATGCAACTTCTTTTCTT
TZ-46	construct pGAD-*gp0.4-AD_ftsZ*	AGTTGCATGCAAGCGACGTCAGGTGGCACTTTTCG
TZ-47	construct pGAD-*gp0.4-AD_ftsZ*	CACCTCTACCGGTAAGAAACCATTATTATCATGACATTAACCTAT
