# Supplementary figures and images for: Bacterial cell division protein FtsZ complexes with a phage protein to activate bacterial immunity
Source: Nat Microbiol. 2026 Jun 12;11(8):2266–78. doi: 10.1038/s41564-026-02384-6 (PMC13423816; doi:10.1038/s41564-026-02384-6)

**a Fig. 4b**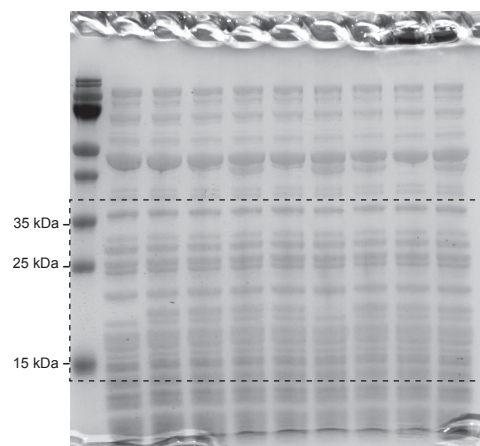**b Fig. 4h**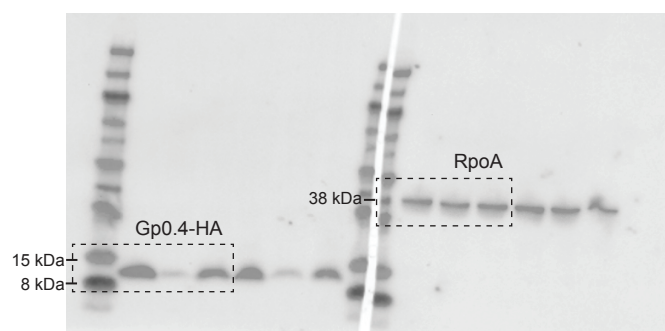**c ED Fig. 1d**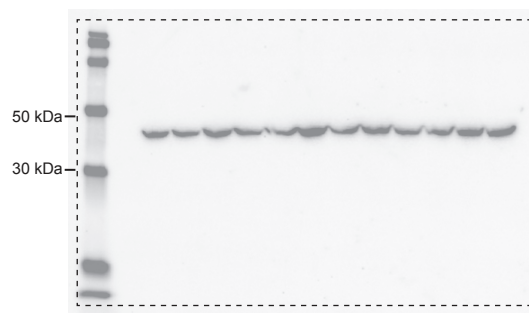**d ED Fig. 4c**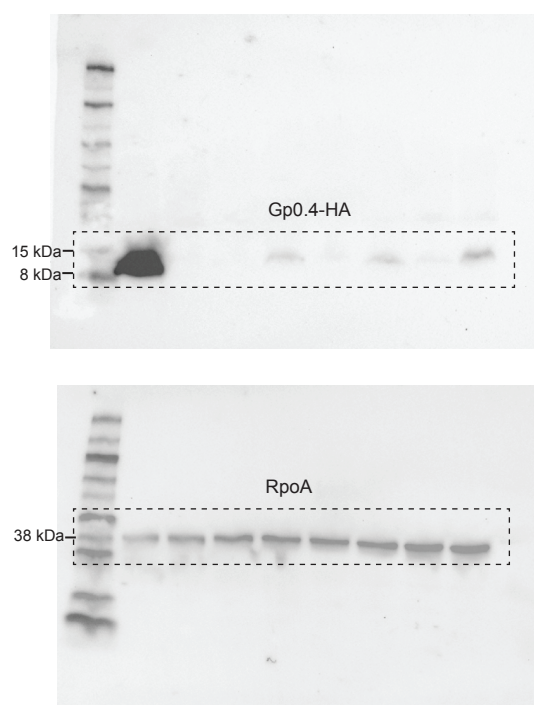**e ED Fig. 5c**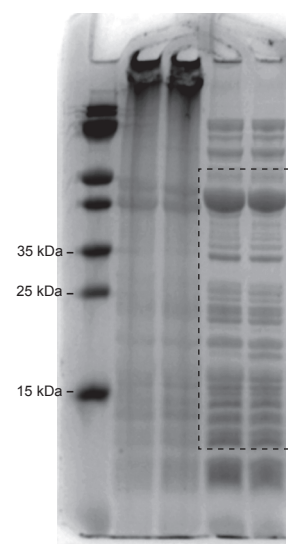

Supplement: Supplementary file 4 — Unprocessed western blots and gels for all gels. [file 41564_2026_2384_MOESM4_ESM.pdf]
