# Supplementary material for: Bacterial cell division protein FtsZ complexes with a phage protein to activate bacterial immunity
Source: Nat Microbiol. 2026 Jun 12;11(8):2266–78. doi: 10.1038/s41564-026-02384-6 (PMC13423816; doi:10.1038/s41564-026-02384-6)
Supplement: Supplementary file 3 — Peer Review File [file 41564_2026_2384_MOESM3_ESM.pdf]

nature portfolio

Peer Review File

Bacterial cell division protein FtsZ complexes with a phage protein to activate bacterial immunity

Corresponding Author: Professor Michael Laub

Version 0:

Reviewer comments:

Reviewer #1

(Remarks to the Author)
In this manuscript, Zhang et al. describe a novel activation mechanism for the bacterial defense protein CapRelEbc from Enterobacter chengduensis, showing that it requires both a phage protein and a host factor for activation. The authors demonstrate that CapRelEbc protects E. coli from T7 infection by detecting a complex between the T7 protein Gp0.4 and the bacterial cell division protein FtsZ. Gp0.4 alone does not activate CapRelEbc; instead, it binds to the synergy loop of FtsZ to block polymerization, and CapRelEbc senses this Gp0.4-FtsZ complex. A mutant FtsZ variant (ftsZ9) that resists Gp0.4 binding abolishes defense activation, and other unrelated FtsZ inhibitors, such as SulA and Kil peptides, fail to trigger CapRelEbc, indicating that FtsZ inhibition alone is insufficient.

Mechanistic dissection through genetic screens, mutational analysis, structural modeling, and HDX reveals that Gp0.4 interacts with both FtsZ and the C-terminal antitoxin domain of CapRelEbc. Yeast two-hybrid assays demonstrate that CapRelEbc and Gp0.4 do not interact in isolation but only in the presence of FtsZ, removing the bacterial genetic background and providing additional confirmation of the ternary complex model.

Overall, this work is elegant and broadens the conceptual framework of bacterial immune sensing and draws interesting parallels to eukaryotic effector-triggered immunity (ETI). The findings suggest that searches for phage defense activators should consider host factors in addition to phage proteins and highlight the rapid functional diversification of related immunity proteins.

Major comments:

1. I believe the manuscript would be strengthened by additional biochemical characterization of the ternary complex in vitro. Since the major novelty of this work is in demonstrating recognition of a phage protein-host factor complex, direct biochemical validation of that complex would reinforce the conclusions. This could include, for example, HDX experiments omitting one component, binding affinity measurements, purification of the intact complex, or in vitro reconstitution assays such as pyrophosphorylation.

2. The data clearly demonstrate the necessity of FtsZ for the recognition of Gp0.4 by CapRelEbc. However, it is not entirely clear how FtsZ becomes essential for this recognition, as the AlphaFold3 model suggests only modest direct contacts between CapRelEbc and FtsZ. Could the binding of FtsZ instead stabilize the conformation of Gp0.4, making it more favorable for interaction with CapRelEbc?

3. CapRelEbc and CapRelSJ46 are highly similar yet differ in binding specificity. Beyond the structural overlay in Fig. S5, a deeper analysis of sequence differences at the interaction interfaces would be helpful. Can the difference be rationalized by the amino acid changes between Ebc and SJ46 at the interaction residues with FtsZ, MCP, or Gp54? Could either protein recognize the other's known phage targets, depending on the identity of the infecting phage? If not, what prevents cross-recognition despite high sequence identity? If possible, it would also be valuable to expand the phage panel to determine whether CapRelEbc can confer defense against additional types of phages beyond T7.

Additional minor points:

4. It would be helpful to note in the manuscript that the FtsZ proteins from E. coli and E. chengduensis share 97% amino acid identity, suggesting that similar interactions would also be detected in the native host. Do the differing residues have any impact on interactions?

5. Can the authors explain the apparent discrepancy between growth phenotypes in Fig. 1H (delayed growth over 2 hours) and Fig. S2A (no growth overnight) under the same induction conditions?

6. The labeling in Figures 4C-D is somewhat difficult to follow. A bar plot similar to Fig. 3B might improve clarity.

7. Are there detectable differences in the FtsZ-Gp0.4 complex with and without CapRel present?

Reviewer #2

(Remarks to the Author)
The manuscript “Activation of a bacterial immunity protein by a viral-host factor complex” by Zhang et al. investigates a form of the phage defense protein CapRel and find that activation depends on the host protein FtsZ as well as the phage protein Gp0.4. In this paper the authors set out to characterize a form of CapRel (CapRelEbc) that is distinct from the previously characterized CapRel (CapRelSJ46) system that is activated via direct binding to phage capsid. They find that this system specifically defends against the phage T7. By generating phages capable of evading defense, the authors uncover that activation relies on the T7 protein Gp0.4. Gp0.4 has previously been described as a FtsZ inhibitor therefore raising the question, does CapRel activation have anything to do with Gp0.4 inhibition of FtsZ? Using diverse known FtsZ inhibitors the authors first demonstrate that CapRel activation is specific to Gp0.4-based inhibition and not simply general FtsZ inhibition, suggesting direct protein binding is involved. A series of point mutations within Gp0.4 are used in a both bacterial growth and yeast two-hybrid assays to begin building a model in which Gp0.4 binding to FtsZ alone doesn’t trigger activation but that CapRel must also bind Gp0.4. The use of mutant forms of FtsZ and additional yeast two-hybrid assays supports a model in that Gp0.4, FtsZ, and CapRel form a ternary complex, and it is the formation of this complex that leads to activation of the toxic domain in CapRel and downstream phage defense. This is supported by an AlphaFold model which also provides an explanation for how the antitoxin domain is displaced through the binding of the FtsZ-Gp0.4 complex. Finally, the authors employ HDX-MS to support the proposed binding interface between the three proteins and how CapRel’s confirmation is changed to trigger phage defense.
The findings from this work suggest an exciting mode of immune activation in bacteria; however, the manuscript lacks overall depth and the necessary controls/experiments to fully support the conclusions.

Major Issues:
- The primary issue this work is facing is the inherent toxicity imposed by Gp0.4. While this reviewer can appreciate the complications that come with working in such system, more controls are needed. The authors phrase the toxicity of Gp0.4 as “moderately toxic” (line 108); however, this is quite misleading. If you look at the CFU assays (Fig. S2a), there is complete elimination of colony formation. Further, if you dig into the methods the complex set up of the growth assay make is clear there is more than moderate toxicity. While this does not undermine the overall findings, additional data is needed to help support the genetic/growth data. This reviewers concern is that a sick bacterium behaves quite differently than a healthy one, especially when it comes to immune signaling which is impacted by cell “health”. By saying there is “more toxicity” +/- the immune pathways could be due to numerous cell changes. Also, the likelihood that cultures are picking up mutations that elevate toxicity is high. There are a variety of different inducible system available that allow for more fine tune expression than arabinose and glucose-based systems. Can the authors use these other systems and find a level of expression where gp0.4 alone doesn’t completely kill the bacterium? These could then be used in more standard growth assays (CFU assays that don’t result in complete killing or growth curves that don’t require such extensive manipulations)? Additionally, perhaps transformation efficiency assays can be employed against instead of standard plate spot assays.
Along with this, the change in methods and presentation style of growth curves in Fig. 3 makes it hard to compare with earlier data. For examples in Fig. 3 the impact of Gp0.4 of cell growth doesn’t really manifest until later (8+ hrs) which isn’t consistent with the early time point examine in Fig. 1 and 2.
Even in the yeast two hybrid assays there is a clear impact on cell toxicity in certain conditions. Again, while this doesn’t completely undermine the findings it does complicate the findings, and this reviewer would like to see more in vitro confirmations. If this is not possible, at least in the yeast two hybrid, the catalytically dead point mutant of CapRel should be used to help reduce toxicity issues.
- As a major focus of this paper is using point mutations to infer bindings site and disentangle complex toxicity issues, the expression level of the different protein variants needs to be explored through western blot analysis. Many of these results shown could simple be due to reduced or eliminated protein expression (especially the numerous mutants tested in Gp0.4 which is so small and likely easily to disrupt).
- To help overcome the toxicity issue noted above and to help support the findings, can the authors perform more in vitro assays? The final piece of data using mass spec suggests that the authors are capable of purifying the individual components of this immune pathway (Gp0.4, FtsZ, and CapRel). The use of reciprocal IPs would help support interactions and solidify the dependency on Gp0.4 first binding FtsZ. Mutant forms of each protein could then be used in these assays to confirm in vivo findings. For example, it could be confirmed that the different point mutations in Gp0.4 truly impact the binding to one interacting partner but not the other. This could be taken one step further where the authors could show that the toxic domain of CapRel only is active in the presence of FtsZ (perhaps the monomeric mutant) and Gp0.4 using previous describe biochemical assays. This would really solidify the proposed model.
Minor Issues:
- Representation of data lacks transparency and doesn’t emphasize relevant comparisons. Throughout the manuscript the way the data is presented makes it challenging for the reading to interpret. Some examples below:
o Fig. 1A; the use of heatmap obscures the variance within biological replicates and as there is only a single defense system shown there aren’t so many comparisons that a bar graph would be overwhelming.
o The majority of phage protection is shown as plate images. While this nicely demonstrates the phenotype, a paired quantification of three biological replicates should be included. Especially in systems with toxicity, the variance between experiments needs to be shown.
o Growth curves are only two replicates and lack any statistics. When the differences are as small as in Fig. 1H, a full three replicates and stats should be shown to help support conclusions.
o There are many places where the control data should be moved to the supplement to help the reader interpret findings. Currently there is often just too much data to weed through to find the relevant comparison. For example: throughout Fig. 3; the +glucose plates can be moved to the supplement to help focus the reader on when gp0.4 is present. For the Y2H; the +His plate

can also be moved and in place labels can be places on the -His plate to make it easier to read (the current key is very confusing)
o The microscopy is really hard to see (very faint and lacks contrast) and also lacks necessary controls (no gp0.4)
o Comparing the views between Fig. 3D and Fig. 3G is impossible and this reviewer would like to see how the structure is rotated to help see where the two sets of mutations are in comparison to each other.
- With the high degree of specificity seen for this system (so specific for T7) it would be interesting to explore the conservation and distribution of the combination. Both on the side of the phage and on CapRel. The authors start to get at how CapRel proteins can recognize diverse substrates in the discussion, but can there be more? How different is CapRelEbc from CapRelSJ46? Are there more types? How different are these? Not necessary but would help but these findings in the context of the larger field. On the side of the phage, for only T7 Bas64 and Bas65 encode homologs? Are there any related ones out there?
- Why were all Gp0.4 escapee phages in the FtsZ binding site? Wouldn't it be more advantages for the phages to generate mutations like those the authors generated in Fig. 3G? As the authors state, there is a fitness advantage for phages to inhibit FtsZ, so why weren't these variants select for? At minimal this point should be discussed.
- In Fig. 1H the bottom dot in the key has a green outline but blue fill. I think it should all be green.
- Authors should consider de-emphasizing the comparison to effector triggered immunity and this model more closely resembles a form of PAMP detection. ETI really relies on the immune system monitoring an output that is changed upon infection which doesn't fit with Gp0.4.

Reviewer #3

(Remarks to the Author)
The manuscript by Zhang et al. describes a previously described tRNA pyrophosphorylase anti-phage defense system, CapRel(Ebc), and shows that it is a single-component TA system with both toxin and antioxin components. The CapRel(Ebc) protein is shown to be active against phage T7, and genetic studies show that it is activated by the presence of T7 gp0.4, an inhibitor of cell division. Additional genetic studies show that the interaction of gp0.4 with host FtsZ is required for activation of CapRel(Ebc), as point mutations in gp0.4 or FtsZ that reduce the interaction are protective. The findings are supported by site-directed mutagenesis based on predicted structures and yeast two-hybrid studies. A model is presented in which the gp0.4-FtsZ complex is required for activation of toxin activity.
The manuscript is generally well-written and well-presented, and in the absence of direct (e.g., structural) data, lays out a strong case in support of the model based on genetic experiments. Comments are noted below.

Abstract: the abstract should not contain citations.

The growth defects observed in liquid culture seem to variy between experimental systems. For example, in Fig. 1h expression of gp0.4 produces a mild growth defect but completely eliminates colony formation in Fig. 3c. How do you reconcile these differences in observation between approaches?

Fig. 1e: indicate multiple identical frameshifts were isolated.
Fig. 1f: the labeling within the genes is difficult to read. What is represented by the dashed line?
Fig. 1h: here and elsewhere, the yellow line and markers are very difficult to see, suggest use of an alternate color. Specify the meaning of EV in the caption here and elsewhere.
Line 94: Can these all be called loss of function mutations?
Fig. 2a: micrographs require scale bars. Zooming in on the cells and increasing the image contrast would help show the differences between conditions. Also specify this is phase-contrast microscopy in the figure caption.
Fig. 2d: there appears to be a plaque at the 10^-3 dilution of the wt strain expressing CapRel. Is this an anomaly or a repeatable observation?
Fig. 3i specifies means of 7-10 "plate replicates" are shown, are these true biological replicates or subsamples of the same replicate? All experiments should be performed in biological replicates.

Reviewer #4

(Remarks to the Author)
co-review

Reviewer #5

(Remarks to the Author)
Co-review

Decision Letter:

2nd September 2025

*Please ensure you delete the link to your author homepage in this e-mail if you wish to forward it to your co-authors.

Dear Mike,

Thank you for your patience while your manuscript "Activation of a bacterial immunity protein by a viral-host factor complex" was

under peer-review at Nature Microbiology. As I mentioned previously, it has now been seen by 3 referees, whose expertise and comments you will find at the end of this email. Although they find your work of considerable potential interest, they have raised a number of concerns that will need to be addressed before we can consider publication of the work in Nature Microbiology.

As you will see, all three reviewers find the work to be strong. Reviewers #1 and #3 have relatively minor concerns and suggestions, and these should be straightforward to address. Reviewer #2 raised a number of concerns regarding additional controls that they feel are necessary, as well as some additional work to deal with the toxicity issue of Gp0.4. Should further experimental data allow you to address these criticisms, we would be happy to look at a revised manuscript.

We are committed to providing a fair and constructive peer-review process. Please do not hesitate to contact us if there are specific requests from the reviewers that you believe are technically impossible or unlikely to yield a meaningful outcome.

We strongly support public availability of data. Please place the data used in your paper into a public data repository, if one exists, or alternatively, present the data as Source Data or Supplementary Information. If data can only be shared on request, please explain why in your Data Availability Statement, and also in the correspondence with your editor. For some data types, deposition in a public repository is mandatory - more information on our data deposition policies and available repositories can be found at https://www.nature.com/nature-research/editorial-policies/reporting-standards#availability-of-data.

Please include a data availability statement as a separate section after Methods but before references, under the heading "Data Availability". This section should inform readers about the availability of the data used to support the conclusions of your study. This information includes accession codes to public repositories (data banks for protein, DNA or RNA sequences, microarray, proteomics data etc…), references to source data published alongside the paper, unique identifiers such as URLs to data repository entries, or data set DOIs, and any other statement about data availability. At a minimum, you should include the following statement: "The data that support the findings of this study are available from the corresponding author upon request", mentioning any restrictions on availability. If DOIs are provided, we also strongly encourage including these in the Reference list (authors, title, publisher (repository name), identifier, year). For more guidance on how to write this section please see: http://www.nature.com/authors/policies/data/data-availability-statements-data-citations.pdf

If revising your manuscript:

* Include a "Response to referees" document detailing, point-by-point, how you addressed each referee comment. If no action was taken to address a point, you must provide a compelling argument. This response will be sent back to the referees along with the revised manuscript.

* If you have not done so already we suggest that you begin to revise your manuscript so that it conforms to our Article format instructions at http://www.nature.com/nmicrobiol/info/final-submission. Refer also to any guidelines provided in this letter.

* Include a revised version of any required reporting checklist. It will be available to referees (and, potentially, statisticians) to aid in their evaluation if the manuscript goes back for peer review. A revised checklist is essential for re-review of the paper.

When submitting the revised version of your manuscript, please pay close attention to our href="https://www.nature.com/nature-portfolio/editorial-policies/image-integrity">Digital Image Integrity Guidelines. and to the following points below:

-- that unprocessed scans are clearly labelled and match the gels and western blots presented in figures.
-- that control panels for gels and western blots are appropriately described as loading on sample processing controls
-- all images in the paper are checked for duplication of panels and for splicing of gel lanes.

EXTENDED DATA FIGURES

When re-submitting your manuscript, please ensure that any supplementary figures and tables that are crucial to the manuscript's conclusions are converted into Extended Data figures and tables to increase visibility of these data. Extended Data figures and tables are online-only (present in the online PDF and full-text HTML versions of the paper), peer-reviewed display items that provide essential background to the article but are not included in the main article due to space constraints. A maximum of ten Extended Data display items (figures and tables) is permitted.

Finally, please ensure that you retain unprocessed data and metadata files after publication, ideally archiving data in perpetuity, as these may be requested during the peer review and production process or after publication if any issues arise.

Please use the link below to submit a revised paper:

Link Redacted

Note: This url links to your confidential homepage and associated information about manuscripts you may have submitted or be reviewing for us. If you wish to forward this e-mail to co-authors, please delete this link to your homepage first.

Nature Microbiology is committed to improving transparency in authorship. As part of our efforts in this direction, we are now requesting that all authors identified as 'corresponding author' on published papers create and link their Open Researcher and Contributor Identifier (ORCID) with their account on the Manuscript Tracking System (MTS), prior to acceptance. This applies to

primary research papers only. ORCID helps the scientific community achieve unambiguous attribution of all scholarly contributions. You can create and link your ORCID from the home page of the MTS by clicking on ‘Modify my Springer Nature account’. For more information please visit please visit www.springernature.com/orcid.

If you wish to submit a suitably revised manuscript we would hope to receive it within 6 months. If you cannot send it within this time, please let us know. We will be happy to consider your revision, even if a similar study has been accepted for publication at Nature Microbiology or published elsewhere (up to a maximum of 6 months).

In the meantime we hope that you find our referees' comments helpful.

Yours sincerely,

****
Reviewer Expertise:

Referee #1: phage defense, structural biology
Referee #2: phage defense, evolution, biochemistry
Referee #3: phage defense, phage biology, genomics and genetics

Reviewer Comments:

Reviewer #1 (Remarks to the Author):

In this manuscript, Zhang et al. describe a novel activation mechanism for the bacterial defense protein CapRelEbc from Enterobacter chengduensis, showing that it requires both a phage protein and a host factor for activation. The authors demonstrate that CapRelEbc protects E. coli from T7 infection by detecting a complex between the T7 protein Gp0.4 and the bacterial cell division protein FtsZ. Gp0.4 alone does not activate CapRelEbc; instead, it binds to the synergy loop of FtsZ to block polymerization, and CapRelEbc senses this Gp0.4-FtsZ complex. A mutant FtsZ variant (ftsZ9) that resists Gp0.4 binding abolishes defense activation, and other unrelated FtsZ inhibitors, such as SulA and Kil peptides, fail to trigger CapRelEbc, indicating that FtsZ inhibition alone is insufficient.

Mechanistic dissection through genetic screens, mutational analysis, structural modeling, and HDX reveals that Gp0.4 interacts with both FtsZ and the C-terminal antitoxin domain of CapRelEbc. Yeast two-hybrid assays demonstrate that CapRelEbc and Gp0.4 do not interact in isolation but only in the presence of FtsZ, removing the bacterial genetic background and providing additional confirmation of the ternary complex model.

Overall, this work is elegant and broadens the conceptual framework of bacterial immune sensing and draws interesting parallels to eukaryotic effector-triggered immunity (ETI). The findings suggest that searches for phage defense activators should consider host factors in addition to phage proteins and highlight the rapid functional diversification of related immunity proteins.

Major comments:

1. I believe the manuscript would be strengthened by additional biochemical characterization of the ternary complex in vitro. Since the major novelty of this work is in demonstrating recognition of a phage protein-host factor complex, direct biochemical validation of that complex would reinforce the conclusions. This could include, for example, HDX experiments omitting one component, binding affinity measurements, purification of the intact complex, or in vitro reconstitution assays such as pyrophosphorylation.

2. The data clearly demonstrate the necessity of FtsZ for the recognition of Gp0.4 by CapRelEbc. However, it is not entirely clear how FtsZ becomes essential for this recognition, as the AlphaFold3 model suggests only modest direct contacts between CapRelEbc and FtsZ. Could the binding of FtsZ instead stabilize the conformation of Gp0.4, making it more favorable for interaction with CapRelEbc?

3. CapRelEbc and CapRelSJ46 are highly similar yet differ in binding specificity. Beyond the structural overlay in Fig. S5, a deeper analysis of sequence differences at the interaction interfaces would be helpful. Can the difference be rationalized by the amino acid changes between Ebc and SJ46 at the interaction residues with FtsZ, MCP, or Gp54? Could either protein recognize the other’s known phage targets, depending on the identity of the infecting phage? If not, what prevents cross-recognition despite high sequence identity? If possible, it would also be valuable to expand the phage panel to determine whether CapRelEbc can confer defense against additional types of phages beyond T7.

Additional minor points:

4. It would be helpful to note in the manuscript that the FtsZ proteins from E. coli and E. chengduensis share 97% amino acid identity, suggesting that similar interactions would also be detected in the native host. Do the differing residues have any impact on interactions?

5. Can the authors explain the apparent discrepancy between growth phenotypes in Fig. 1H (delayed growth over 2 hours) and Fig. S2A (no growth overnight) under the same induction conditions?

6. The labeling in Figures 4C-D is somewhat difficult to follow. A bar plot similar to Fig. 3B might improve clarity.

7. Are there detectable differences in the FtsZ-Gp0.4 complex with and without CapRel present?

Reviewer #2 (Remarks to the Author):

The manuscript "Activation of a bacterial immunity protein by a viral-host factor complex" by Zhang et al. investigates a form of the phage defense protein CapRel and find that activation depends on the host protein FtsZ as well as the phage protein Gp0.4. In this paper the authors set out to characterize a form of CapRel (CapRelEbc) that is distinct from the previously characterized CapRel (CapRelSJ46) system that is activated via direct binding to phage capsid. They find that this system specifically defends against the phage T7. By generating phages capable of evading defense, the authors uncover that activation relies on the T7 protein Gp0.4. Gp0.4 has previously been described as a FtsZ inhibitor therefore raising the question, does CapRel activation have anything to do with Gp0.4 inhibition of FtsZ? Using diverse known FtsZ inhibitors the authors first demonstrate that CapRel activation is specific to Gp0.4-based inhibition and not simply general FtsZ inhibition, suggesting direct protein binding is involved. A series of point mutations within Gp0.4 are used in a both bacterial growth and yeast two-hybrid assays to begin building a model in which Gp0.4 binding to FtsZ alone doesn't trigger activation but that CapRel must also bind Gp0.4. The use of mutant forms of FtsZ and additional yeast two-hybrid assays supports a model in that Gp0.4, FtsZ, and CapRel form a ternary complex, and it is the formation of this complex that leads to activation of the toxic domain in CapRel and downstream phage defense. This is supported by an AlphaFold model which also provides an explanation for how the antitoxin domain is displaced through the binding of the FtsZ-Gp0.4 complex. Finally, the authors employ HDX-MS to support the proposed binding interface between the three proteins and how CapRel's confirmation is changed to trigger phage defense.
The findings from this work suggest an exciting mode of immune activation in bacteria; however, the manuscript lacks overall depth and the necessary controls/experiments to fully support the conclusions.

Major Issues:
- The primary issue this work is facing is the inherent toxicity imposed by Gp0.4. While this reviewer can appreciate the complications that come with working in such system, more controls are needed. The authors phrase the toxicity of Gp0.4 as "moderately toxic" (line 108); however, this is quite misleading. If you look at the CFU assays (Fig. S2a), there is complete elimination of colony formation. Further, if you dig into the methods the complex set up of the growth assay make is clear there is more than moderate toxicity. While this does not undermine the overall findings, additional data is needed to help support the genetic/growth data. This reviewers concern is that a sick bacterium behaves quite differently than a healthy one, especially when it comes to immune signaling which is impacted by cell "health". By saying there is "more toxicity" +/- the immune pathways could be due to numerous cell changes. Also, the likelihood that cultures are picking up mutations that elevate toxicity is high. There are a variety of different inducible system available that allow for more fine tune expression than arabinose and glucose-based systems. Can the authors use these other systems and find a level of expression where gp0.4 alone doesn't completely kill the bacterium? These could then be used in more standard growth assays (CFU assays that don't result in complete killing or growth curves that don't require such extensive manipulations)? Additionally, perhaps transformation efficiency assays can be employed against instead of standard plate spot assays.
Along with this, the change in methods and presentation style of growth curves in Fig. 3 makes it hard to compare with earlier data. For examples in Fig. 3 the impact of Gp0.4 of cell growth doesn't really manifest until later (8+ hrs) which isn't consistent with the early time point examine in Fig. 1 and 2.
Even in the yeast two hybrid assays there is a clear impact on cell toxicity in certain conditions. Again, while this doesn't completely undermine the findings it does complicate the findings, and this reviewer would like to see more in vitro confirmations. If this is not possible, at least in the yeast two hybrid, the catalytically dead point mutant of CapRel should be used to help reduce toxicity issues.
- As a major focus of this paper is using point mutations to infer bindings site and disentangle complex toxicity issues, the expression level of the different protein variants needs to be explored through western blot analysis. Many of these results shown could simple be due to reduced or eliminated protein expression (especially the numerous mutants tested in Gp0.4 which is so small and likely easily to disrupt).
- To help overcome the toxicity issue noted above and to help support the findings, can the authors perform more in vitro assays? The final piece of data using mass spec suggests that the authors are capable of purifying the individual components of this immune pathway (Gp0.4, FtsZ, and CapRel). The use of reciprocal IPs would help support interactions and solidify the dependency on Gp0.4 first binding FtsZ. Mutant forms of each protein could then be used in these assays to confirm in vivo findings. For example, it could be confirmed that the different point mutations in Gp0.4 truly impact the binding to one interacting partner but not the other. This could be taken one step further where the authors could show that the toxic domain of CapRel only is active in the presence of FtsZ (perhaps the monomeric mutant) and Gp0.4 using previous describe biochemical assays. This would really solidify the proposed model.
Minor Issues:
- Representation of data lacks transparency and doesn't emphasize relevant comparisons. Throughout the manuscript the way the data is presented makes it challenging for the reading to interpret. Some examples below:
o Fig. 1A; the use of heatmap obscures the variance within biological replicates and as there is only a single defense system shown there aren't so many comparisons that a bar graph would be overwhelming.
o The majority of phage protection is shown as plate images. While this nicely demonstrates the phenotype, a paired quantification of three biological replicates should be included. Especially in systems with toxicity, the variance between experiments needs to be shown.
o Growth curves are only two replicates and lack any statistics. When the differences are as small as in Fig. 1H, a full three replicates and stats should be shown to help support conclusions.
o There are many places where the control data should be moved to the supplement to help the reader interpret findings. Currently there is often just too much data to weed through to find the relevant comparison. For example: throughout Fig. 3; the +glucose plates can be moved to the supplement to help focus the reader on when gp0.4 is present. For the Y2H; the +His plate

can also be moved and in place labels can be places on the -His plate to make it easier to read (the current key is very confusing)
o The microscopy is really hard to see (very faint and lacks contrast) and also lacks necessary controls (no gp0.4)
o Comparing the views between Fig. 3D and Fig. 3G is impossible and this reviewer would like to see how the structure is rotated to help see where the two sets of mutations are in comparison to each other.
- With the high degree of specificity seen for this system (so specific for T7) it would be interesting to explore the conservation and distribution of the combination. Both on the side of the phage and on CapRel. The authors start to get at how CapRel proteins can recognize diverse substrates in the discussion, but can there be more? How different is CapRelEbc from CapRelSJ46? Are there more types? How different are these? Not necessary but would help but these findings in the context of the larger field. On the side of the phage, for only T7 Bas64 and Bas65 encode homologs? Are there any related ones out there?
- Why were all Gp0.4 escapee phages in the FtsZ binding site? Wouldn't it be more advantages for the phages to generate mutations like those the authors generated in Fig. 3G? As the authors state, there is a fitness advantage for phages to inhibit FtsZ, so why weren't these variants select for? At minimal this point should be discussed.
- In Fig. 1H the bottom dot in the key has a green outline but blue fill. I think it should all be green.
- Authors should consider de-emphasizing the comparison to effector triggered immunity and this model more closely resembles a form of PAMP detection. ETI really relies on the immune system monitoring an output that is changed upon infection which doesn't fit with Gp0.4.

Reviewer #3 (Remarks to the Author):

The manuscript by Zhang et al. describes a previously described tRNA pyrophosphorylase anti-phage defense system, CapRel(Ebc), and shows that it is a single-component TA system with both toxin and antioxin components. The CapRel(Ebc) protein is shown to be active against phage T7, and genetic studies show that it is activated by the presence of T7 gp0.4, an inhibitor of cell division. Additional genetic studies show that the interaction of gp0.4 with host FtsZ is required for activation of CapRel(Ebc), as point mutations in gp0.4 or FtsZ that reduce the interaction are protective. The findings are supported by site-directed mutagenesis based on predicted structures and yeast two-hybrid studies. A model is presented in which the gp0.4-FtsZ complex is required for activation of toxin activity.
The manuscript is generally well-written and well-presented, and in the absence of direct (e.g., structural) data, lays out a strong case in support of the model based on genetic experiments. Comments are noted below.

Abstract: the abstract should not contain citations.

The growth defects observed in liquid culture seem to variy between experimental systems. For example, in Fig. 1h expression of gp0.4 produces a mild growth defect but completely eliminates colony formation in Fig. 3c. How do you reconcile these differences in observation between approaches?

Fig. 1e: indicate multiple identical frameshifts were isolated.
Fig. 1f: the labeling within the genes is difficult to read. What is represented by the dashed line?
Fig. 1h: here and elsewhere, the yellow line and markers are very difficult to see, suggest use of an alternate color. Specify the meaning of EV in the caption here and elsewhere.
Line 94: Can these all be called loss of function mutations?
Fig. 2a: micrographs require scale bars. Zooming in on the cells and increasing the image contrast would help show the differences between conditions. Also specify this is phase-contrast microscopy in the figure caption.
Fig. 2d: there appears to be a plaque at the 10^-3 dilution of the wt strain expressing CapRel. Is this an anomaly or a repeatable observation?
Fig. 3i specifies means of 7-10 "plate replicates" are shown, are these true biological replicates or subsamples of the same replicate? All experiments should be performed in biological replicates.

****

Version 1:

Reviewer comments:

Reviewer #1

(Remarks to the Author)
The authors have thoroughly and thoughtfully addressed my previous concerns. The inclusion of new in vitro biochemical data significantly strengthens the central claim of the paper that CapRelEbc activation requires a ternary complex with both Gp0.4 and FtsZ. Overall, the revised manuscript provides a compelling, well-supported mechanism for this unique defense system.

I have only one minor suggestion for the final text. Since ITC is the biochemical method used that definitively establishes the binding stoichiometry, I recommend adding a brief statement around lines 234-235 to point out the 1:1:1 molar ratio of the ternary complex derived from the ITC curves.

Congratulations to the authors on an excellent study.

Reviewer #3

(Remarks to the Author)
I thank the authors for their revisions to the manuscript, including additional text and modifications to figures. I have no additional revisions.

Reviewer #4

(Remarks to the Author)
Co-review

Reviewer #5

(Remarks to the Author)

Decision Letter:

26th March 2026

*Please ensure you delete the link to your author homepage in this e-mail if you wish to forward it to your co-authors.

Dear Professor Laub,

Thank you for your patience while your manuscript "Activation of a bacterial immunity protein by a viral-host factor complex" was under peer-review at Nature Microbiology. . It has now been seen by 2 referees, whose expertise and comments you will find at the end of this email. Unfortunately referee #2 was unable to look again but we asked reviewer #1 to assess your responses to referee #2's concerns.

The referees are now happy and we will be proceeding with your manuscript. Before I send the official accept in principle letter, I need you to convert the supplementary figures to extended data (maximum of 10 extended data figures are allowed) to enable our editorial checks. See below for further details.

EXTENDED DATA FIGURES

When re-submitting your manuscript, please ensure that any supplementary figures and tables that are crucial to the manuscript's conclusions are converted into Extended Data figures and tables to increase visibility of these data. Extended Data figures and tables are online-only (present in the online PDF and full-text HTML versions of the paper), peer-reviewed display items that provide essential background to the article but are not included in the main article due to space constraints. A maximum of ten Extended Data display items (figures and tables) is permitted.

Finally, please ensure that you retain unprocessed data and metadata files after publication, ideally archiving data in perpetuity, as these may be requested during the peer review and production process or after publication if any issues arise.

Please resubmit electronically using the link below to access your home page:

Link Redacted

*This url links to your confidential homepage and associated information about manuscripts you may have submitted or be reviewing for us. If you wish to forward this e-mail to co-authors, please delete this link to your homepage first.

Please ensure that all correspondence is marked with your Nature Microbiology reference number in the subject line.

Once these changes have been made, we will proceed with your manuscript.

We look forward to hearing from you soon.

Yours sincerely,

****

Reviewers Comments:

Reviewer #1 (Remarks to the Author):

The authors have thoroughly and thoughtfully addressed my previous concerns. The inclusion of new in vitro biochemical data significantly strengthens the central claim of the paper that CapRelEbc activation requires a ternary complex with both Gp0.4 and FtsZ. Overall, the revised manuscript provides a compelling, well-supported mechanism for this unique defense system.

I have only one minor suggestion for the final text. Since ITC is the biochemical method used that definitively establishes the binding stoichiometry, I recommend adding a brief statement around lines 234-235 to point out the 1:1:1 molar ratio of the ternary complex derived from the ITC curves.

Congratulations to the authors on an excellent study.

Reviewer #3 (Remarks to the Author):

I thank the authors for their revisions to the manuscript, including additional text and modifications to figures. I have no additional revisions.

****

Version 2:

Decision Letter:

Our ref: NMICROBIOL-25062352B

1st April 2026

Dear Dr. Laub,

Thank you for submitting your revised manuscript "Activation of a bacterial immunity protein by a viral-host factor complex" (NMICROBIOL-25062352B). It has now been seen by the original referees and their comments are below. The reviewers find that the paper has improved in revision, and therefore we'll be happy in principle to publish it in Nature Microbiology, pending minor revisions to satisfy the referees' final requests and to comply with our editorial and formatting guidelines.

If the current version of your manuscript is in a PDF format, please email us a copy of the file in an editable format (Microsoft Word or LaTex)-- we can not proceed with PDFs at this stage.

We are now performing detailed checks on your paper and will send you a checklist detailing our editorial and formatting requirements in about a week. Please do not upload the final materials and make any revisions until you receive this additional information from us.

Thank you again for your interest in Nature Microbiology Please do not hesitate to contact me if you have any questions.

Sincerely,

Version 3:

Decision Letter:

12th May 2026

Dear Mike,

Great to catch up in person last week at the SISB Conference! Some good news:

I am pleased to accept your Article "Bacterial cell division protein FtsZ complexes with a phage protein to activate bacterial

immunity" for publication in Nature Microbiology. Thank you for having chosen to submit your work to us and many congratulations.

Over the next few weeks, your paper will be copyedited to ensure that it conforms to Nature Microbiology style. We look particularly carefully at the titles of all papers to ensure that they are relatively brief and understandable.

Once your paper is typeset, you will receive an email with a link to choose the appropriate publishing options for your paper and our Author Services team will be in touch regarding any additional information that may be required. Once your paper has been scheduled for online publication, the Nature press office will be in touch to confirm the details.

You may wish to make your media relations office aware of your accepted publication, in case they consider it appropriate to organize some internal or external publicity. Once your paper has been scheduled you will receive an email confirming the publication details. This is normally 3-4 working days in advance of publication. If you need additional notice of the date and time of publication, please let the production team know when you receive the proof of your article to ensure there is sufficient time to coordinate. Further information on our embargo policies can be found here:
https://www.nature.com/authors/policies/embargo.html

After the grant of rights is completed, you will receive a link to your electronic proof via email with a request to make any corrections within 48 hours. If, when you receive your proof, you cannot meet this deadline, please inform us at rjsproduction@springernature.com immediately. You will not receive your proofs until the publishing agreement has been received through our system

Due to the importance of these deadlines, we ask you please us know now whether you will be difficult to contact over the next month. If this is the case, we ask you provide us with the contact information (email, phone and fax) of someone who will be able to check the proofs on your behalf, and who will be available to address any last-minute problems.

Acceptance of your manuscript is conditional on all authors' agreement with our publication policies (see https://www.nature.com/nmicrobiol/editorial-policies). In particular your manuscript must not be published elsewhere.

Authors may need to take specific actions to achieve compliance with funder and institutional open access mandates. If your research is supported by a funder that requires immediate open access (e.g. according to Plan S principles or the NIH public access policy) then you should select the gold OA route, and we will direct you to the compliant route where possible. Because authors warrant under our subscription licensing terms that they haven’t committed to licensing any version of their article under a licence inconsistent with the terms of our agreement – including the applicable embargo period – publication under the subscription model isn’t suitable for authors whose funders require no embargo.

If you have any questions about our publishing options, costs, Open Access requirements, or our legal forms, please contact ASJournals@springernature.com

An online order form for reprints of your paper is available at https://www.nature.com/reprints/author-reprints.html. All co-authors, authors' institutions and authors' funding agencies can order reprints using the form appropriate to their geographical region.

We welcome the submission of potential cover material (including a short caption of around 40 words) related to your manuscript; suggestions should be sent to Nature Microbiology as electronic files (the image should be 300 dpi at 210 x 297 mm in either TIFF or JPEG format). Please note that such pictures should be selected more for their aesthetic appeal than for their scientific content, and that colour images work better than black and white or grayscale images. Please do not try to design a cover with the Nature Microbiology logo etc., and please do not submit composites of images related to your work. I am sure you will understand that we cannot make any promise as to whether any of your suggestions might be selected for the cover of the journal.

You can now use a single sign-on for all your accounts, view the status of all your manuscript submissions and reviews, access usage statistics for your published articles and download a record of your refereeing activity for the Nature journals.

To assist our authors in disseminating their research to the broader community, our SharedIt initiative provides you with a unique shareable link that will allow anyone (with or without a subscription) to read the published article. Recipients of the link with a subscription will also be able to download and print the PDF.

As soon as your article is published, you will receive an automated email with your shareable link.

With kind regards,

P.S. Click on the following link if you would like to recommend Nature Microbiology to your librarian

http://www.nature.com/subscriptions/recommend.html#forms

** Visit the Springer Nature Editorial and Publishing website at www.springernature.com/editorial-and-publishing-jobs for more information about our career opportunities. If you have any questions please click here.**

Open Access This Peer Review File is licensed under a Creative Commons Attribution 4.0 International License, which permits use, sharing, adaptation, distribution and reproduction in any medium or format, as long as you give appropriate credit to the original author(s) and the source, provide a link to the Creative Commons license, and indicate if changes were made.
In cases where reviewers are anonymous, credit should be given to 'Anonymous Referee' and the source.
The images or other third party material in this Peer Review File are included in the article's Creative Commons license, unless indicated otherwise in a credit line to the material. If material is not included in the article's Creative Commons license and your intended use is not permitted by statutory regulation or exceeds the permitted use, you will need to obtain permission directly from the copyright holder.
To view a copy of this license, visit https://creativecommons.org/licenses/by/4.0/

We thank all three reviewers for their constructive comments and questions. Below we respond to each query and indicate, when appropriate, how the text and figures have been updated.

Reviewer #1 (Remarks to the Author):

In this manuscript, Zhang et al. describe a novel activation mechanism for the bacterial defense protein CapRelEbc from Enterobacter chengduensis, showing that it requires both a phage protein and a host factor for activation. The authors demonstrate that CapRelEbc protects E. coli from T7 infection by detecting a complex between the T7 protein Gp0.4 and the bacterial cell division protein FtsZ. Gp0.4 alone does not activate CapRelEbc; instead, it binds to the synergy loop of FtsZ to block polymerization, and CapRelEbc senses this Gp0.4-FtsZ complex. A mutant FtsZ variant (ftsZ9) that resists Gp0.4 binding abolishes defense activation, and other unrelated FtsZ inhibitors, such as SulA and Kil peptides, fail to trigger CapRelEbc, indicating that FtsZ inhibition alone is insufficient.

Mechanistic dissection through genetic screens, mutational analysis, structural modeling, and HDX reveals that Gp0.4 interacts with both FtsZ and the C-terminal antitoxin domain of CapRelEbc. Yeast two-hybrid assays demonstrate that CapRelEbc and Gp0.4 do not interact in isolation but only in the presence of FtsZ, removing the bacterial genetic background and providing additional confirmation of the ternary complex model.

Overall, this work is elegant and broadens the conceptual framework of bacterial immune sensing and draws interesting parallels to eukaryotic effector-triggered immunity (ETI). The findings suggest that searches for phage defense activators should consider host factors in addition to phage proteins and highlight the rapid functional diversification of related immunity proteins.

Major comments:

1. I believe the manuscript would be strengthened by additional biochemical characterization of the ternary complex in vitro. Since the major novelty of this work is in demonstrating recognition of a phage protein-host factor complex, direct biochemical validation of that complex would reinforce the conclusions. This could include, for example, HDX experiments omitting one component, binding affinity measurements, purification of the intact complex, or in vitro reconstitution assays such as pyrophosphorylation.

We have now added the following additional biochemical characterizations to show that Gp0.4 together with FtsZ can activate CapRel^{Ebc} *in vitro*.

1) Using purified CapRel^{Ebc}, FtsZ(L178E) (a monomeric variant of FtsZ), and Gp0.4 in a reconstituted transcription-translation system, we observed an inhibition of the production of a model protein DHFR only when all three proteins were present; omitting any one of the proteins abolished such inhibition (Fig. 4B). Further, no protein synthesis inhibition was observed using the L35A variant of Gp0.4 (L35 was mutated in our escape phages) (Fig. S5C).

2) We have performed ITC with purified proteins, and confirmed an interaction between FtsZ(L178E) and Gp0.4 (Fig. S3G). Importantly, we also observed interaction between CapRel^{Ebc} and the complex of FtsZ(L178E)-Gp0.4 by ITC (Fig. 4F), supporting the formation of a ternary complex.

These additional biochemical results, combined with our *in vivo* characterization, strongly support our model that Gp0.4 and FtsZ activate CapRel^{Ebc} via a ternary complex.

2. The data clearly demonstrate the necessity of FtsZ for the recognition of Gp0.4 by CapRelEbc. However, it is not entirely clear how FtsZ becomes essential for this recognition, as the AlphaFold3 model suggests only modest direct contacts between CapRelEbc and FtsZ. Could the binding of FtsZ instead stabilize the conformation of Gp0.4, making it more favorable for interaction with CapRelEbc?

We thank the reviewer for this suggestion. We think that FtsZ likely stabilizes the helical structures of Gp0.4, as has been seen with other peptide inhibitors of FtsZ such as MciZ (Bisson-Filho et al., 2015). We have now added additional data to support this idea: we show that Gp0.4 was destabilized (and therefore degraded) in the suppressor strain containing *ftsZ9* where Gp0.4 presumably no longer interacted with FtsZ (new Fig. 4H). This destabilization/degradation can be rescued by co-producing a monomeric FtsZ that interacts with Gp0.4 (Fig. 4H). Additionally, Gp0.4 variants that were unable to interact with FtsZ were found to be destabilized in cells as well (Fig. 3D, S4C). We now present and discuss these new analyses on lines 183-185 and lines 243-249 of the revised manuscript.

3. CapRelEbc and CapRelSJ46 are highly similar yet differ in binding specificity. Beyond the structural overlay in Fig. S5, a deeper analysis of sequence differences at the interaction interfaces would be helpful. Can the difference be rationalized by the amino acid changes between Ebc and SJ46 at the interaction residues with FtsZ, MCP, or Gp54? Could either protein recognize the other's known phage targets, depending on the identity of the infecting phage? If not, what prevents cross-recognition despite high sequence identity? If possible, it would also be valuable to expand the phage panel to determine whether CapRelEbc can confer defense against additional types of phages beyond T7.

We agree that it is very striking that these two highly similar proteins have different specificity with respect to phage recognition. We have previously shown that CapRel^{Ebc} cannot bind to either the MCP or Gp54 and is therefore unable to defend against these phages. We made chimeras of CapRel^{Ebc} and CapRel^{SJ46} by swapping their antitoxin regions, and found that the chimeric proteins switched their phage defense profiles, supporting the conclusion that the C-terminal antitoxin region involving the pseudo-ZFD functions as an infection sensor (Zhang et al., 2022; Zhang et al., 2024). We have now added a sequence alignment of CapRel^{Ebc} and CapRel^{SJ46} (Fig. S7), and commented that the antitoxin region is more different between these two proteins and the least conserved across diverse CapRel homologs (see lines 258-261). Pinpointing the exact subset of residues responsible for the different specificities of these homologs is, in our opinion, beyond the scope of this manuscript, but certainly an interesting angle to pursue in the future in the context of considering how phage recognition evolves.

Additional minor points:

4. It would be helpful to note in the manuscript that the FtsZ proteins from E. coli and E. chengduensis share 97% amino acid identity, suggesting that similar interactions would also be detected in the native host. Do the differing residues have any impact on interactions?

We thank the reviewer for pointing this out. Residues that are important for interacting with Gp0.4 (such as the synergy loop) are the same in these two proteins, so the interaction likely still holds in the native host. We now discuss this on lines 299-301.

5. Can the authors explain the apparent discrepancy between growth phenotypes in Fig. 1H (delayed growth over 2 hours) and Fig. S2A (no growth overnight) under the same induction conditions?

As mentioned below in response to reviewer #2 as well below: the OD_{600} measurements in Fig. 1H are, of course, a short-term assay that measures initial growth of the culture, with OD_{600} reflecting total cellular mass (not directly CFUs), whereas the spotting assay in Fig. S2A is an end-point assay after prolonged incubation and measures CFUs. Given that the effect of Gp0.4 is to inhibit cell division, its production initially leads to continued growth but substantial cell elongation (as shown by the microscopy in Figure 2A), hence the continued gain in OD_{600}; the extreme elongation, however, produces cells that are not viable in the long-term and so cannot form CFUs upon plating. This same phenomenon of continued growth in the short-term but with very limited plating viability in the long-term is also seen with other FtsZ inhibitors such as SulA, MinC, Era, and others (Gottesman et al *J Bact* 1981, Pichoff and Lutkenhaus *J Bact* 2001, Zhou et al *J Bact* 2020, Sanchez-Gorostiaga et al *mSphere* 2016). We provide additional data to support this hypothesis (see below in our response to reviewer #2) by comparing measured CFUs and calculated CFUs based on OD_{600}. We have also now commented on this issue in the text on lines 124-128.

6. The labeling in Figures 4C-D is somewhat difficult to follow. A bar plot similar to Fig. 3B might improve clarity.

We have improved the clarity of these figures (now as Fig. 4D-E) by putting labels directly next to the plates, and moved the +His plates to the supplement as suggested by reviewer #2.

7. Are there detectable differences in the FtsZ-Gp0.4 complex with and without CapRel present?

We did not see many differences in the FtsZ-Gp0.4 complex with and without $CapRel^{Ebc}$, which supports the hypothesis noted above that binding to FtsZ stabilizes Gp0.4. With the added experimental data, we now comment on this on lines 243-249.

Reviewer #2 (Remarks to the Author):

The manuscript “Activation of a bacterial immunity protein by a viral-host factor complex”

by Zhang et al. investigates a form of the phage defense protein CapRel and find that activation depends on the host protein FtsZ as well as the phage protein Gp0.4. In this paper the authors set out to characterize a form of CapRel (CapRelEbc) that is distinct from the previously characterized CapRel (CapRelSJ46) system that is activated via direct binding to phage capsid. They find that this system specifically defends against the phage T7. By generating phages capable of evading defense, the authors uncover that activation relies on the T7 protein Gp0.4. Gp0.4 has previously been described as a FtsZ inhibitor therefore raising the question, does CapRel activation have anything to do with Gp0.4 inhibition of FtsZ? Using diverse known FtsZ inhibitors the authors first demonstrate that CapRel activation is specific to Gp0.4-based inhibition and not simply general FtsZ inhibition, suggesting direct protein binding is involved. A series of point mutations within Gp0.4 are used in a both bacterial growth and yeast two-hybrid assays to begin building a model in which Gp0.4 binding to FtsZ alone doesn't trigger activation but that CapRel must also bind Gp0.4. The use of mutant forms of FtsZ and additional yeast two-hybrid assays supports a model in that Gp0.4, FtsZ, and CapRel form a ternary complex, and it is the formation of this complex that leads to activation of the toxic domain in CapRel and downstream phage defense. This is supported by an AlphaFold model which also provides an explanation for how the antitoxin domain is displaced through the binding of the FtsZ-Gp0.4 complex. Finally, the authors employ HDX-MS to support the proposed binding interface between the three proteins and how CapRel's confirmation is changed to trigger phage defense.
The findings from this work suggest an exciting mode of immune activation in bacteria; however, the manuscript lacks overall depth and the necessary controls/experiments to fully support the conclusions.

Major Issues:
- The primary issue this work is facing is the inherent toxicity imposed by Gp0.4. While this reviewer can appreciate the complications that come with working in such system, more controls are needed. The authors phrase the toxicity of Gp0.4 as "moderately toxic" (line 108); however, this is quite misleading. If you look at the CFU assays (Fig. S2a), there is complete elimination of colony formation. Further, if you dig into the methods the complex set up of the growth assay make is clear there is more than moderate toxicity. While this does not undermine the overall findings, additional data is needed to help support the genetic/growth data. This reviewers concern is that a sick bacterium behaves quite differently than a healthy one, especially when it comes to immune signaling which is impacted by cell "health". By saying there is "more toxicity" +/- the immune pathways could be due to numerous cell changes. Also, the likelihood that cultures are picking up mutations that elevate toxicity is high. There are a variety of different inducible system available that allow for more fine tune expression than arabinose and glucose-based systems. Can the authors use these other systems and find a level of expression where gp0.4 alone doesn't completely kill the bacterium? These could then be used in more standard growth assays (CFU assays that don't result in complete killing or growth curves that don't require such extensive manipulations)? Additionally, perhaps transformation efficiency assays can be employed against instead of standard plate spot assays.

Along with this, the change in methods and presentation style of growth curves in Fig. 3 makes it hard to compare with earlier data. For examples in Fig. 3 the impact of Gp0.4 of cell growth doesn't really manifest until later (8+ hrs) which isn't consistent with the early time point examine in Fig. 1 and 2.

As described in response to reviewer #1's minor comment on this same topic: The OD_{600} measurements in Fig. 1H reflect the short-term effects on cell mass accumulation, whereas the spotting/viability assay in Fig. S2A is an end-point assay after prolonged incubation and measures CFUs. Because Gp0.4 inhibits cell division, this leads to cell elongation, but continued growth in the short-term (as further shown by microscopy data in Fig. 2A); after longer periods of time, the extremely long cells have not divided to create new cells, hence the drop in viability in Fig. S2A. This same phenomenon is seen with other FtsZ/cell division inhibitors, as noted/referenced above.

To further validate our model for Gp0.4 and the consequences of its induction, we measured CFUs of cultures at 90 and 120 min post-induction (as in Fig. 1H) by plating cells on media containing glucose (see panel A below). We also plated the cells on arabinose-containing plates to verify there were no suppressors in the cultures. We measured CFUs in three independent experiments, and compared to the calculated CFUs based on the OD_{600} of the culture (see panel B below). There is a difference between the actual and calculated CFUs only in cells producing Gp0.4 (+ara), supporting the conclusion that OD_{600} measurements do not correctly reflect CFUs, and instead that OD_{600} increases because cells grow and elongate due to cell division inhibition by Gp0.4.

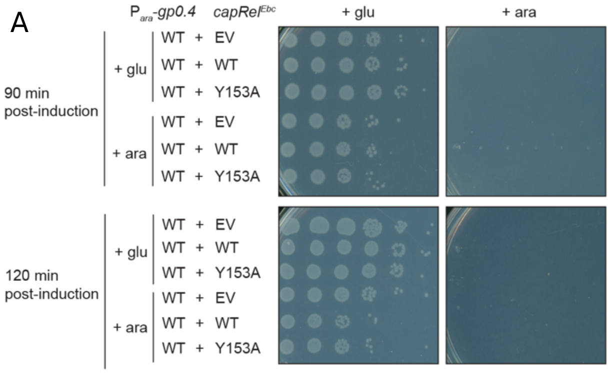

B

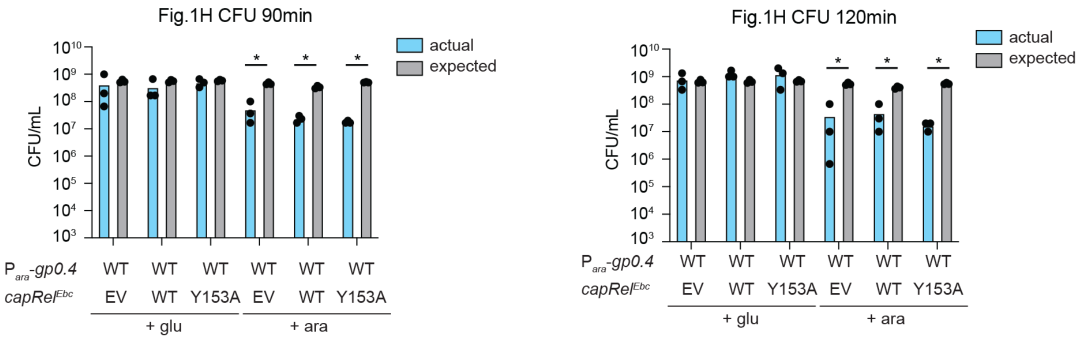

We appreciate the reviewer suggesting different induction methods (such as titrating down levels of arabinose in a relevant strain) to avoid toxicity. But the key finding of our work is that Gp0.4 must interact with FtsZ to activate CapRel^{Ebc} meaning it is not possible to separate the toxicity of wild-type Gp0.4 that results from FtsZ inhibition from the activation of CapRel^{Ebc}. In other words, if we titrate Gp0.4 levels down to a point where it no longer has any toxicity on its own, that means it's at a level where FtsZ isn't being bound, which means that it also won't activate CapRel^{Ebc}!

Further, we would emphasize that we had demonstrated that the additional toxicity associated with Gp0.4 induction required the enzymatic activity of CapRel^{Ebc}, as no toxicity was seen when co-producing Gp0.4 with the active site mutant CapRel^{Ebc} (Y153A) (Fig. 1H). And, perhaps most importantly: we showed that not all cell division inhibitors that lead to a 'sick cell' lead to additional toxicity when co-produced with CapRel^{Ebc} (Fig. 2E-G). These are extremely important controls that demonstrate that the activation is specific to Gp0.4 and not the general consequence of producing any cell division inhibitor.

As to the differences in Fig. 3 compared to Fig.1 and 2, this is because the data in Fig. 3 resulted from measurements made in a plate reader in a 96-well format starting from stationary phase cells, whereas data in Fig. 1 and 2 were from measurements made in shaking flasks starting from mid-exponential phase cells. To verify that the results are comparable, we measured growth of the Gp0.4 variants in Fig. 3 in the shaking flask set up (see data in new Fig. S4F-H).

Even in the yeast two hybrid assays there is a clear impact on cell toxicity in certain conditions. Again, while this doesn't completely undermine the findings it does complicate the findings, and this reviewer would like to see more in vitro confirmations. If this is not possible, at least in the yeast two hybrid, the catalytically dead point mutant of CapRel should be used to help reduce toxicity issues.

Neither Gp0.4 nor CapRel^{Ebc} was toxic to yeast, and we did not observe any toxicity in the yeast two hybrid assays, i.e. on non-selective media, there are no major differences in growth (non-selective conditions are in Fig. S4E and S5D-E of the revised figures). As mentioned in response to reviewer 1 and further articulated below in response to this reviewer's last 'Major' comment, we now also provide additional *in vitro* biochemical

experiments to confirm the formation of a ternary complex and the activation of CapRel^{Ebc} by Gp0.4 and FtsZ.

- As a major focus of this paper is using point mutations to infer bindings site and disentangle complex toxicity issues, the expression level of the different protein variants needs to be explored through western blot analysis. Many of these results shown could simple be due to reduced or eliminated protein expression (especially the numerous mutants tested in Gp0.4 which is so small and likely easily to disrupt).

We thank the reviewer for suggesting this. For the Gp0.4 variants in Fig. 3H, we infer that they are not simply unfolded and degraded as they can still interact with and inhibit FtsZ. For the variants in Fig. 3E-F, we tested the expression levels of these proteins and found that these Gp0.4 variants do not accumulate to the same levels as wild-type Gp0.4 (Fig. S4C), likely because an interaction with FtsZ is important for stabilizing the helical structure of Gp0.4; a similar phenomenon has been seen with other peptide inhibitors of FtsZ such as MciZ (Bisson-Filho et al., 2015). We have also now added additional data to support this idea: we show that Gp0.4 was destabilized (likely degraded) in the suppressor strain containing *ftsZ9* where Gp0.4 presumably no longer interacts with FtsZ (new Fig. 4H). The reduction in Gp0.4 levels can be rescued when co-producing monomeric FtsZ that can interact with Gp0.4 (Fig. 4H). We have now modified the text to reflect these new data (see lines 183-187, 243-249).

- To help overcome the toxicity issue noted above and to help support the findings, can the authors perform more in vitro assays? The final piece of data using mass spec suggests that the authors are capable of purifying the individual components of this immune pathway (Gp0.4, FtsZ, and CapRel). The use of reciprocal IPs would help support interactions and solidify the dependency on Gp0.4 first binding FtsZ. Mutant forms of each protein could then be used in these assays to confirm in vivo findings. For example, it could be confirmed that the different point mutations in Gp0.4 truly impact the binding to one interacting partner but not the other. This could be taken one step further where the authors could show that the toxic domain of CapRel only is active in the presence of FtsZ (perhaps the monomeric mutant) and Gp0.4 using previous describe biochemical assays. This would really solidify the proposed model.

As noted in response to reviewer #1, we have now added the following additional biochemical data to show that Gp0.4 together with FtsZ can activate CapRel^{Ebc} *in vitro*, which further supports our proposed model.

1) We incubated purified CapRel^{Ebc}, FtsZ(L178E) (a monomeric variant of FtsZ), and Gp0.4 in a reconstituted transcription-translation system, and observed inhibited production of a model protein DHFR only when all three proteins were present; omitting any single protein abolished such inhibition (Fig. 4B). No protein synthesis inhibition was observed using the L35A variant of Gp0.4 (L35 was mutated in our escape phages) (Fig. S5C).

2) We have performed ITC with purified proteins, and confirmed an interaction between FtsZ(L178E) and Gp0.4 (Fig. S3G). Importantly, we also observed interaction between

CapRel^{Ebc} and the complex of FtsZ(L178E)-Gp0.4 by ITC (Fig. 4F), supporting the formation of a ternary complex.

These additional biochemical results, combined with our *in vivo* studies, strongly support our model that Gp0.4 and FtsZ activate CapRel^{Ebc} via a ternary complex.

Minor Issues:
- Representation of data lacks transparency and doesn't emphasize relevant comparisons. Throughout the manuscript the way the data is presented makes it challenging for the reading to interpret. Some examples below:

o Fig. 1A; the use of heatmap obscures the variance within biological replicates and as there is only a single defense system shown there aren't so many comparisons that a bar graph would be overwhelming.

As suggested, bar graphs summarizing 3 independent biological replicates are now included in Fig. S1B.

o The majority of phage protection is shown as plate images. While this nicely demonstrates the phenotype, a paired quantification of three biological replicates should be included. Especially in systems with toxicity, the variance between experiments needs to be shown.

We prefer showing raw plate images for phage protection assays as it shows both the PFU and plaque sizes, but as suggested, we now also included bar graphs quantifying each of the spotting assays with 3 biological replicates (Fig. S1B, S1C, S1E, S1H, S2B).

o Growth curves are only two replicates and lack any statistics. When the differences are as small as in Fig. 1H, a full three replicates and stats should be shown to help support conclusions.

We have now included 3 biological replicates for growth curves shown in Fig. 1H, 2C, 2E, 2F, 2G, and statistics have been added for Fig. 1H where a difference was seen. The additional repeats have not changed any of the original conclusions.

o There are many places where the control data should be moved to the supplement to help the reader interpret findings. Currently there is often just too much data to weed through to find the relevant comparison. For example: throughout Fig. 3; the +glucose plates can be moved to the supplement to help focus the reader on when gp0.4 is present. For the Y2H; the +His plate can also be moved and in place labels can be places on the -His plate to make it easier to read (the current key is very confusing)

As suggested, the +glucose plates in Fig. 3 and the +His plates of Y2H in Fig. 4 have been moved to the supplement. We now place labels directly on the -His plates to improve clarify as suggested.

o The microscopy is really hard to see (very faint and lacks contrast) and also lacks necessary controls (no gp0.4)

We have improved the contrast of the microscopy images and included scale bars. Since Gp0.4 is under arabinose-inducible promoter and otherwise suppressed with glucose, the t = 0 min is the control for cells without Gp0.4 expression.

o Comparing the views between Fig. 3D and Fig. 3G is impossible and this reviewer would like to see how the structure is rotated to help see where the two sets of mutations are in comparison to each other.

We have now added a symbol indicating the relative rotation of Fig. 3D and Fig. 3G.

- With the high degree of specificity seen for this system (so specific for T7) it would be interesting to explore the conservation and distribution of the combination. Both on the side of the phage and on CapRel. The authors start to get at how CapRel proteins can recognize diverse substrates in the discussion, but can there be more? How different is CapRelEbc from CapRelSJ46? Are there more types? How different are these? Not necessary but would help but these findings in the context of the larger field. On the side of the phage, for only T7 Bas64 and Bas65 encode homologs? Are there any related ones out there?

As noted above in response to reviewer #1, we have previously shown that CapRel^{Ebc} cannot bind to either the MCP or Gp54 that activate CapRel^{SJ46}, and so is unable to defend against the phages encoding those activators. We made chimeras of CapRel^{Ebc} and CapRel^{SJ46} by swapping their antitoxin regions, and found that the chimeric proteins switched their phage defense profile, supporting the conclusion that the C-terminal antitoxin involving the pseudo-ZFD functions as an infection sensor (Zhang et al., 2022; Zhang et al., 2024). We have now added a sequence alignment of CapRel^{Ebc} and CapRel^{SJ46} (Fig. S7), and commented that the antitoxin region is more different between these two proteins and is the least conserved region across diverse CapRel homologs (see lines 258-261).

We did not find any other phages that encode Gp0.4 homologs, but it is possible that CapRel^{Ebc} can sense other phage-encoded triggers that are not related to Gp0.4, as seen with CapRel^{SJ46}, which can bind two unrelated proteins (the MCP and Gp54).

- Why were all Gp0.4 escapee phages in the FtsZ binding site? Wouldn't it be more advantages for the phages to generate mutations like those the authors generated in Fig. 3G? As the authors state, there is a fitness advantage for phages to inhibit FtsZ, so why weren't these variants select for? At minimal this point should be discussed.

In addition to the mutants that map to the FtsZ binding site, we also isolated phage escapers that have a frameshift in Gp0.4 (Fig. 1E, S1F). Why we did not get mutations like those in Fig. 3G is not clear. Obviously our escape screen was not done to saturation so other mutations may have arisen if we continued screening. Whether there is a significant enough fitness disadvantage in our growth and plaquing conditions following loss of Gp0.4 function is not clear. There are also potentially other trade-offs introduced by various mutations that influence their frequencies, as well as possible non-uniformities in

mutation rates across phage genes. Given so much uncertainty about why our limited escape screen only produced two substitutions mapping to the FtsZ binding surface and a frameshift, we are hesitant to speculate on this topic in the manuscript.

- In Fig. 1H the bottom dot in the key has a green outline but blue fill. I think it should all be green.

We thank the reviewer for pointing this out - this has now been fixed.

- Authors should consider de-emphasizing the comparison to effector triggered immunity and this model more closely resembles a form of PAMP detection. ETI really relies on the immune system monitoring an output that is changed upon infection which doesn't fit with Gp0.4.

We have removed one case in the Discussion where we agree too strong a comparison was being made, but we retain some of the general commentary on this point. We think the commentary is explicit about the fact that Gp0.4 does not perfectly adhere to the ETI framework. For instance, on lines 317-320 we note that "Our studies indicate that the activation of $CapRel^{Ebc}$ shares features of both PAMP and ETI-based strategies, binding to the T7 phage protein Gp0.4, but only once this phage-encoded effector has bound to monomeric FtsZ to inhibit cell division" and later on lines 326-328 that "Although akin to ETI in some ways, $CapRel^{Ebc}$ does not solely sense the consequences of cell division inhibition by Gp0.4 as evidenced by our finding that other FtsZ inhibitors, such as SulA and the Kil peptides, do not trigger $CapRel^{Ebc}$ activity." We think the comparing and contrasting to eukaryotic concepts is useful with the clear differences being explicitly discussed.

Reviewer #3 (Remarks to the Author):

The manuscript by Zhang et al. describes a previously described tRNA pyrophosphorylase anti-phage defense system, CapRel(Ebc), and shows that it is a single-component TA system with both toxin and antioxin components. The CapRel(Ebc) protein is shown to be active against phage T7, and genetic studies show that it is activated by the presence of T7 gp0.4, an inhibitor of cell division. Additional genetic studies show that the interaction of gp0.4 with host FtsZ is required for activation of CapRel(Ebc), as point mutations in gp0.4 or FtsZ that reduce the interaction are protective. The findings are supported by site-directed mutagenesis based on predicted structures and yeast two-hybrid studies. A model is presented in which the gp0.4-FtsZ complex is required for activation of toxin activity.

The manuscript is generally well-written and well-presented, and in the absence of direct (e.g., structural) data, lays out a strong case in support of the model based on genetic experiments. Comments are noted below.

Abstract: the abstract should not contain citations.

Citations have been removed from the abstract.

The growth defects observed in liquid culture seem to variy between experimental systems. For example, in Fig. 1h expression of gp0.4 produces a mild growth defect but completely eliminates colony formation in Fig. 3c. How do you reconcile these differences in observation between approaches?

Please see our responses to reviewer 1 (minor point #5) and reviewer 2 (point #1) on this same issue.

Fig. 1e: indicate multiple identical frameshifts were isolated.

We have now clarified the frameshifts were identical in lines 91-92.

Fig. 1f: the labeling within the genes is difficult to read. What is represented by the dashed line?

We have improved the clarity of Fig. 1F by using a different approach, which now highlights the homologous genes across these phages.

Fig. 1h: here and elsewhere, the yellow line and markers are very difficult to see, suggest use of an alternate color. Specify the meaning of EV in the caption here and elsewhere.

We have changed the yellow to orange line across the paper, and specified the meaning of EV.

Line 94: Can these all be called loss of function mutations?

As suggested, we have removed the 'loss of function'.

Fig. 2a: micrographs require scale bars. Zooming in on the cells and increasing the image contrast would help show the differences between conditions. Also specify this is phase-contrast microscopy in the figure caption.

Scale bars have been added, and the image contrast has been adjusted. We also now specify it is phage-contrast microscopy.

Fig. 2d: there appears to be a plaque at the 10^-3 dilution of the wt strain expressing CapRel. Is this an anomaly or a repeatable observation?

We believe this is an anomaly, and we have now replaced the figure with a different experiment. All spotting assays have been done 3 times independently, and we now added quantification in Fig. S2B.

Fig. 3i specifies means of 7-10 "plate replicates" are shown, are these true biological replicates or subsamples of the same replicate? All experiments should be performed in biological replicates.

They are plate replicates from the same culture, but the entire experiments have been repeated at least twice. We have now clarified this in the caption.

We thank all three reviewers for their constructive comments and questions. Below we respond to each query and indicate, when appropriate, how the text and figures have been updated.

Reviewer #1 (Remarks to the Author):

In this manuscript, Zhang et al. describe a novel activation mechanism for the bacterial defense protein CapRelEbc from Enterobacter chengduensis, showing that it requires both a phage protein and a host factor for activation. The authors demonstrate that CapRelEbc protects E. coli from T7 infection by detecting a complex between the T7 protein Gp0.4 and the bacterial cell division protein FtsZ. Gp0.4 alone does not activate CapRelEbc; instead, it binds to the synergy loop of FtsZ to block polymerization, and CapRelEbc senses this Gp0.4-FtsZ complex. A mutant FtsZ variant (ftsZ9) that resists Gp0.4 binding abolishes defense activation, and other unrelated FtsZ inhibitors, such as SulA and Kil peptides, fail to trigger CapRelEbc, indicating that FtsZ inhibition alone is insufficient.

Mechanistic dissection through genetic screens, mutational analysis, structural modeling, and HDX reveals that Gp0.4 interacts with both FtsZ and the C-terminal antitoxin domain of CapRelEbc. Yeast two-hybrid assays demonstrate that CapRelEbc and Gp0.4 do not interact in isolation but only in the presence of FtsZ, removing the bacterial genetic background and providing additional confirmation of the ternary complex model.

Overall, this work is elegant and broadens the conceptual framework of bacterial immune sensing and draws interesting parallels to eukaryotic effector-triggered immunity (ETI). The findings suggest that searches for phage defense activators should consider host factors in addition to phage proteins and highlight the rapid functional diversification of related immunity proteins.

Major comments:

1. I believe the manuscript would be strengthened by additional biochemical characterization of the ternary complex in vitro. Since the major novelty of this work is in demonstrating recognition of a phage protein-host factor complex, direct biochemical validation of that complex would reinforce the conclusions. This could include, for example, HDX experiments omitting one component, binding affinity measurements, purification of the intact complex, or in vitro reconstitution assays such as pyrophosphorylation.

We have now added the following additional biochemical characterizations to show that Gp0.4 together with FtsZ can activate CapRel^{Ebc} *in vitro*.

1) Using purified CapRel^{Ebc}, FtsZ(L178E) (a monomeric variant of FtsZ), and Gp0.4 in a reconstituted transcription-translation system, we observed an inhibition of the production of a model protein DHFR only when all three proteins were present; omitting any one of the proteins abolished such inhibition (Fig. 4B). Further, no protein synthesis inhibition was observed using the L35A variant of Gp0.4 (L35 was mutated in our escape phages) (Fig. S5C).

2) We have performed ITC with purified proteins, and confirmed an interaction between FtsZ(L178E) and Gp0.4 (Fig. S3G). Importantly, we also observed interaction between CapRel^{Ebc} and the complex of FtsZ(L178E)-Gp0.4 by ITC (Fig. 4F), supporting the formation of a ternary complex.

These additional biochemical results, combined with our *in vivo* characterization, strongly support our model that Gp0.4 and FtsZ activate CapRel^{Ebc} via a ternary complex.

2. The data clearly demonstrate the necessity of FtsZ for the recognition of Gp0.4 by CapRelEbc. However, it is not entirely clear how FtsZ becomes essential for this recognition, as the AlphaFold3 model suggests only modest direct contacts between CapRelEbc and FtsZ. Could the binding of FtsZ instead stabilize the conformation of Gp0.4, making it more favorable for interaction with CapRelEbc?

We thank the reviewer for this suggestion. We think that FtsZ likely stabilizes the helical structures of Gp0.4, as has been seen with other peptide inhibitors of FtsZ such as MciZ (Bisson-Filho et al., 2015). We have now added additional data to support this idea: we show that Gp0.4 was destabilized (and therefore degraded) in the suppressor strain containing *ftsZ9* where Gp0.4 presumably no longer interacted with FtsZ (new Fig. 4H). This destabilization/degradation can be rescued by co-producing a monomeric FtsZ that interacts with Gp0.4 (Fig. 4H). Additionally, Gp0.4 variants that were unable to interact with FtsZ were found to be destabilized in cells as well (Fig. 3D, S4C). We now present and discuss these new analyses on lines 183-185 and lines 243-249 of the revised manuscript.

3. CapRelEbc and CapRelSJ46 are highly similar yet differ in binding specificity. Beyond the structural overlay in Fig. S5, a deeper analysis of sequence differences at the interaction interfaces would be helpful. Can the difference be rationalized by the amino acid changes between Ebc and SJ46 at the interaction residues with FtsZ, MCP, or Gp54? Could either protein recognize the other's known phage targets, depending on the identity of the infecting phage? If not, what prevents cross-recognition despite high sequence identity? If possible, it would also be valuable to expand the phage panel to determine whether CapRelEbc can confer defense against additional types of phages beyond T7.

We agree that it is very striking that these two highly similar proteins have different specificity with respect to phage recognition. We have previously shown that CapRel^{Ebc} cannot bind to either the MCP or Gp54 and is therefore unable to defend against these phages. We made chimeras of CapRel^{Ebc} and CapRel^{SJ46} by swapping their antitoxin regions, and found that the chimeric proteins switched their phage defense profiles, supporting the conclusion that the C-terminal antitoxin region involving the pseudo-ZFD functions as an infection sensor (Zhang et al., 2022; Zhang et al., 2024). We have now added a sequence alignment of CapRel^{Ebc} and CapRel^{SJ46} (Fig. S7), and commented that the antitoxin region is more different between these two proteins and the least conserved across diverse CapRel homologs (see lines 258-261). Pinpointing the exact subset of residues responsible for the different specificities of these homologs is, in our opinion, beyond the scope of this manuscript, but certainly an interesting angle to pursue in the future in the context of considering how phage recognition evolves.

Additional minor points:

4. It would be helpful to note in the manuscript that the FtsZ proteins from E. coli and E. chengduensis share 97% amino acid identity, suggesting that similar interactions would also be detected in the native host. Do the differing residues have any impact on interactions?

We thank the reviewer for pointing this out. Residues that are important for interacting with Gp0.4 (such as the synergy loop) are the same in these two proteins, so the interaction likely still holds in the native host. We now discuss this on lines 299-301.

5. Can the authors explain the apparent discrepancy between growth phenotypes in Fig. 1H (delayed growth over 2 hours) and Fig. S2A (no growth overnight) under the same induction conditions?

As mentioned below in response to reviewer #2 as well below: the OD_{600} measurements in Fig. 1H are, of course, a short-term assay that measures initial growth of the culture, with OD_{600} reflecting total cellular mass (not directly CFUs), whereas the spotting assay in Fig. S2A is an end-point assay after prolonged incubation and measures CFUs. Given that the effect of Gp0.4 is to inhibit cell division, its production initially leads to continued growth but substantial cell elongation (as shown by the microscopy in Figure 2A), hence the continued gain in OD_{600}; the extreme elongation, however, produces cells that are not viable in the long-term and so cannot form CFUs upon plating. This same phenomenon of continued growth in the short-term but with very limited plating viability in the long-term is also seen with other FtsZ inhibitors such as SulA, MinC, Era, and others (Gottesman et al *J Bact* 1981, Pichoff and Lutkenhaus *J Bact* 2001, Zhou et al *J Bact* 2020, Sanchez-Gorostiaga et al *mSphere* 2016). We provide additional data to support this hypothesis (see below in our response to reviewer #2) by comparing measured CFUs and calculated CFUs based on OD_{600}. We have also now commented on this issue in the text on lines 124-128.

6. The labeling in Figures 4C-D is somewhat difficult to follow. A bar plot similar to Fig. 3B might improve clarity.

We have improved the clarity of these figures (now as Fig. 4D-E) by putting labels directly next to the plates, and moved the +His plates to the supplement as suggested by reviewer #2.

7. Are there detectable differences in the FtsZ-Gp0.4 complex with and without CapRel present?

We did not see many differences in the FtsZ-Gp0.4 complex with and without $CapRel^{Ebc}$, which supports the hypothesis noted above that binding to FtsZ stabilizes Gp0.4. With the added experimental data, we now comment on this on lines 243-249.

Reviewer #2 (Remarks to the Author):

The manuscript "Activation of a bacterial immunity protein by a viral-host factor complex"

by Zhang et al. investigates a form of the phage defense protein CapRel and find that activation depends on the host protein FtsZ as well as the phage protein Gp0.4. In this paper the authors set out to characterize a form of CapRel (CapRelEbc) that is distinct from the previously characterized CapRel (CapRelSJ46) system that is activated via direct binding to phage capsid. They find that this system specifically defends against the phage T7. By generating phages capable of evading defense, the authors uncover that activation relies on the T7 protein Gp0.4. Gp0.4 has previously been described as a FtsZ inhibitor therefore raising the question, does CapRel activation have anything to do with Gp0.4 inhibition of FtsZ? Using diverse known FtsZ inhibitors the authors first demonstrate that CapRel activation is specific to Gp0.4-based inhibition and not simply general FtsZ inhibition, suggesting direct protein binding is involved. A series of point mutations within Gp0.4 are used in a both bacterial growth and yeast two-hybrid assays to begin building a model in which Gp0.4 binding to FtsZ alone doesn't trigger activation but that CapRel must also bind Gp0.4. The use of mutant forms of FtsZ and additional yeast two-hybrid assays supports a model in that Gp0.4, FtsZ, and CapRel form a ternary complex, and it is the formation of this complex that leads to activation of the toxic domain in CapRel and downstream phage defense. This is supported by an AlphaFold model which also provides an explanation for how the antitoxin domain is displaced through the binding of the FtsZ-Gp0.4 complex. Finally, the authors employ HDX-MS to support the proposed binding interface between the three proteins and how CapRel's confirmation is changed to trigger phage defense.
The findings from this work suggest an exciting mode of immune activation in bacteria; however, the manuscript lacks overall depth and the necessary controls/experiments to fully support the conclusions.

Major Issues:
- The primary issue this work is facing is the inherent toxicity imposed by Gp0.4. While this reviewer can appreciate the complications that come with working in such system, more controls are needed. The authors phrase the toxicity of Gp0.4 as "moderately toxic" (line 108); however, this is quite misleading. If you look at the CFU assays (Fig. S2a), there is complete elimination of colony formation. Further, if you dig into the methods the complex set up of the growth assay make is clear there is more than moderate toxicity. While this does not undermine the overall findings, additional data is needed to help support the genetic/growth data. This reviewers concern is that a sick bacterium behaves quite differently than a healthy one, especially when it comes to immune signaling which is impacted by cell "health". By saying there is "more toxicity" +/- the immune pathways could be due to numerous cell changes. Also, the likelihood that cultures are picking up mutations that elevate toxicity is high. There are a variety of different inducible system available that allow for more fine tune expression than arabinose and glucose-based systems. Can the authors use these other systems and find a level of expression where gp0.4 alone doesn't completely kill the bacterium? These could then be used in more standard growth assays (CFU assays that don't result in complete killing or growth curves that don't require such extensive manipulations)? Additionally, perhaps transformation efficiency assays can be employed against instead of standard plate spot assays.

Along with this, the change in methods and presentation style of growth curves in Fig. 3 makes it hard to compare with earlier data. For examples in Fig. 3 the impact of Gp0.4 of cell growth doesn't really manifest until later (8+ hrs) which isn't consistent with the early time point examine in Fig. 1 and 2.

As described in response to reviewer #1's minor comment on this same topic: The OD_{600} measurements in Fig. 1H reflect the short-term effects on cell mass accumulation, whereas the spotting/viability assay in Fig. S2A is an end-point assay after prolonged incubation and measures CFUs. Because Gp0.4 inhibits cell division, this leads to cell elongation, but continued growth in the short-term (as further shown by microscopy data in Fig. 2A); after longer periods of time, the extremely long cells have not divided to create new cells, hence the drop in viability in Fig. S2A. This same phenomenon is seen with other FtsZ/cell division inhibitors, as noted/referenced above.

To further validate our model for Gp0.4 and the consequences of its induction, we measured CFUs of cultures at 90 and 120 min post-induction (as in Fig. 1H) by plating cells on media containing glucose (see panel A below). We also plated the cells on arabinose-containing plates to verify there were no suppressors in the cultures. We measured CFUs in three independent experiments, and compared to the calculated CFUs based on the OD_{600} of the culture (see panel B below). There is a difference between the actual and calculated CFUs only in cells producing Gp0.4 (+ara), supporting the conclusion that OD_{600} measurements do not correctly reflect CFUs, and instead that OD_{600} increases because cells grow and elongate due to cell division inhibition by Gp0.4.

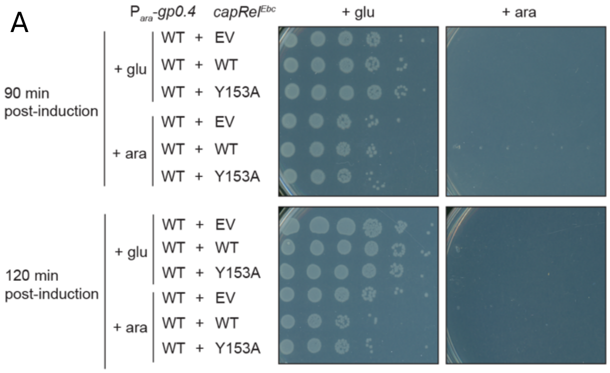

B

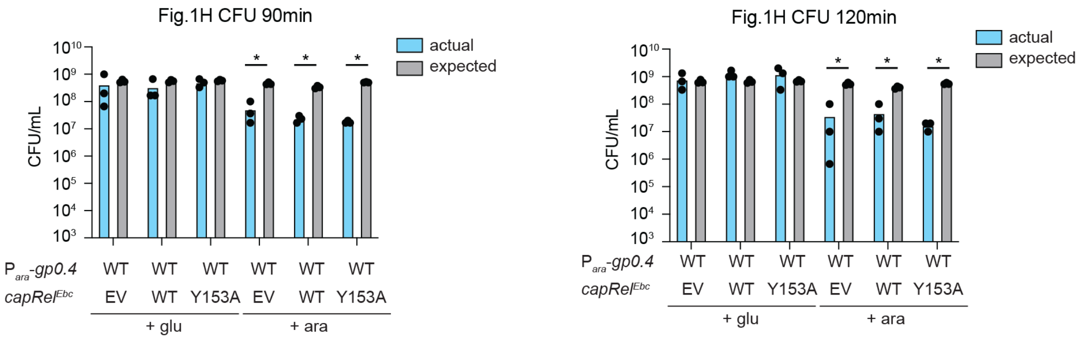

We appreciate the reviewer suggesting different induction methods (such as titrating down levels of arabinose in a relevant strain) to avoid toxicity. But the key finding of our work is that Gp0.4 must interact with FtsZ to activate CapRel^{Ebc} meaning it is not possible to separate the toxicity of wild-type Gp0.4 that results from FtsZ inhibition from the activation of CapRel^{Ebc}. In other words, if we titrate Gp0.4 levels down to a point where it no longer has any toxicity on its own, that means it's at a level where FtsZ isn't being bound, which means that it also won't activate CapRel^{Ebc}!

Further, we would emphasize that we had demonstrated that the additional toxicity associated with Gp0.4 induction required the enzymatic activity of CapRel^{Ebc}, as no toxicity was seen when co-producing Gp0.4 with the active site mutant CapRel^{Ebc} (Y153A) (Fig. 1H). And, perhaps most importantly: we showed that not all cell division inhibitors that lead to a 'sick cell' lead to additional toxicity when co-produced with CapRel^{Ebc} (Fig. 2E-G). These are extremely important controls that demonstrate that the activation is specific to Gp0.4 and not the general consequence of producing any cell division inhibitor.

As to the differences in Fig. 3 compared to Fig.1 and 2, this is because the data in Fig. 3 resulted from measurements made in a plate reader in a 96-well format starting from stationary phase cells, whereas data in Fig. 1 and 2 were from measurements made in shaking flasks starting from mid-exponential phase cells. To verify that the results are comparable, we measured growth of the Gp0.4 variants in Fig. 3 in the shaking flask set up (see data in new Fig. S4F-H).

Even in the yeast two hybrid assays there is a clear impact on cell toxicity in certain conditions. Again, while this doesn't completely undermine the findings it does complicate the findings, and this reviewer would like to see more in vitro confirmations. If this is not possible, at least in the yeast two hybrid, the catalytically dead point mutant of CapRel should be used to help reduce toxicity issues.

Neither Gp0.4 nor CapRel^{Ebc} was toxic to yeast, and we did not observe any toxicity in the yeast two hybrid assays, i.e. on non-selective media, there are no major differences in growth (non-selective conditions are in Fig. S4E and S5D-E of the revised figures). As mentioned in response to reviewer 1 and further articulated below in response to this reviewer's last 'Major' comment, we now also provide additional *in vitro* biochemical

experiments to confirm the formation of a ternary complex and the activation of $CapRel^{Ebc}$ by Gp0.4 and FtsZ.

- As a major focus of this paper is using point mutations to infer bindings site and disentangle complex toxicity issues, the expression level of the different protein variants needs to be explored through western blot analysis. Many of these results shown could simple be due to reduced or eliminated protein expression (especially the numerous mutants tested in Gp0.4 which is so small and likely easily to disrupt).

We thank the reviewer for suggesting this. For the Gp0.4 variants in Fig. 3H, we infer that they are not simply unfolded and degraded as they can still interact with and inhibit FtsZ. For the variants in Fig. 3E-F, we tested the expression levels of these proteins and found that these Gp0.4 variants do not accumulate to the same levels as wild-type Gp0.4 (Fig. S4C), likely because an interaction with FtsZ is important for stabilizing the helical structure of Gp0.4; a similar phenomenon has been seen with other peptide inhibitors of FtsZ such as MciZ (Bisson-Filho et al., 2015). We have also now added additional data to support this idea: we show that Gp0.4 was destabilized (likely degraded) in the suppressor strain containing *ftsZ9* where Gp0.4 presumably no longer interacts with FtsZ (new Fig. 4H). The reduction in Gp0.4 levels can be rescued when co-producing monomeric FtsZ that can interact with Gp0.4 (Fig. 4H). We have now modified the text to reflect these new data (see lines 183-187, 243-249).

- To help overcome the toxicity issue noted above and to help support the findings, can the authors perform more in vitro assays? The final piece of data using mass spec suggests that the authors are capable of purifying the individual components of this immune pathway (Gp0.4, FtsZ, and CapRel). The use of reciprocal IPs would help support interactions and solidify the dependency on Gp0.4 first binding FtsZ. Mutant forms of each protein could then be used in these assays to confirm in vivo findings. For example, it could be confirmed that the different point mutations in Gp0.4 truly impact the binding to one interacting partner but not the other. This could be taken one step further where the authors could show that the toxic domain of CapRel only is active in the presence of FtsZ (perhaps the monomeric mutant) and Gp0.4 using previous describe biochemical assays. This would really solidify the proposed model.

As noted in response to reviewer #1, we have now added the following additional biochemical data to show that Gp0.4 together with FtsZ can activate $CapRel^{Ebc}$ *in vitro*, which further supports our proposed model.

1) We incubated purified $CapRel^{Ebc}$, FtsZ(L178E) (a monomeric variant of FtsZ), and Gp0.4 in a reconstituted transcription-translation system, and observed inhibited production of a model protein DHFR only when all three proteins were present; omitting any single protein abolished such inhibition (Fig. 4B). No protein synthesis inhibition was observed using the L35A variant of Gp0.4 (L35 was mutated in our escape phages) (Fig. S5C).

2) We have performed ITC with purified proteins, and confirmed an interaction between FtsZ(L178E) and Gp0.4 (Fig. S3G). Importantly, we also observed interaction between

CapRel^{Ebc} and the complex of FtsZ(L178E)-Gp0.4 by ITC (Fig. 4F), supporting the formation of a ternary complex.

These additional biochemical results, combined with our *in vivo* studies, strongly support our model that Gp0.4 and FtsZ activate CapRel^{Ebc} via a ternary complex.

Minor Issues:
- Representation of data lacks transparency and doesn't emphasize relevant comparisons. Throughout the manuscript the way the data is presented makes it challenging for the reading to interpret. Some examples below:

o Fig. 1A; the use of heatmap obscures the variance within biological replicates and as there is only a single defense system shown there aren't so many comparisons that a bar graph would be overwhelming.

As suggested, bar graphs summarizing 3 independent biological replicates are now included in Fig. S1B.

o The majority of phage protection is shown as plate images. While this nicely demonstrates the phenotype, a paired quantification of three biological replicates should be included. Especially in systems with toxicity, the variance between experiments needs to be shown.

We prefer showing raw plate images for phage protection assays as it shows both the PFU and plaque sizes, but as suggested, we now also included bar graphs quantifying each of the spotting assays with 3 biological replicates (Fig. S1B, S1C, S1E, S1H, S2B).

o Growth curves are only two replicates and lack any statistics. When the differences are as small as in Fig. 1H, a full three replicates and stats should be shown to help support conclusions.

We have now included 3 biological replicates for growth curves shown in Fig. 1H, 2C, 2E, 2F, 2G, and statistics have been added for Fig. 1H where a difference was seen. The additional repeats have not changed any of the original conclusions.

o There are many places where the control data should be moved to the supplement to help the reader interpret findings. Currently there is often just too much data to weed through to find the relevant comparison. For example: throughout Fig. 3; the +glucose plates can be moved to the supplement to help focus the reader on when gp0.4 is present. For the Y2H; the +His plate can also be moved and in place labels can be places on the - His plate to make it easier to read (the current key is very confusing)

As suggested, the +glucose plates in Fig. 3 and the +His plates of Y2H in Fig. 4 have been moved to the supplement. We now place labels directly on the -His plates to improve clarify as suggested.

o The microscopy is really hard to see (very faint and lacks contrast) and also lacks necessary controls (no gp0.4)

We have improved the contrast of the microscopy images and included scale bars. Since Gp0.4 is under arabinose-inducible promoter and otherwise suppressed with glucose, the t = 0 min is the control for cells without Gp0.4 expression.

o Comparing the views between Fig. 3D and Fig. 3G is impossible and this reviewer would like to see how the structure is rotated to help see where the two sets of mutations are in comparison to each other.

We have now added a symbol indicating the relative rotation of Fig. 3D and Fig. 3G.

- With the high degree of specificity seen for this system (so specific for T7) it would be interesting to explore the conservation and distribution of the combination. Both on the side of the phage and on CapRel. The authors start to get at how CapRel proteins can recognize diverse substrates in the discussion, but can there be more? How different is CapRelEbc from CapRelSJ46? Are there more types? How different are these? Not necessary but would help but these findings in the context of the larger field. On the side of the phage, for only T7 Bas64 and Bas65 encode homologs? Are there any related ones out there?

As noted above in response to reviewer #1, we have previously shown that $CapRel^{Ebc}$ cannot bind to either the MCP or Gp54 that activate $CapRel^{SJ46}$, and so is unable to defend against the phages encoding those activators. We made chimeras of $CapRel^{Ebc}$ and $CapRel^{SJ46}$ by swapping their antitoxin regions, and found that the chimeric proteins switched their phage defense profile, supporting the conclusion that the C-terminal antitoxin involving the pseudo-ZFD functions as an infection sensor (Zhang et al., 2022; Zhang et al., 2024). We have now added a sequence alignment of $CapRel^{Ebc}$ and $CapRel^{SJ46}$ (Fig. S7), and commented that the antitoxin region is more different between these two proteins and is the least conserved region across diverse CapRel homologs (see lines 258-261).

We did not find any other phages that encode Gp0.4 homologs, but it is possible that $CapRel^{Ebc}$ can sense other phage-encoded triggers that are not related to Gp0.4, as seen with $CapRel^{SJ46}$, which can bind two unrelated proteins (the MCP and Gp54).

- Why were all Gp0.4 escapee phages in the FtsZ binding site? Wouldn’t it be more advantages for the phages to generate mutations like those the authors generated in Fig. 3G? As the authors state, there is a fitness advantage for phages to inhibit FtsZ, so why weren’t these variants select for? At minimal this point should be discussed.

In addition to the mutants that map to the FtsZ binding site, we also isolated phage escapers that have a frameshift in Gp0.4 (Fig. 1E, S1F). Why we did not get mutations like those in Fig. 3G is not clear. Obviously our escape screen was not done to saturation so other mutations may have arisen if we continued screening. Whether there is a significant enough fitness disadvantage in our growth and plaquing conditions following loss of Gp0.4 function is not clear. There are also potentially other trade-offs introduced by various mutations that influence their frequencies, as well as possible non-uniformities in

mutation rates across phage genes. Given so much uncertainty about why our limited escape screen only produced two substitutions mapping to the FtsZ binding surface and a frameshift, we are hesitant to speculate on this topic in the manuscript.

- In Fig. 1H the bottom dot in the key has a green outline but blue fill. I think it should all be green.

We thank the reviewer for pointing this out - this has now been fixed.

- Authors should consider de-emphasizing the comparison to effector triggered immunity and this model more closely resembles a form of PAMP detection. ETI really relies on the immune system monitoring an output that is changed upon infection which doesn't fit with Gp0.4.

We have removed one case in the Discussion where we agree too strong a comparison was being made, but we retain some of the general commentary on this point. We think the commentary is explicit about the fact that Gp0.4 does not perfectly adhere to the ETI framework. For instance, on lines 317-320 we note that "Our studies indicate that the activation of $CapRel^{Ebc}$ shares features of both PAMP and ETI-based strategies, binding to the T7 phage protein Gp0.4, but only once this phage-encoded effector has bound to monomeric FtsZ to inhibit cell division" and later on lines 326-328 that "Although akin to ETI in some ways, $CapRel^{Ebc}$ does not solely sense the consequences of cell division inhibition by Gp0.4 as evidenced by our finding that other FtsZ inhibitors, such as SulA and the Kil peptides, do not trigger $CapRel^{Ebc}$ activity." We think the comparing and contrasting to eukaryotic concepts is useful with the clear differences being explicitly discussed.

Reviewer #3 (Remarks to the Author):

The manuscript by Zhang et al. describes a previously described tRNA pyrophosphorylase anti-phage defense system, CapRel(Ebc), and shows that it is a single-component TA system with both toxin and antioxin components. The CapRel(Ebc) protein is shown to be active against phage T7, and genetic studies show that it is activated by the presence of T7 gp0.4, an inhibitor of cell division. Additional genetic studies show that the interaction of gp0.4 with host FtsZ is required for activation of CapRel(Ebc), as point mutations in gp0.4 or FtsZ that reduce the interaction are protective. The findings are supported by site-directed mutagenesis based on predicted structures and yeast two-hybrid studies. A model is presented in which the gp0.4-FtsZ complex is required for activation of toxin activity.

The manuscript is generally well-written and well-presented, and in the absence of direct (e.g., structural) data, lays out a strong case in support of the model based on genetic experiments. Comments are noted below.

Abstract: the abstract should not contain citations.

Citations have been removed from the abstract.

The growth defects observed in liquid culture seem to variy between experimental systems. For example, in Fig. 1h expression of gp0.4 produces a mild growth defect but completely eliminates colony formation in Fig. 3c. How do you reconcile these differences in observation between approaches?

Please see our responses to reviewer 1 (minor point #5) and reviewer 2 (point #1) on this same issue.

Fig. 1e: indicate multiple identical frameshifts were isolated.

We have now clarified the frameshifts were identical in lines 91-92.

Fig. 1f: the labeling within the genes is difficult to read. What is represented by the dashed line?

We have improved the clarity of Fig. 1F by using a different approach, which now highlights the homologous genes across these phages.

Fig. 1h: here and elsewhere, the yellow line and markers are very difficult to see, suggest use of an alternate color. Specify the meaning of EV in the caption here and elsewhere.

We have changed the yellow to orange line across the paper, and specified the meaning of EV.

Line 94: Can these all be called loss of function mutations?

As suggested, we have removed the 'loss of function'.

Fig. 2a: micrographs require scale bars. Zooming in on the cells and increasing the image contrast would help show the differences between conditions. Also specify this is phase-contrast microscopy in the figure caption.

Scale bars have been added, and the image contrast has been adjusted. We also now specify it is phage-contrast microscopy.

Fig. 2d: there appears to be a plaque at the 10^-3 dilution of the wt strain expressing CapRel. Is this an anomaly or a repeatable observation?

We believe this is an anomaly, and we have now replaced the figure with a different experiment. All spotting assays have been done 3 times independently, and we now added quantification in Fig. S2B.

Fig. 3i specifies means of 7-10 "plate replicates" are shown, are these true biological replicates or subsamples of the same replicate? All experiments should be performed in biological replicates.

They are plate replicates from the same culture, but the entire experiments have been repeated at least twice. We have now clarified this in the caption.

We thank the reviewers for their final comments.

Reviewer #1 (Remarks to the Author):

The authors have thoroughly and thoughtfully addressed my previous concerns. The inclusion of new in vitro biochemical data significantly strengthens the central claim of the paper that CapRelEbc activation requires a ternary complex with both Gp0.4 and FtsZ. Overall, the revised manuscript provides a compelling, well-supported mechanism for this unique defense system.

I have only one minor suggestion for the final text. Since ITC is the biochemical method used that definitively establishes the binding stoichiometry, I recommend adding a brief statement around lines 234-235 to point out the 1:1:1 molar ratio of the ternary complex derived from the ITC curves.

Congratulations to the authors on an excellent study.

We have made this update to the text, noting the 1:1:1 ratio of the ternary complex.

Reviewer #3 (Remarks to the Author):

I thank the authors for their revisions to the manuscript, including additional text and modifications to figures. I have no additional revisions.
